# Efficacy of hormone therapy and phytoestrogens on the psychological symptoms of menopausal women: a systematic review, meta-analysis, and trial sequential analysis

**DOI:** 10.3389/fmed.2026.1855845

**Published:** 2026-07-10

**Authors:** Mengna Shou, Mengfei Ye, Chunhua Lou, Xinyi Chen, Yiying Yao, Junwei Yan, Wenchao Zhou, Zimu Fu, Zheng Liu

**Affiliations:** 1 Department of Women's Health, Shaoxing Maternity and Child Healthcare Hospital, Maternity and Child Healthcare Affiliated Hospital, Shaoxing University, Shaoxing, Zhejiang, China; 2 Department of Psychiatry, Shaoxing Seventh People's Hospital, Affiliated Mental Health Center of Shaoxing University, Shaoxing, Zhejiang, China; 3 Shaoxing Key Laboratory of Diagnosis and Treatment Psychiatic Disorders, Shaoxing, Zhejiang, China; 4 Department of Pharmacology, School of Medicine, Shaoxing University, Shaoxing, Zhejiang, China; 5 Department of Blood Transfusion, Affiliated Hospital of Shaoxing University, Shaoxing, Zhejiang, China; 6 Department of Gynecology, Cixi People's Hospital, Wenzhou Medical University, Ningbo, Zhejiang, China

**Keywords:** hormone therapy, menopause, meta-analysis, phytoestrogens, psychological

## Abstract

**Background:**

The efficacy of hormone therapy and phytoestrogens in reducing psychological symptoms of menopausal women has generated substantial debate. We aimed to evaluate the effectiveness of hormone therapy and phytoestrogens for psychological symptoms.

**Methods:**

We conducted a comprehensive search of multiple academic databases, including EMBASE, Cochrane Library, Web of Science, PubMed, and PsycINFO. The inclusion criteria were limited to randomized controlled trials (RCTs) published up to March 1, 2026. Hedge's *g* was utilized as the standardized between-group effect size, and a random-effects model was applied. Trial sequential analysis was performed to gauge the statistical reliability of the data in the cumulative meta-analysis.

**Results:**

We analyzed the participant data from 51 RCTs of hormone therapy (*n* = 41,821) and 16 RCTs of phytoestrogens (*n* = 1,191). The study found hormone therapy and phytoestrogens were associated with the alleviation of psychological symptoms in menopausal women, particularly in mood and anxiety. Trial sequential analysis confirmed the robustness of those findings, while some significant outcomes failed correction and require further validation. In addition, Tibolone showed a significant improvement in depression (SMD 0.93, 95% CI 0.62–1.24). Compared with perimenopausal women, post-menopausal women showed a greater beneficial effect of hormone therapy on sleep quality and of phytoestrogens on depression and anxiety.

**Conclusion:**

Hormone therapy and phytoestrogens alleviated psychological symptoms in menopausal women, but their effects differed by symptoms. The therapeutic strategy should be adopted based on individual patient's specific characteristics.

**Systematic review registration:**

https://www.crd.york.ac.uk/PROSPERO/, Identifier: CRD42024533658.

## 1 Introduction

Menopause has been identified as a recognized risk factor for psychological issues in women, with up to two-thirds of women experiencing mild to moderate psychological symptoms during menopause (1). Menopause-related psychological symptoms decrease the quality of life (QoL) and overall life satisfaction, resulting in significant individual, family, and society burdens (2). There is an interdependent relationship between sleep disturbances and depression during menopause, as the former can significantly increase the likelihood of experiencing depressive symptoms by two to three times (3). The implementation of measures aimed at effectively managing menopause can enhance the QoL of women.

Analysis of existing clinical evidence has demonstrated that estrogen, phytoestrogens, selective serotonin reuptake inhibitors (SSRIs), and cognitive behavior therapy have beneficial effects on the psychological health of menopausal women (4, 5). However, the treatment of menopausal symptoms with SSRIs is controversial because their primary adverse events are dry mouth, nausea, constipation, and reduced libido, and they may cause or worsen insomnia, despite providing relief for depression (6). Previous studies on the impact of estrogen treatment on the psychological health of menopausal women have been inconclusive, as certain studies have reported a decrease in depression, anxiety, and sleep latency, whereas others have found no discernible effect (7, 8). Phytoestrogens are naturally occurring compounds found in plants that can serve as viable substitutes for estrogens (9). However, it is unclear whether phytoestrogens affect psychological symptoms directly or indirectly. These issues could be resolved by conducting a well-conducted meta-analysis of randomized controlled trials (RCTs).

Several previous meta-analyses have evaluated the effect of hormone therapy and phytoestrogens on the psychological health of menopausal women (10, 11). However, many of these studies assessed the outcome of only one symptom, such as depression, sleep disorders, or anxiety. In addition, potentially important between-study differences remained unexplored, including participants and intervention duration. Additionally, meta-analyses are inherently limited by the risk of type I error. It has been reported that 7% of Cochrane reviews have reached false-positive conclusions, and 93% of these errors could have been avoided by applying trial sequential analysis (TSA) (12). TSA operates on principles similar to interim analysis in RCTs. When the required information size is not achieved, TSA increases the uncertainty around the intervention effect estimate, thereby reducing the probability of type I error (13).

Therefore, the primary objective of this study was to conduct a systematic review of clinical trials and integrate the available evidence with a meta-analysis to compare the therapeutic advantages of various interventions in addressing psychological symptoms among menopausal women. The secondary aim was to investigate the possible influence of between-study differences in different reproductive states, therapeutic regimens, and duration of intervention. We also evaluated the adverse events of hormone therapy and phytoestrogens. Additionally, we employed TSA to assess how pre-mature conclusions could be avoided.

## 2 Methods

This systematic review and meta-analysis was conducted strictly following the Cochrane Handbook for Systematic Reviews of Interventions (14). The reporting of this study was performed in accordance with the Preferred Reporting Items for Systematic Reviews and Meta-Analyses (PRISMA) statement (15). This study has been registered in PROSPERO (registration number: CRD42024533658).

### 2.1 Search strategy

We performed keyword-based searches of the EMBASE, Cochrane Library, Web of Science databases, PubMed, and PsycINFO. A combination of keywords was used to indicate the psychological health of menopause, including depression, anxiety, stress, anger, and hormone therapy or phytoestrogens, such as estrogen, hormone replacement, phytoestrogen, and phyto-estrogen. March 1, 2026, was the end date of the literature search, which included forward searches (citation tracking) and backward searches (snowballing) of systematic reviews and identified studies. Detailed search strategies are presented in Supplementary Tables 1, 2.

### 2.2 Selection procedure

The inclusion of English articles was limited to peer-reviewed publications. We included RCTs that analyzed the effects of hormone therapy and phytoestrogens on menopausal symptoms and provided outcome data for calculating effect sizes. The PICO (population, intervention, comparison, and outcome) principle was used to assess the articles' eligibility (16).

The inclusion criteria were as follows: population, women who were in perimenopause and post-menopause and/or had treatment-induced menopausal symptoms; intervention, hormone therapy and phytoestrogens; comparison, a control group was required for eligible studies, Including placebo, calcium, and no treatment; outcome, questionnaire results of psychological health during menopause, e.g., depression, sleep disorders, anxiety, stress, and anger.

### 2.3 Extraction and analysis of the data

Two authors (MY and ZF) worked in pairs to eliminate duplicates and screen the records to ensure that each record was independently evaluated by two investigators. After screening the titles and abstracts of the articles, the complete texts of the remaining articles were assessed, and the rationales for excluding articles were documented. Whenever there was a disagreement, a third author (XC) was involved until a conclusion was reached. One author (MY) extracted data from the included studies and another (MS) checked them. Additionally, adverse events associated with all included studies were recorded, including weakness, gastrointestinal symptoms, irregular vaginal bleeding, and peripheral edema. Adverse events were systematically categorized according to the Medical Dictionary for Regulatory Activities (MedDRA) version 20.0.

Pooled standardized mean differences (SMD) were calculated using random-effect models. Each outcome was standardized using Hedge's *g*, a variation of Cohen's *d*, and the 95% confidence interval (CI) was computed. The pooled effect sizes were aggregated from baseline to post-intervention, and the final quantitative data synthesis incorporated the number of participants included in the calculation. The random-effects model was used because of the anticipated clinical and methodological heterogeneity of treatment effects across the studies. Effect sizes were adjusted by their opposite number for some symptoms, with positive values indicating a better outcome after the intervention. The effect sizes were interpreted as either small (0.2), moderate (0.5), or large (0.8).

### 2.4 Heterogeneity

Studies were analyzed for heterogeneity using the *I*^2^ and Q statistics. *I*^2^ calculated the difference between effect estimates caused by between-study heterogeneity instead of chance (17). *I*^2^ values of 25, 50, and 75% were considered heterogeneous to low, moderate, and high degrees, respectively. Significant heterogeneity was determined by a *P*-value of 0.10. Sensitivity analysis was conducted to address treatment heterogeneity across the included studies. Subgroup analyses were performed to determine the origins of heterogeneity, employing a mixed-effects model to aggregate studies within a subgroup and assess statistically significant variations between subgroups. Moreover, meta-regression was used to evaluate the impact of differences in the characteristics of studies on the treatment effect.

### 2.5 Risk of bias assessment

An adaptation of the Cochrane Collaboration tool (18) was developed to assess the risk of bias in intervention studies. The risk of bias included the following seven domains, “random sequence allocation”, “allocation concealment”, “blinding of participants and personnel”, “blinding of outcome assessment”, “incomplete outcome data”, “selective reporting”, and “other bias”. The risk of bias in each study was evaluated independently by two authors (MY., MS), and differences were discussed with the third author (ZL) until an undisputed rating was obtained. To determine the reliability of independent ratings, inter-rater agreement, and Kappa statistics were used before the final score was determined. A Funnel plot was used to estimate the publication bias, which was quantified using the Egger's test. Using the Duval and Tweedie trim and fill procedure, the effect sizes were corrected for potential publication bias, and an estimate of the number of studies that might have been overlooked was included (19). Statistical analyses were conducted using Stata version 16.0 and R version 4.3.3.

### 2.6 Trial sequential analysis

TSA was performed using TSA program version 0.9.5.10 beta. A sufficient level of evidence for the anticipated intervention effect was reached and no further trials were needed if the cumulative Z-curve crossed the trial sequential monitoring boundary or entered the futility area, whereas if the Z-curve did not cross any of the boundaries or the required information size (RIS) has not been reached, the evidence of the conclusion was considered to be insufficient and more trials were needed to confirm the results. We used a random-effects model with CIs of 95%, an information axis with sample size, type 1 error of 2-sided boundary type of 5%, and power of 80%.

## 3 Results

### 3.1 Selection and inclusion of studies

**Figure 1:**
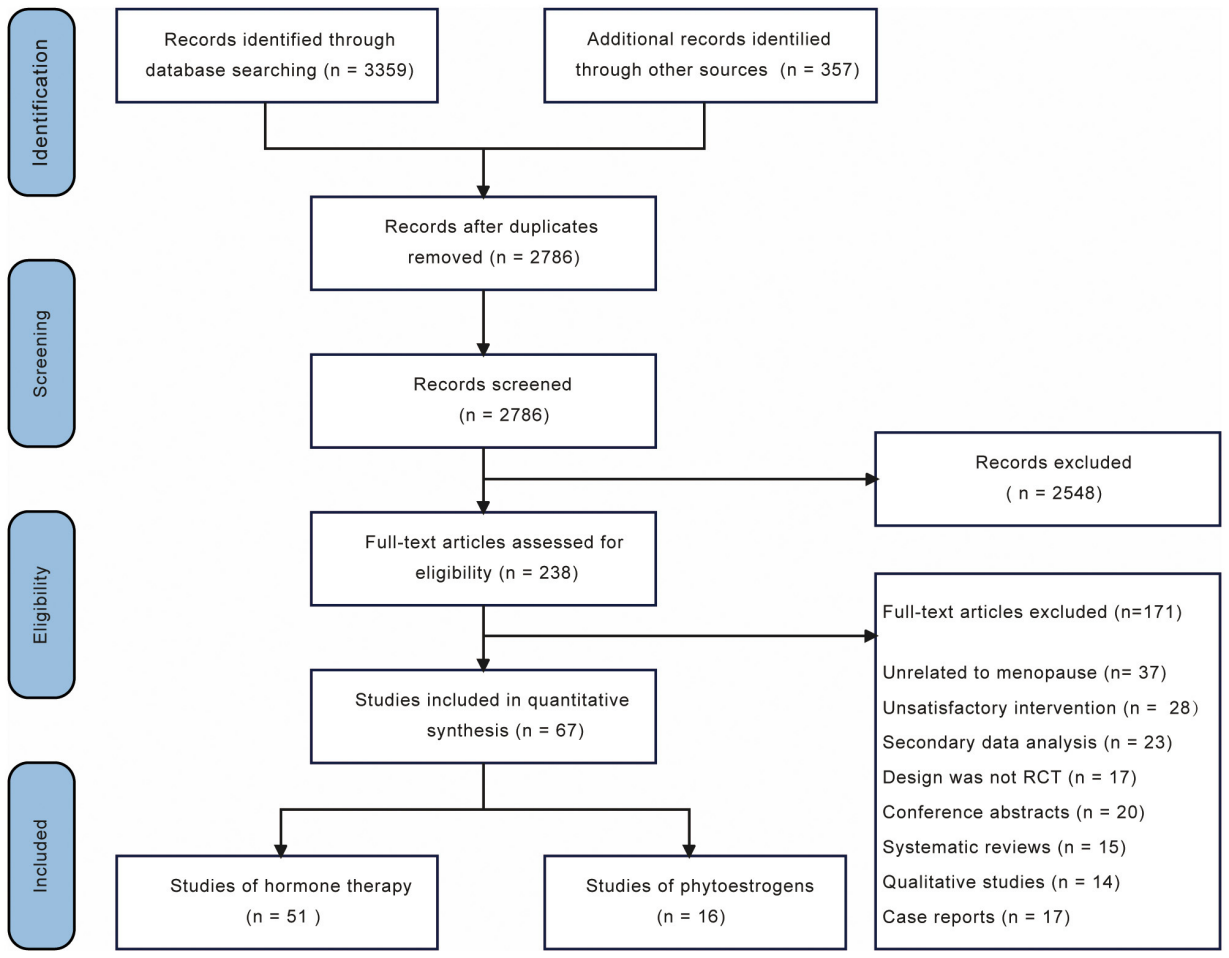
PRISMA flowchart of the study selection. Flowchart summary of the study selection process (adapted from PRISMA guidelines).

A total of 3,716 articles were examined (2,786 after removing duplicates), and 238 full-text articles were included for further evaluation. As a result, 171 articles were excluded for the following reasons: unrelated to menopause (*n* = 37), unsatisfactory intervention (*n* = 28), secondary data analysis (*n* = 23), not RCT (*n* = 17), conference abstracts (*n* = 20), systematic reviews (*n* = 15), qualitative studies (*n* = 14), and case reports (*n* = 17). The remaining 67 RCTs (43,012 patients) were included in this meta-analysis (Figure 1). Among the excluded literature, 37 studies superficially described menopause in the abstract, yet their actual study populations failed to satisfy the standard diagnostic requirements for both natural menopause and treatment-induced menopause, and were therefore excluded from the final quantitative synthesis.

### 3.2 Characteristics of the included studies

A summary of the characteristics of the included studies is shown in Tables 1, 2. Of the 67 RCTs hormone therapy was employed as an intervention in 51, whereas phytoestrogens were used in 16 (7, 8, 20–84). To determine the impact of reproductive state, the populations under investigation were further classified into perimenopausal and post-menopausal categories. In most studies, participants of the included studies were post-menopausal women (69.23%). The administration for drug delivery was oral in almost all studies (83.07%). More detailed characteristics of the included studies are provided in Supplementary eTable 3.

**Table 1:** Information of the studies that were included.

References	Country	Sample size	Dropout (%)	Participants	Age (mean ±SD)	Intervention	Route of administration	Control	College degree or higher (%)	Duration (weeks)	Outcome measures
Schmidt et al. (8)	USA	46	8.0	Peri-M	50.2 ± 3.3	Transdermal 17β-estradiol 100 ug/d, Phytoestrogen compound Rimostil (2 g/d)	Transdermal Oral	Placebo	N	8	HAMD, VAS
Liu et al. (84)	China	152	0.0	Perimenopausal	54.6 ± 4.1	Oral EPT (E2 1 mg/ d + MPA 8 mg/d)	Oral	Placebo	38.16	12	SRSS, PANAS
Brunner et al. (25)	USA	10,730	10.7	Post-M	63.6 ± 4.0	Oral estrogen (CEE 0.625 mg/d)	Oral	Placebo	24.70	144	WHIIRS, RAND36
Chen. (37)	China	180	7.7	Perimenopausal	49.7 ± 2.9	Oral EPT (E2 1 mg/ d + MPA 10 mg/d)	Oral	Placebo	34.00	12	PSQI, ISI
Gülseren et al. (28)	Turkey	42	4.8	Post-M	48.7 ± 3.6	Tibolone (2.5 mg/d)	Oral	Placebo	N	24	HAMA, HAMD
Ensrud et al. (83)	USA	224	8.5	Peri- and post-M	54.6 ± 3.8	Oral estrogen (E2 0.5 mg/d)	Oral	Placebo	51.03	8	PSQI
Gambacciani et al. (55)	Italy	50	0.0	Post-M	54.4 ± 2.5	Oral EPT (E2 1 mg/d + NETA 0.5 mg/d)	Oral	Calcium-vitamin	18.00	12	WHQ
Block et al. (20)	USA	21	0.0	Peri-M	48.5 ± 6.6	Progesterone (MPA30 mg/d)	Oral	Placebo	N	10	TST
Zhang et al. (40)	China	100	0.0	Peri- and post-M	52.1 ± 6.3	Oral estrogen (E2 2 mg/d)	Oral	Placebo	N	12	PSQI, HAMA, HAMD, MENQOL
Gambacciani et al. (56)	Italy	40	0.0	Post-M	53.4 ± 2.6	Oral EPT (CE 0.3 mg/d + MPA 2.5 mg/d)	Oral	Calcium	N	12	WHQ
Gambacciani et al. (31)	Italy	52	0.0	Post-M	52.7 ± 2.8	Oral EPT (E2 1 mg/d + drospirenone 2 mg/d)	Oral	Calcium	N	12	WHQ
Caan et al. (66)	USA	243	0.0	Post-M	54.6 ± 3.8	Oral estrogen (E2 0.5 mg/d)	Oral	Placebo	50.70	8	PHQ-9, GAD-7, PSS, MENQOL
Hays et al. (73)	USA	16608	7.9	Post-M	63.2 ± 3.1	Oral EPT (CEE 0.625 mg/d + MPA 2.5 mg/d)	Oral	Placebo	35.30	52	QOL, CES-D, WHIIRS
Heinrich et al. (75)	Germany	35	12.5	Post-M	64.1 ± 1.8	Oral estrogen (EV 2 mg/d), Oral estrogen (EV2 mg + Progesterone 100 mg/d)	Oral	Placebo	N	24	Sleep quality, MMSE, CES-D
Joffe et al. (46)	USA	41	14.6	Peri-M	51.1 ± 5.0	Oral estrogen (E2 0.05 mg/d)	Oral	Placebo	67.60	8	PSQI, MADRS
Joffe et al. (46)	USA	41	14.6	Peri-M	51.1 ± 5.0	Oral estrogen (E2 0.05 mg/d)	Oral	Placebo	67.60	8	PSQI, MADRS
Diem et al. (67)	USA	198	1.3	Post-M	61.0 ± 4.0	Estradiol 10 mg/d	Vaginal	Placebo	66.30	12	MENQOL, PHQ-9, GAD-7
LeBlanc et al. (68)	USA	32	0.0	Peri- and post-M	53.2 ± 2.3	Oral estrogen (2 mg/d)	Oral	Placebo	N	8	Sleep diary, POMS
Khanna et al. (53)	India	42	12.5	Peri-M	45.2 ± 3.8	Fenugreek seeds 250 mg/d	Oral	Placebo	53.10	6	MRS
Meeuwsen et al. (57)	Holland	81	5.9	Post-M	54.2 ± 4.7	Tibolone (2.5 mg/d)	Oral	Placebo	N	48	NHP
Nielsen et al. (58)	Denmark	232	20.0	Post-M	52.6 ± 1.6	Intranasal estrogen (S21400 150 mg/d)	Intranasal	Placebo	N	104	WHQ
Cagnacci et al. (59)	Italy	43	8.4	Peri-M	50.2 ± 0.6	Phytoestrogen Kava-Kava 200 mg/d	Oral	Placebo	N	12	STAI, SDS
Purdie et al. (60)	UK	33	0.0	Post-M	54.3 ± 3.8	Oral EPT (CEE 0.625 mg/+ Norgestrel 0.15 mg/d)	Oral	Placebo	N	12	CCEI, SSQ
Hirose et al. (24)	Japan	58	3.0	Peri- and Post-M	48.0 ± 5.7	Phytoestrogen isoflavone 25 mg/d	Oral	Placebo	N	8	HADS, AIS
Saletu-Zyhlarz et al. (49)	Austria	49	0.0	Post-M	58.0 ± 5.0	Oral estrogen (EV 2 mg/d), Oral EPT (EV 2 mg/d + Dienogest 3 mg/d)	Oral	Placebo	N	8	PSQI
Schüssler et al. (80)	Germany	20	0.0	Post-M	60.3 ± 5.7	Oral progesterone (300 mg/d)	Oral	Placebo	N	3	TST
Silva et al. (26)	Brazil	12	0.0	Post-M	47.7 ± 4.4	Oral EPT (E 1 mg/+ trimegestone 0.125 mg/s)	Oral	Placebo	N	4	PSQI
Sismondi et al. (61)	Multi-nations	3133	1.1	Post-M, patients with breast cancer	52.7 ± 7.3	Tibolone 2.5 mg/d	Oral	Placebo	N	104	WHQ
Ishiwata et al. (69)	Japan	37	10.8	Peri- and Post-M	50.5 ± 4.7	Phytoestrogen Equol 10 mg/d	Oral	Placebo	N	12	POMS
Welton et al. (30)	UK NZ Australia	3721	42.8	Post-M	63.8 ± 4.4	Oral EPT (CEE 0.625 mg + MPA 2.5/5.0 mg)	Oral	Placebo	N	52	WHQ, CES-D
Carranza et al. (42)	Mexico	12	0.0	Hysterectomized post-M	49.1 ± 5.6	Oral estrogen (CEE 0.625 mg/d)	Oral	No treatment	N	24	HAMD
Hachul et al. (70)	Brazil	38	36.7	Post-M	57.4 ± 5.2	Isoflavones 80 mg	Oral	Placebo	N	16	TST
Almeida et al. (74)	Australia	86	25.2	Post-M	73.7 ± 4.5	Oral estrogen (E2 2 mg/d)	Oral	Placebo	36.70	20	BDI, BAI, SF36
Bech et al. (82)	Denmark	67	33.6	Post-M	53.1 ± 6.2	EPT (E2 2 mg/+ 1 mg NETA)	Oral	Placebo	N	48	BDI, GHQ
Demetrio et al. (27)	Brazil	66	0.0	Post-M	50.8 ± 2.7	estrogen (CEEs 0.625 mg/d)	Oral	Placebo	N	24	BDI, BAI, POMS, STAI
Girdler et al. (51)	USA	54	0.0	Post-M	52.3 ± 5.9	Oral estrogen (CEE 0.625 mg/d), Oral EPT (CEE 0.625 mg/d + MPA 10 mg/d)	Oral	Placebo	43.43	24	BDI, POMS, STAI
Haines et al. (62)	China (Hong Kong)	100	9.0	Post-M	56.7 ± 5.6	Oral estrogen (E2 2 mg/d)	Oral	Placebo	N	48	HADS, WHOQOL
Hlatky et al. (52)	USA	2246	18.7	Post-M	66.6 ± 6.7	Oral EPT (CEE 0.625 mg/d + MPA 2.5 mg/d)	Oral	Placebo	37.10	144	RAND36
Khoo et al. (32)	Australia	80	17.0	Peri-M	46.1 ± 2.9	Oral EPT (CEE 0.625 mg/d + MPA 10 mg/d)	Oral	Placebo	N	48	ZSRD, STAI, SRSD
Pearce et al. (48)	UK	40	0.0	Post-M	50.1 ± 6.4	E2 50 mg/d	Implant	Placebo	N	8	HADS
Baksu et al. (63)	Turkey	133	0.0	Surgically M	49.8 ± 4.8	Oral estrogen (CEE 0.625 mg/d), Intranasal 300 ug/d estradiol hemihidrate, Transdermal l1.5 mg/d estradiol hemihidrate	Oral Intranasal Transdermal	Placebo	N	48	HAMA, HAMD
Frigo et al. (45)	Brazil	43	10.4	Peri-M	50.4 ± 5.2	40 mg of soybean and 40 mg flaxseed phytoestrogen	Oral	Rice flakes biscuit	34.88	22	KI
Rasgon et al. (54)	USA	22	0.0	Post-M	55.5 ± 6.4	Transdermal estrogen 0.1 mg/d	Transdermal	Placebo	N	10	HAMD
Zanardi et al. (39)	Italy	170	7.6	Post-M	54.5 ± 5.0	Oral EPT (CEE 0.611 mg/d + MPA 5 mg/d)	Oral	Placebo	N	7	HAMD
Paoletti et al. (47)	Italy	60	0.0	Post-M	52.3 ± 2.1	Transdermal 0.05 mg/d estrogen, Oral EPT (E2 2 mg/d + MPA 1 mg/d)	Transdermal Oral	Placebo	N	12	SCL-90
Morrison et al. (29)	USA	55	3.5	Post-M women diagnosed with depressive disorders	61.9 ± 9.4	Transdermal E2 1 mg/d	Transdermal	Placebo	65.40	8	HAMD
Rudolph et al. (33)	Germany	89	31.0	Post-M women with depressive episodes	56.1 ± 5.1	Oral EPT (E2 2 mg + dienogest 2 mg)	Oral	Placebo	N	24	HAMD
Schmidt et al. (21)	USA	34	11.8	Peri-M	49.3 ± 2.9	Oral EPT (E2 2 mg/d + MPA 10 mg/d)	Transdermal	Placebo	N	3	HAMD
Soares et al. (23)	USA	50	0.0	Peri-M women diagnosed with depressive disorders	49.7 ± 3.9	Transdermal 17β-estradiol 100 ug	Transdermal	Placebo	26.00	12	MADRS
Casini et al. (41)	Italy	78	0.0	Post-M	49.0 ± 4.7	Isoflavones 60 mg/d	Oral	Placebo	N	24	HAMD, STAI
Berlanga et al. (50)	Mexico	31	0.0	Post-M women diagnosed with depressive disorders	53.4 ± 2.8	Tibolone 2.5 mg/d	Oral	Placebo	51.60	8	HAMD
Kulkarni et al. (44)	Australia	44	0.0	Peri-M women diagnosed with depressive disorders	52.1 ± 5.7	Tibolone 2.5 mg/d	Oral	Placebo	N	12	MADRS
Gleason et al. (79)	USA	619	6.1	Peri-M	52.6 ± 2.6	EPT (CEE 0.45 mg/d + m-P 200 mg/d) EPT (E2 50 μg/d + m-P 200 mg/d)	Oral Transdermal	Placebo	40.00	208	BDI
Baksu et al. (43)	Turkey	65	0.0	Surgical menopause	N	Tibolon 2.5 mg/d, Transdermal estradiol 557 mg/d	Oral Transdermal	Placebo	N	24	HAMD, HAMA
Cintron et al. (71)	USA	653	10.2	Peri-M	52.7 ± 2.6	CEE 0.45 mg/d, Transdermal 17β-estradiol 50 ug/d	Oral Transdermal	Placebo	N	192	PSQI
Kagan et al. (72)	USA	572	9.7	Post-M	54.5 ± 4.3	EPT (E2 0.5 mg/d + progesterone 50 mg/d)	Oral	Placebo	N	48	MOS sleep scale
Tansupswatdikul et al. (34)	Thailand	36	10.0	Post-M	54.4 ± 3.9	Transdermal E2 50 μg/d	Transdermal	Placebo	N	8	ISI
Gordon et al. (7)	Canada	132	23.3	Peri-M	51.0 ± 3.0	Transdermal 17β-estradiol 100 ug/d	Transdermal	Placebo	41.80	48	CES-D
Aghamiri et al. (38)	Iran	120	0.0	Peri-M	38.7 ± 6.7	Phytoestrogen Hop (Humulus lupulus L.) 500 mg/d	Oral	Placebo	30.80	12	GCS
Shamshad et al. (78)	India	70	20.5	Post-M	53.7 ± 4.5	Phytoestrogen Fenugreek Husk 1 g/d	Oral	Placebo	58.60	12	GCS
Park et al. (35)	Korea	36	12.2	Peri-M	51.9 ± 6.0	Phytoestrogen Schisandra chinensis 784 mg	Oral	Placebo	N	12	KI, MRS
Saletu et al. (81)	Austria	53	23.2	Post-M	51.2 ± 3.3	Transdermal E2 50 μg/d	Transdermal	Placebo	N	12	HAMD
Kotsopoulos et al. (36)	Australia	94	20.2	Post-M	59.7 ± 1.4	Phytoestrogen soy supplements containing 118 mg/d	Oral	Placebo	N	12	KI
Evans et al. (76)	Australia	72	10.0	Post-M	61.5 ± 1.2	Phytoestrogen Resveratrol 150 mg/d	Oral	Placebo	42.10	14	CES-D, POMS
Sousa et al. (64)	Brazil	76	9.5	Post-M	53.3 ± 3.6	Phytoestrogen soy isoflavones extract 120 mg/d	Oral	Placebo	38.60	16	CES-D
Wiklund et al. (22)	Sweden	223	6.7	Post-M	52.5 ± 4.8	Transdermal E2 50 μg/d	Transdermal	Placebo	N	12	WHQ
Atteritano et al. (77)	Italy	229	14.4	Post-M	52.7 ± 2.0	Phytoestrogen isoflavone genistein 54 mg/d	Oral	Placebo	N	104	SF36, ZSRD
Lipovac et al. (65)	Austria	109	3.5	Post-M	53.5 ± 7.1	Phytoestrogen red clover isoflavones 80 mg/d	Oral	Placebo	N	26	HADS

AIS, Athens Insomnia Scale; BAI, Beck Anxiety Inventory; BDI, Beck Depression Inventory; BZA, Bazedoxifene; CCEI, Crown-Crisp experiential index; CE, conjugated estrogens; CEE, conjugated equine estrogens; CES-D, the center for epidemiological studies Depression Scale; E, estrogen; EPT, estrogen plus progesterone therapy; EV, estradiol valerate; GAD-7, Generalized Anxiety Disorder Questionnaire-7; GCS, Greene Climacteric Scale; GHQ, General Health Questionnaire; HADS, Hospital Anxiety and Depression Scale; HAMA, Hamilton Anxiety Scale; HAMD, Hamilton Depression Scale; IHAD, Iranian version of Hospital Anxiety and Depression Questionnaire; ISI, Insomnia Severity Index; KI, Kupperman Index; MADRS, Montgomery-Asberg Depression Rating Scale; MENQOL, Menopause-Specific Quality of Life Questionnaire; MMSE, Mini_x005f_x005f_x005f_x0002_Mental State Examination; MOS, medical outcomes study; m-P, micronized progesterone; MPA, medroxyprogesterone acetate; MRS, Menopause Rating Scale; NA, not applicable; NETA, norethisterone acetate; NHP, nottingham health profile; PANAS, the Positive and Negative Affect Scale; Peri-M, peri-menopausal; PHQ-9, Patient Health Questionnaire-9; POMS, the profile of mood states; Post-M, post-menopausal; PSQI, Pittsburgh Sleep Quality Index; PSS, Perceived Stress Scale; QOL, quality of life; RAND36, RAND 36-item health survey; SCL-90, symptom checklist 90; SDS, sleep disturbance score; SF36, short form 36 health survey; SRSD, Self Rating Scale of distress; SRSS, the Self Rating Scale of sleep; SSQ, The Stanford Sleepiness Questionnaire; STAI, State–Trait Anxiety Inventory; TST, total sleep time (min); UK, the United Kingdom; USA, the United States of America; VAS, Visual Analog Scale; WHIIRS, Women's Health Initiative Insomnia Rating Scale; WHOQOL, world health organization quality of life; WHQ, the Women's Health Questionnaire; ZSRD, Zung Self-rating Depression Scale.

**Table 2:** Characteristics of included studies.

Characteristics	Hormone therapy		Phytoestrogens		Total	
	Trials	Sample size	Trials	Sample size	Trials	Sample size
Overall	51	41,821	16	1,191	67	43,012
Age (mean ± SD)	54.2 ± 3.8		51.6 ± 3.1		52.9 ± 3.6	
Drop out (%)	7.6		11.2		9.5	
Regions						
Asia	8	808	6	363	14	1,171
Europe	17	5,015	4	459	21	5,474
Australia	3	210	2	166	5	376
North America	20	32,577	1	46	21	32,623
South America	2	78	3	157	5	235
Multinational	1	3,133	NA	NA	1	3,133
Participants						
Perimenopause	11	2,006	6	330	17	2,336
Post-menopause	34	39,249	8	766	42	40,015
Peri- & post- menopause	3	356	2	95	5	451
Surgical menopause	3	210	NA	NA	3	210
Duration						
≤ 12 weeks	28	2,265	9	546	37	2,811
13–52 weeks	17	21,943	6	416	23	22,359
≥ 53 weeks	6	17,613	1	229	7	17,842
Interventions						
Estrogen	26	13,659	NA	NA	26	13,659
Progesterone	2	41	NA	NA	2	41
EPT	17	24,725	NA	NA	17	24,725
Tibolone	6	3,396	NA	NA	6	3,396
Soybean	NA	NA	3	213	3	213
Isoflavone	NA	NA	5	512	5	512
Others	NA	NA	8	466	8	466
Route of administration						
Oral	39	40,686	16	1,191	39	41,877
Transdermal	10	705	NA	NA	10	705
Intranasal	1	232	NA	NA	1	232
Vaginal	1	198	NA	NA	1	198

Among the 67 included studies in the analysis, hormone therapy was implemented as an intervention in 51, whereas phytoestrogens were employed in 16.

Several studies did not provide data on dropouts.

EPT, estrogen plus progesterone therapy; NA, not available; Peri- & post-M, Peri- & post-menopause.

### 3.3 Effects of interventions on mood

**Figure 2:**
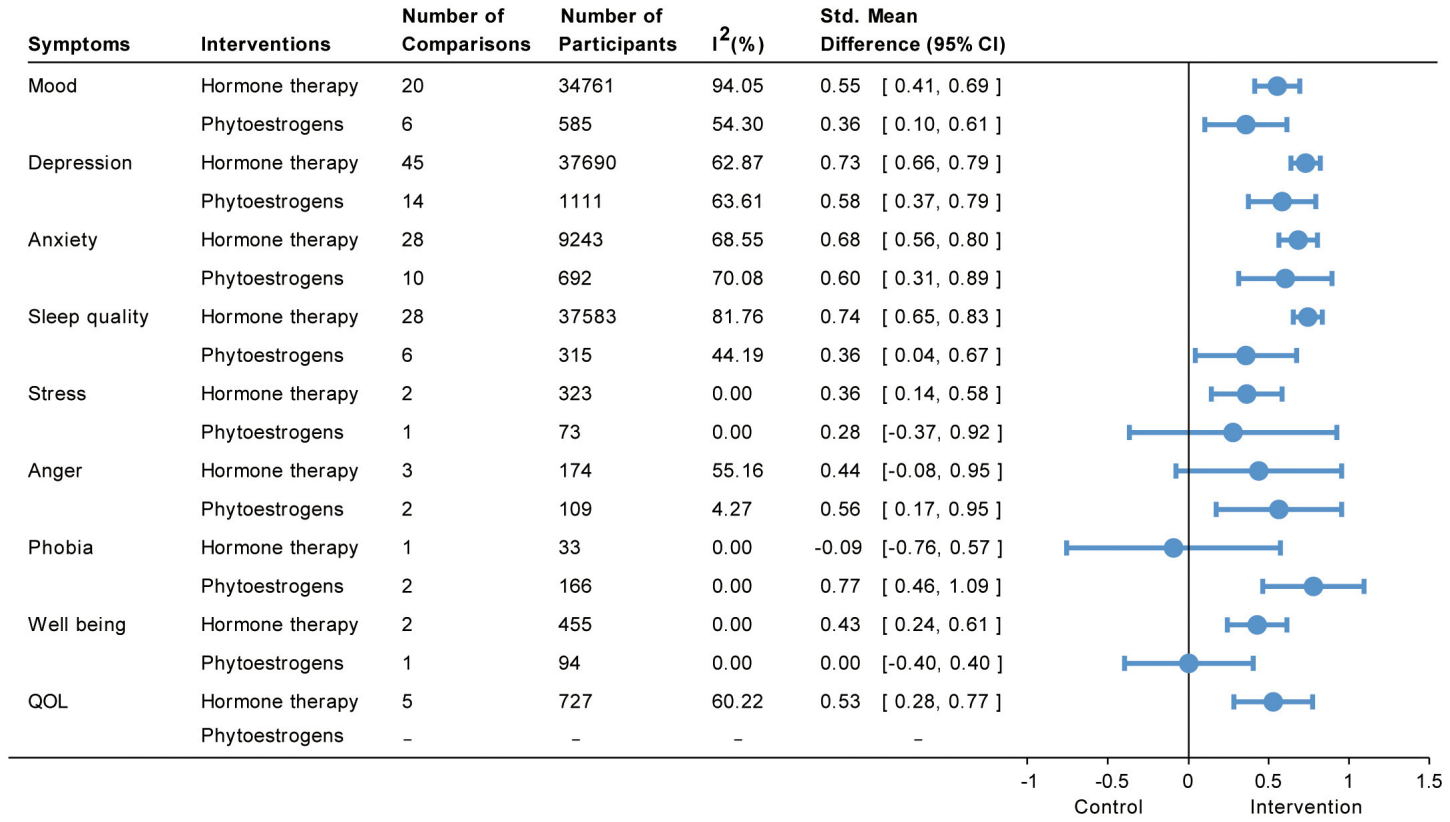
Efficacy of hormone therapy and phytoestrogens on the psychological health of menopausal women. Hormone therapy and phytoestrogens demonstrated small to moderate efficacy in mitigating psychological symptoms relative to placebo among menopausal women, with varying effects on different psychological symptoms. CI, confidence interval; QoL, quality of life.

**Figure 3:**
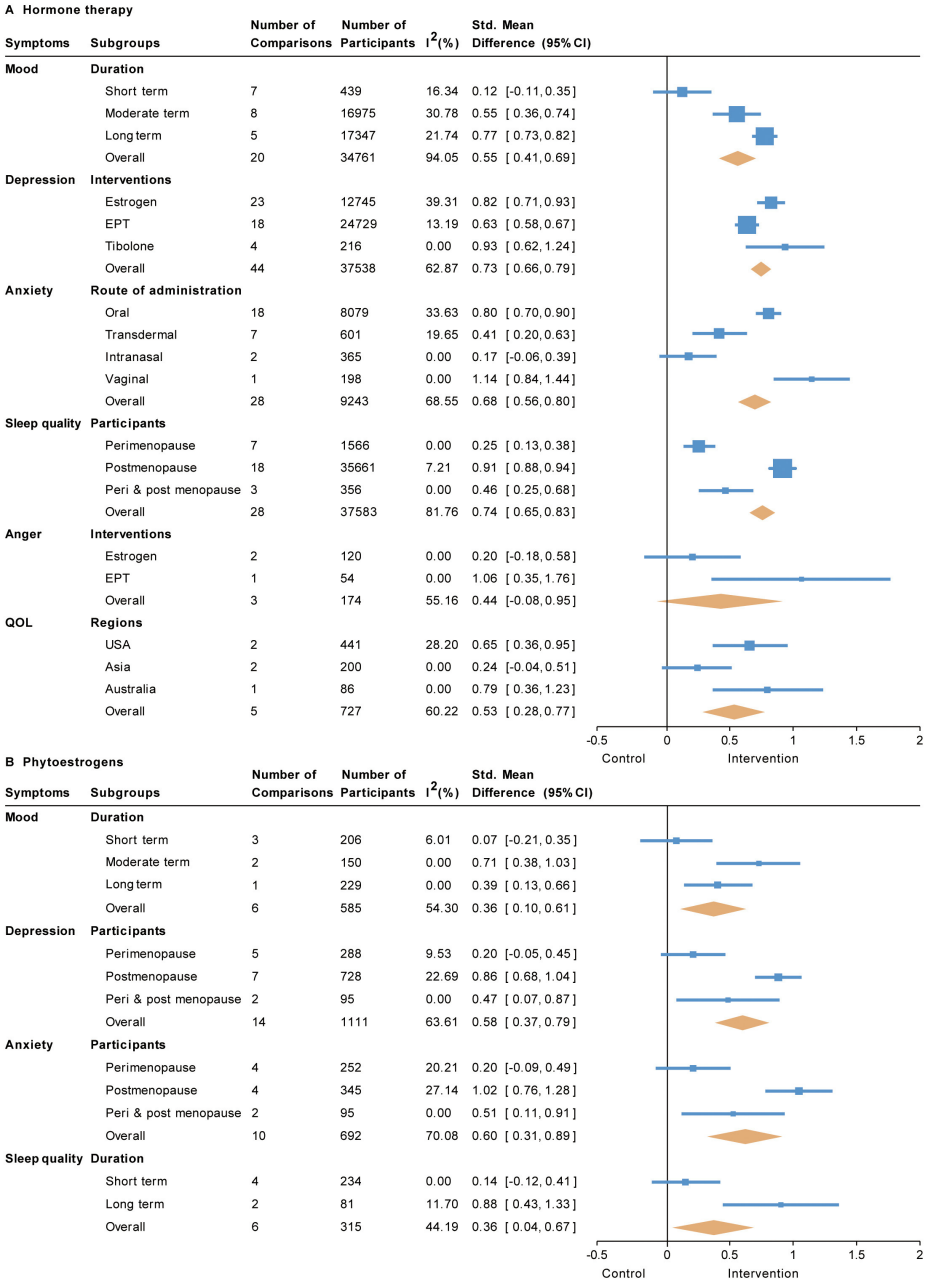
Subgroup analysis on the psychological health of menopausal women. **(A)** Comparison of women undergoing hormone therapy vs. those in the control group. **(B)** Comparison of women undergoing phytoestrogens vs. those in the control group. The overall result is indicated in bold. CI, confidence interval; EPT, estrogen plus progesterone therapy; QoL, quality of life.

In terms of mood, the hormone therapy groups exhibited superior performance compared to the control groups (SMD: 0.55, 95% CI: 0.41–0.69; *I*^2^: 94.05; Figure 2). The *I*^2^ value was large, and the heterogeneity of result decreased following the subgroup analysis. Subgroup analysis revealed that compared to the non-treated controls, hormone therapy exhibited a moderate effect in the long term (SMD: 0.77, 95% CI: 0.73–0.82; *I*^2^: 21.74), but no effect in the short term (SMD: 0.12; Figure 3A). The effect size observed between the phytoestrogen and control groups was 0.36 (95% CI: 0.1–0.61; *I*^2^: 54.3; Figure 3B). The effect size was most pronounced in the intermediate term (SMD: 0.71, 95% CI: 0.38–1.03), exhibiting a degree of fluctuation over time. And the results of the meta-regression analyses were consistent with those of the subgroup analyses. In meta-regression analyses, the heterogeneity of results were mostly related to duration of therapy (Supplementary eTables 5, 7, 8). The results of the subgroup analysis are shown in Supplementary eFigures 1–17. The bubble plots of the meta-regression are shown in Supplementary eFigures 18–26.

### 3.4 Effects of interventions on depression

In the 44 comparisons of depression between hormone therapy and control groups, the pooled effect sizes and heterogeneity were moderate (SMD: 0.73, 95% CI: 0.66–0.79; *I*^2^: 62.87). Following the subgroup analysis, the heterogeneity of results decreased. Subgroup analysis indicated that studies with estrogen (SMD: 0.82, 95% CI 0.71–0.93; *I*^2^: 39.31) and Tibolone (SMD: 0.93, 95% CI 0.62–1.24; *I*^2^: 0) demonstrated greater effect sizes than those involving estrogen plus progesterone therapy (EPT; SMD: 0.63, 95% CI 0.58–0.67; *I*^2^: 13.19). And based on the meta-regression analysis, the main sources of heterogeneity were related to the interventions, while Tibolone showed significant therapeutic efficacy (β: −0.27, 95% CI −0.35 to −0.19); *p* < 0.001). The effect size of depression exhibited moderate levels after the intervention of phytoestrogens (SMD: 0.58, 95% CI: 0.37–0.79; *I*^2^: 63.61). The efficacy of phytoestrogens in post-menopausal women was found to be significantly more impactful than that in the perimenopausal stage.

### 3.5 Effects of interventions on anxiety and stress

Based on 18 comparisons between hormone therapy and control groups, the effect size for anxiety was 0.68 (95% CI: 0.56–0.8; *I*^2^: 68.55). The results of the subgroup analysis and the meta-regression analysis indicated a statistically significant distinction among the subgroups classified based on the route of administration. The effect size of the oral format (SMD: 0.8, 95% CI 0.7–0.9; *I*^2^: 33.63) was found to be considerably greater than that of the transdermal (SMD: 0.41, 95% CI 0.2–0.63; *I*^2^: 19.65) and intranasal (SMD: 0.17, 95% CI −0.06 to 0.39; *I*^2^: 0.00) methods. One study using the vaginal format demonstrated a significant effect size. The pooled effect size and heterogeneity for the 10 comparisons of anxiety between phytoestrogens and placebo exhibited moderate (SMD: 0.6, 95% CI: 0.31–0.89; *I*^2^: 70.08). The effect size of post-menopause (SMD: 1.02, 95% CI 0.76–1.28; *I*^2^: 27.14) was significantly higher than that of perimenopause (SMD: 0.2, 95% CI −0.09 to 0.49; *I*^2^: 20.21). The effect sizes of both hormone therapy and phytoestrogens were small under stress (SMD: 0.36 and 0.28), with low heterogeneity.

### 3.6 Effects of interventions on sleep quality

The effect size of hormone therapy (SMD: 0.74, 95% CI: 0.65–0.83; *I*^2^: 81.76) exhibited a greater magnitude in improving sleep quality compared to phytoestrogens (SMD: 0.36, 95% CI: 0.04–0.67; *I*^2^: 44.19). After subgroup analysis, there was a decrease in heterogeneity. A significant distinction between post-menopause (SMD: 0.91, 95% CI 0.88–0.94; *I*^2^: 7.21) and perimenopause (SMD: 0.25, 95% CI 0.13–0.38; *I*^2^: 0.00) was observed following hormone therapy. In addition, the effect size demonstrated its greatest magnitude during the intermediate term (SMD: 0.88, 95% CI 0.43–1.33; *I*^2^: 11.7) following intervention with phytoestrogens, displaying variability over time. And the meta-regression analysis also revealed that variations in therapy duration accounted for sources of heterogeneity.

### 3.7 Rate of adverse event reporting

**Figure 4:**
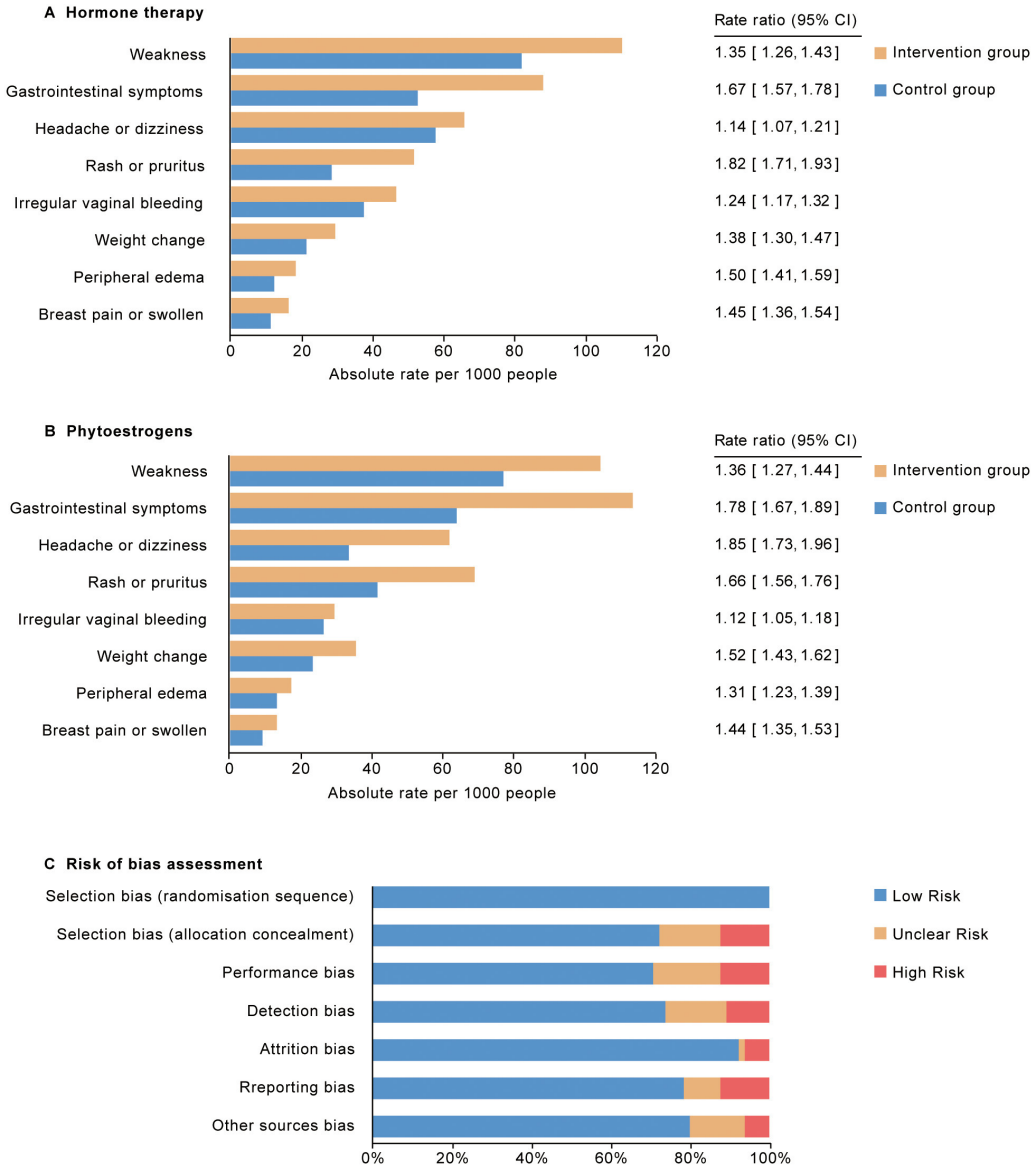
Rate of adverse event reporting and risk of bias assessment. **(A)** Rate of adverse event reporting of hormone therapy. **(B)** Rate of adverse event reporting of phytoestrogens. **(C)** Risk of bias assessment.

After the intervention, there were several adverse reactions with a high reporting rate. Rashes/pruritus (1.82 [95% CI, 1.71 to 1.93]) were the most commonly reported adverse events in the hormone therapy groups, followed by gastrointestinal symptoms, peripheral edema, and breast pain/swelling (Figures 4A, B). In the phytoestrogens groups, adverse events were largely related to headache/dizziness (1.85 [95% CI, 1.73 to 1.96]), followed by gastrointestinal symptoms, rash or pruritus, and weight change.

### 3.8 Quality assessments

The ultimate outcomes of the risk-of-bias assessments are depicted in Figure 4C and Supplementary eTable 4. Random sequences were adequately generated in all of the included studies. Eight studies (12.31%) did not implement blinding of participants and personnel, whereas 11 studies (16.92%) did not provide any information regarding this aspect. Blinding of outcome assessment was reported in 48 studies (73.85%), whereas it was not reported in seven studies (10.77%). In general, the risk of bias was not serious, and there was good agreement between the raters (kappa = 0.87). Forest plots and Galbraith plots are provided in Supplementary eFigures 27–30.

### 3.9 Publication bias

Egger's test revealed a modest level of asymmetry (*P* < 0.05) in the funnel plot of anxiety and sleep quality. However, the application of Duval and Tweedie's trim and fill procedure indicated the absence of publication bias concerning these symptoms. In addition, Egger's test revealed no evidence of publication bias in other symptoms. Moreover, there was no evidence of inconsistency in the present meta-analysis, whether it was local inconsistency, assessed through the loop-specific approach and the node-splitting method, or global inconsistency, determined through the design-by-treatment method.

### 3.10 Trial sequential analysis

For hormone therapy, the cumulative Z-curves crossed the trial sequential monitoring boundaries for mood, anxiety, and sleep quality, although the RIS was not achieved. These findings indicate that the positive effects of hormone therapy on these three outcomes remain statistically significant even after correction for multiple testing, with an extremely low risk of false-positive conclusions. Similarly, for phytoestrogens, the cumulative Z-curves exceeded the monitoring boundaries for mood, depression, and anxiety, confirming the robustness of these positive results despite incomplete sample size accumulation. The results are presented in Supplementary eFigures 31–47.

In contrast, although meta-analysis showed statistically significant effects of hormone therapy on depression, stress, and quality of life, and of phytoestrogens on sleep quality and fear, none of these outcomes passed TSA correction. This suggests that these positive findings may be influenced by multiple testing bias, and their true therapeutic effects require further validation in larger, high-quality clinical trials. No significant improvement was observed for anger in the hormone therapy group, or for fear and wellbeing in the phytoestrogen group. Furthermore, the sample sizes for most of these non-significant outcomes were far below the pre-specified RIS, precluding definitive conclusions regarding their efficacy.

## 4 Discussion

To the best of our knowledge, the present study is the most comprehensive meta-analysis conducted to investigate the effectiveness and tolerability of hormone therapy and phytoestrogens in managing the psychological health of menopausal women.

### 4.1 The primary findings of this study

Based on 67 RCTs recruiting a total of 43,012 patients, we observed different effects on the psychological symptoms of menopausal women. The heterogeneity of results decreased following the subgroup analysis and meta-regression. We also found that hormone therapy and phytoestrogens had a positive effect on depression in menopausal women, but no significant effect in the short term. Additionally, the tolerability, as indicated by the rate of adverse event reporting and participant discontinuation, was comparatively lower in the treatment group than in the placebo/control group.

Several previous meta-analyses assessed the outcome of only one symptom, and potentially important between-study differences remained unexplored, including participants and therapeutic regimens. For example, some meta-analyses have only assessed the effects of hormone therapy and phytoestrogens on depression, anxiety, or sleep (10, 85, 86). And several authors have only explored the effects of estrogen on psychological health of menopausal women, without evaluating the impact of other hormonal therapies (87, 88). In addition, other meta-analyses included only peri- or post-menopausal women and have not investigated the impact of these two populations (89, 90).

### 4.2 Psychological symptoms are affected by hormone therapy

We found that hormone therapy has moderate effect on depression, anxiety, and sleep quality, and slight effect on anger. In hormone therapy, estrogen plays a key role in improving psychological issues. Estrogen, a neuroactive steroid, affects psychological symptoms in multiple ways, including neurotransmitter deficiency, neuroplasticity, cellular energetics, inflammation, and network dysregulation (91). Estrogen plays a regulatory role in the synthesis, metabolism, and receptor trafficking of the classical neurotransmitters serotonin, dopamine, and norepinephrine (92). For example, lower estrogen levels may contribute to the onset of depression through decreased 5-HT_2A_ density and serotonin activity (93). Estrogen receptors have been identified within the human suprachiasmatic nucleus, suggesting that variations in estrogen levels could influence circadian rhythms (94). A previous study showed that decreased estradiol can induce sleep maintenance difficulties, whereas elevated estrogen demonstrates a protective effect against insomnia (95). In addition, previous neuroimaging investigations conducted on both human and animal subjects have provided further evidence to support the correlation between estrogen fluctuations and brain structures and functions (96). However, the etiology and pathophysiological mechanisms underlying the impact of estrogen on psychological symptoms remain incompletely understood, thereby posing a significant obstacle to providing appropriate therapy. It is imperative to conduct pre-clinical studies using animal models to investigate the neurobiological pathways underlying the direct and indirect effects on psychological symptoms (89).

### 4.3 Influenced by the different hormone therapies

epression, estrogen-only therapy was more effective compared with EPT, while the opposite was true for anger. Indeed, the inclusion of progestin in combined hormone therapy results in a controversial effect profile on psychological symptoms. Animal experimental study has shown progesterone reduced depressive behavior of young ovariectomized, aged progestin receptor knockout, and aged wild type mice in the tail suspension test (97). A randomized controlled trial demonstrated that combined treatment with transdermal estradiol and micronized progesterone effectively alleviates depressive symptoms during the menopause transition (7). In contrast, another study showed that progestins, either concurrently or consecutively with estrogen, had the potential to induce mood destabilization (79). We found Tibolone showed an enhancement effect on depression (SMD = 0.93) ompared with other hormone therapies. Tibolone is a synthetic steroid with tissue-selective estrogenic, androgenic and progestogenic properties. The 3α-hydroxy and 3-hydroxy metabolites of Tibolone are responsible for the estrogenic effects of Tibolone in the brain by activating estrogen receptors (98). Kulkarni et al. (44) found that participants in the Tibolone group demonstrated a significant improvement in depression scores compared to the placebo group, without any significant side effects. However, given the limited number of trials, conclusions regarding tibolone should be interpreted with caution, taking into consideration the small sample sizes, regional differences, and variations in tibolone dosages. As healthcare professionals, it is imperative to consider that all hormone therapies possess distinct characteristics and exert diverse biological effects.

### 4.4 Psychological symptoms are affected by phytoestrogens

Our study provided evidence that phytoestrogens has moderate effect on depression and anxiety, and slight effect on sleep quality. Phytoestrogens, molecules derived from plants, have garnered significant attention because of their estrogenic properties (99). The potential antidepressant effects of phytoestrogens may be attributed to their ability to enhance estrogen levels, consequently improving serotonin levels and the activity of 5-HT_2A_ (100). Moreover, previous studies have indicated that the presence of flavonoids derived from phytoestrogens can effectively elevate the levels of brain serotonin (101). Nevertheless, the efficacy of phytoestrogens remained uncertain because of methodological deficiencies and a limited sample size. In addition, the diverse dietary practices among women in different countries, such as the prevalent consumption of soy products in Japan and Korea, may influence the outcomes of phytoestrogens (102). Considering that phytoestrogens are not standard therapeutic option for perimenopausal mood disorders, a balanced consideration of their efficacy and potential adverse effects is warranted in clinical practice. Recommendations for future trials include conducting comparative studies to evaluate the impact of phytoestrogens compared to other treatments, such as SSRIs.

### 4.5 Impact of perimenopausal and post-menopausal stages

Our results showed that compared with perimenopausal women, post-menopausal women exhibited a greater beneficial effect of hormone therapy on sleep quality and of phytoestrogens on depression and anxiety. Therefore, although several studies have included both perimenopausal and post-menopausal women, they should not be mixed. Some researchers have posited that the menopausal transition may represent a period of heightened susceptibility for women, as they may be more sensitive to hormonal fluctuations akin to those experienced during pre-menstrual phases (103). It has also been revealed that psychological symptoms exhibited a correlation with estradiol fluctuations, in that the faster the change, the more severe the psychological symptoms, indicating that the dynamics of altering hormone levels hold greater significance than the absolute levels themselves (104). The fluctuation of estrogen levels during the menopausal transition was also found to be more prominent than that during the post-menopausal stage, even when exogenous estrogen was administered (105). This evidence may explain why psychiatric symptoms differ in response to hormonal therapy in women before and after menopause and during the menopausal transition.

### 4.6 Similarities and differences between hormone therapy and phytoestrogens

This study found that hormone therapy and phytoestrogens yield similar results in improving mood disorders in perimenopausal women. We found that those in post-menopause exhibited a significant effect on improving depression, anxiety, and sleep quality, regardless of whether they were undergoing hormone therapy or using phytoestrogens. And the therapeutic efficacy on psychological symptoms was more pronounced with moderate- and long-term interventions compared to short-term. But the therapeutic efficacy of hormone therapy is superior to that of phytoestrogens in improving depression, anxiety, and sleep quality. Phytoestrogens primarily exert their effects by mimicking the action of estrogen and regulating estrogen receptors, but their effects are weaker compared to hormone therapy. The advantage of phytoestrogens lies in their fewer side effects, particularly the lower risks associated with breast cancer, cardiovascular diseases, and thrombosis, although this remains a subject of debate (106). While some small-scale studies suggest that phytoestrogens can alleviate anxiety and depressive symptoms, these studies have small sample sizes and methodological limitations (24). Phytoestrogens can somewhat reduce anxiety symptoms in perimenopausal women, but their effects are far less pronounced than hormone therapy (76). Overall, hormone therapy demonstrates a significant advantage in improving mood disorders in perimenopausal women, especially for treating depression and anxiety. Although phytoestrogens have some potential in improving psychological symptoms, their effects tend to be milder. Phytoestrogens may be an effective alternative, but for patients with more severe symptoms, hormone therapy remains the more effective treatment.

### 4.7 Adverse events of hormone therapy and phytoestrogens

When considering the clinical use of hormone therapy and phytoestrogens, clinicians must also pay attention to their adverse events. We found that rash/itching, gastrointestinal symptoms, and peripheral edema are common adverse events of hormone therapy. Headache/dizziness and gastrointestinal symptoms are the adverse events associated with phytoestrogens. These results indicate the differences in the adverse events between hormone therapy and phytoestrogens. Although some studies report that the side effects of phytoestrogens are milder than those of hormone therapy, this is not observed in the study. The reason might be that this study includes menopausal women, who are more sensitive to drug treatments (107, 108). When selecting a treatment approach, individual patient differences and their tolerance to adverse events must be taken into consideration.

### 4.8 Trial sequential analysis

Our trial sequential analysis results offer valuable and specific guidance for the design of future clinical trials. For outcomes where the cumulative Z-curve crossed the trial sequential monitoring boundaries, further large-scale trials are unlikely to alter the fundamental conclusions. This applies to mood, anxiety and sleep quality for hormone therapy, and to mood, depression and anxiety for phytoestrogens. Future research in these areas should instead focus on identifying optimal treatment regimens, patient subgroups that derive the greatest benefit and long-term safety profiles (109).

In contrast, some outcomes showed statistical significance in conventional meta-analysis but failed to pass trial sequential analysis correction. These include depression, stress, and quality of life for hormone therapy, as well as sleep quality and fear for phytoestrogens. Additional high-quality and adequately powered studies are required to confirm their true therapeutic effects. For outcomes with no significant improvement and insufficient sample size, future trials should be designed with larger sample sizes and more standardized outcome measures to generate definitive evidence (110). This category includes anger for hormone therapy, as well as fear and wellbeing for phytoestrogens.

### 4.9 Strengths and limitations

This study offers several strengths. First, the inclusion of only RCTs ensured a high or moderate quality of evidence, and the heterogeneity could be explained through subgroup analyses. Second, the rate of adverse event reporting for hormone therapy and phytoestrogens was estimated. Finally, we analyzed the impacts of various drug combinations, duration of intervention, and modes of drug administration on therapeutic effectiveness, thereby offering a theoretical basis for medical decision-making.

Despite these advantages, our study also has limitations that warrant discussion. First, the evidence obtained from the present study may be obscured because of the diverse characteristics of the participants, such as the presence of comorbid diseases and varying levels of baseline depression severity. Although we attempted to mitigate heterogeneity through subgroup analysis and meta-regression, certain effects may persist. Second, the adverse events of intervention are only explored crudely because the included studies each define and evaluate adverse reactions differently. Third, given that the included trials included women with other symptoms (e.g., vasomotor symptoms), the observed enhancements in psychological symptoms were attributed to the mitigation of potential worsening factors such as hot flashes and night sweats. Fourth, due to the extensive inclusiveness of this study encompassing diverse participant characteristics, intervention protocols, treatment durations, and outcome measures, the pooled results cannot be directly applied to real-world clinical practice. Instead, its primary value is to provide a systematic overview of current evidence and guide the design of more targeted, rigorous future clinical trials on this topic.

## 5 Conclusions

Hormone therapy and phytoestrogens exhibited effectiveness in alleviating psychological symptoms in menopausal women, with diverse impacts on various psychological symptoms. Moreover, the effects of the intervention were impacted by the specific hormone regimen used. Targeted health education should be delivered to relevant populations to improve health awareness and treatment adherence. Our results provided reliable evidence to base future treatment decisions, clinical guidelines, and clinical trial designs.

## Data Availability

The original contributions presented in the study are included in the article/Supplementary material, further inquiries can be directed to the corresponding authors.
