# Supplementary material for: Efficacy of hormone therapy and phytoestrogens on the psychological symptoms of menopausal women: a systematic review, meta-analysis, and trial sequential analysis
Source: Front Med (Lausanne). 2026 Jul 10;13:1855845. doi: 10.3389/fmed.2026.1855845 (PMC13395877; doi:10.3389/fmed.2026.1855845)
Supplement: Supplementary file 1 [file Supplementary_file_1.docx]

**Supplementary material**

[eTable 1. The themes and the related terms 1](#_Toc23740)

[eTable 2. Search strategies for some databases searched 2](#_Toc16529)

[eTable 3. Table of subgroup classification 5](#_Toc20802)

[eTable 4. Quality assessment of included studies 10](#_Toc29647)

[eTable 5. Classification effect and source of heterogeneity 13](#_Toc12989)

[eTable 6. Preferred reporting items for systematic reviews and meta-analyses 14](#_Toc21732)

[eFigure 1-17. Forest plots of meta-analysis 19](#_Toc6540)

[eTable 7. Meta-regression summary of hormone therapy 29](#_Toc12910)

[eTable 8. Meta-regression summary of phytoestrogens 31](#_Toc15015)

[eFigure 18-26. Bubble plots of hormone therapy and phytoestrogens 32](#_Toc21264)

[eFigure 27-28. Forest plots of hormone therapy and phytoestrogens 35](#_Toc14728)

[eFigure 29-30. Galbraith plots of hormone therapy and phytoestrogens 37](#_Toc28212)

[eFigure 31-47. Trial sequential analysis of hormone therapy and phytoestrogens 39](#_Toc29284)

### eTable 1. The themes and the related terms

| **Themes** | **Related terms** | |
| --- | --- | --- |
| Menopausal | Change of Life  Climacteric  Climacterics  Perimenopausal  Perimenopause  Postmenopausal | Post-Menopausal  Postmenopause  Premenopausal  Pre-menopausal  Premenopause  Pre-Menopause |
| Hormone therapy | Anordrin  Chlorotrianisene  Conjugated Estrogens  Cyproterone  Desogestrel  Diethylstilbestrol  Drospirenone  Estradiol  Estradiol Benzoate  Estradiol Cypionate  Estradiol Valerate  Estrogen therapy  Ethinylestradiol  Gestrinone  Hexestrol | Hormone Replacement  Hormone therapy  Levonorgestrel  Medroxyprogesterone  Megestrol  Nilestriol  Nomegestrol  Nonoxynol  Norethindrone  Norgestrel  Phytoestrogen  Promegestone  Promestriene  Promestriene  Tibolone |
| Phytoestrogens | Daidzein  Flax  Flaxseed  Genistein  Isoflavon  Isoflavones  Phytoestrogen  Phyto-estrogen  Phytoestrogens | Phytooestrogen  Phyto-oestrogen  Red clover  Soy  Soy Foods  Soybean  Soybeans  Tofu  Trifolium |
| Psychological symptoms | Anxiety  Anxiety  Anxious  Depressed  Depression  Depression  Depressions, Emotional  Depressive Symptom  Depressive Symptoms  Emotion  Emotional Depression | Emotional Depressions  Hyposomnia  Insomnia  Mental  Nervousness  Psychological  Sleep  Sleeplessness  Stress  Stressful  Symptoms, Depressive |

### eTable 2. Search strategies for some databases searched

| **Pubmed** |
| --- |
| (((((((((((Perimenopausal[Title/Abstract]) OR (Postmenopausal[Title/Abstract])) OR (Post-Menopausal[Title/Abstract])) OR (perimenopause[Title/Abstract])) OR (Pre-Menopause[Title/Abstract])) OR (Pre-menopausal[Title/Abstract])) OR (Premenopausal[Title/Abstract])) OR (Premenopause[Title/Abstract])) OR (Postmenopause[Title/Abstract])) OR (Climacteric[Title/Abstract])) OR (Climacterics[Title/Abstract])) OR (Change of Life[Title/Abstract])) AND (((((((((((((((((((((((((((((((((((((((((((((((((Hormone Replacement[Title/Abstract]) OR (Estrogen therapy[Title/Abstract])) OR (Hormone therapy[Title/Abstract])) OR (Estradiol[Title/Abstract])) OR (Phytoestrogen[Title/Abstract])) OR (Estradiol Valerate[Title/Abstract])) OR (Estradiol Benzoate[Title/Abstract])) OR (Estradiol Cypionate[Title/Abstract])) OR (Chlorotrianisene[Title/Abstract])) OR (Promestriene[Title/Abstract])) OR (Hexestrol[Title/Abstract])) OR (Ethinylestradiol[Title/Abstract])) OR (Nilestriol[Title/Abstract])) OR (Conjugated Estrogens[Title/Abstract])) OR (Diethylstilbestrol[Title/Abstract])) OR (Megestrol[Title/Abstract])) OR (Norgestrel[Title/Abstract])) OR (Norethindrone[Title/Abstract])) OR (Levonorgestrel[Title/Abstract])) OR (Desogestrel[Title/Abstract])) OR (Gestrinone[Title/Abstract])) OR (Anordrin[Title/Abstract])) OR (Nonoxynol[Title/Abstract])) OR (Tibolone[Title/Abstract])) OR (Medroxyprogesterone[Title/Abstract])) OR (Promestriene[Title/Abstract])) OR (Cyproterone[Title/Abstract])) OR (Promegestone[Title/Abstract])) OR (Nomegestrol[Title/Abstract])) OR (Drospirenone[Title/Abstract])) OR (Phytoestrogen[Title/Abstract])) OR (Phytoestrogens[Title/Abstract])) OR (Soybeans[Title/Abstract])) OR (Soybean[Title/Abstract])) OR (Soy Foods[Title/Abstract])) OR (Trifolium[Title/Abstract])) OR (Isoflavones[Title/Abstract])) OR (Phytooestrogen[Title/Abstract])) OR (Phyto-estrogen[Title/Abstract])) OR (Phyto-oestrogen[Title/Abstract])) OR (Soy[Title/Abstract])) OR (Red clover[Title/Abstract])) OR (Isoflavon[Title/Abstract])) OR (Daidzein[Title/Abstract])) OR (Genistein[Title/Abstract])) OR (Flaxseed[Title/Abstract])) OR (Tofu[Title/Abstract])) OR (Flax[Title/Abstract])) OR (Flaxseed[Title/Abstract])) OR (Linum usitatissimum[Title/Abstract])) AND ((((((((((((((((((((((Mental[Title/Abstract]) OR (Psychological[Title/Abstract])) OR (Emotion[Title/Abstract])) OR (Depression[Title/Abstract])) OR (Emotional Depression[Title/Abstract])) OR (Symptoms, Depressive[Title/Abstract])) OR (Depressive Symptom[Title/Abstract])) OR (Depressive Symptoms[Title/Abstract])) OR (Emotional Depressions[Title/Abstract])) OR (Depressions, Emotional[Title/Abstract])) OR (Depression[Title/Abstract])) OR (Depressed[Title/Abstract])) OR (Anxiety[Title/Abstract])) OR (Anxious[Title/Abstract])) OR (Hypervigilance[Title/Abstract])) OR (Nervousness[Title/Abstract])) OR (Anxiety[Title/Abstract])) OR (Stress[Title/Abstract])) OR (Stressful[Title/Abstract])) OR (Sleep[Title/Abstract])) OR (Insomnia[Title/Abstract])) OR (Sleeplessness[Title/Abstract])) OR (Hyposomnia[Title/Abstract])) |
| **PsycINFO** |
| 'Perimenopausal':ad,ti OR 'Postmenopausal':ad,ti OR 'Post-Menopausal':ad,ti OR 'perimenopause':ad,ti OR 'Pre-Menopause':ad,ti OR 'Pre-menopausal':ad,ti OR 'Premenopausal':ad,ti OR 'Premenopause':ad,ti OR 'Postmenopause':ad,ti OR 'Climacteric':ad,ti OR 'Climacterics':ad,ti OR 'Change of Life':ad,ti) AND ('Hormone Replacement':ad,ti OR 'Estrogen therapy':ad,ti OR 'Hormone therapy':ad,ti OR 'Estradiol':ad,ti OR 'Phytoestrogen':ad,ti OR 'Estradiol Valerate':ad,ti OR 'Estradiol Benzoate':ad,ti OR 'Estradiol Cypionate':ad,ti OR 'Chlorotrianisene':ad,ti OR 'Promestriene':ad,ti OR 'Hexestrol':ad,ti OR 'Ethinylestradiol':ad,ti OR 'Nilestriol':ad,ti OR 'Conjugated Estrogens':ad,ti OR 'Diethylstilbestrol':ad,ti OR 'Megestrol':ad,ti OR 'Norgestrel':ad,ti OR 'Norethindrone':ad,ti OR 'Levonorgestrel':ad,ti OR 'Desogestrel':ad,ti OR 'Gestrinone':ad,ti OR 'Anordrin':ad,ti OR 'Nonoxynol':ad,ti OR 'Tibolone':ad,ti OR 'Medroxyprogesterone':ad,ti OR 'Promestriene':ad,ti OR 'Cyproterone':ad,ti OR 'Promegestone':ad,ti OR 'Nomegestrol':ad,ti OR 'Drospirenone':ad,ti OR 'Phytoestrogen':ad,ti OR 'Phytoestrogens':ad,ti OR 'Soybeans':ad,ti OR 'Soybean':ad,ti OR 'Soy Foods':ad,ti OR 'Trifolium':ad,ti OR 'Isoflavones':ad,ti OR 'Phytooestrogen':ad,ti OR 'Phyto-estrogen':ad,ti OR 'Phyto-oestrogen':ad,ti OR 'Soy':ad,ti OR 'Red clover':ad,ti OR 'Isoflavon':ad,ti OR 'Daidzein':ad,ti OR 'Genistein':ad,ti OR 'Flaxseed':ad,ti OR 'Tofu':ad,ti OR 'Flax':ad,ti OR 'Flaxseed':ad,ti OR 'Linum usitatissimum':ad,ti) AND (Mental':ad,ti OR 'Psychological':ad,ti OR 'Emotion':ad,ti OR 'Depression':ad,ti OR 'Emotional Depression':ad,ti OR 'Symptoms, Depressive':ad,ti OR 'Depressive Symptom':ad,ti OR 'Depressive Symptoms':ad,ti OR 'Emotional Depressions':ad,ti OR 'Depressions, Emotional':ad,ti OR 'Depression':ad,ti OR 'Depressed':ad,ti OR 'Anxiety':ad,ti OR 'Anxious':ad,ti OR 'Hypervigilance':ad,ti OR 'Nervousness':ad,ti OR 'Anxiety':ad,ti OR 'Stress':ad,ti OR 'Stressful':ad,ti OR 'Sleep':ad,ti OR 'Insomnia':ad,ti OR 'Sleeplessness':ad,ti OR 'Hyposomnia':ad,ti) |
| **C****ochrane Library** |
| ((Perimenopausal):ti,ab,kw OR (Postmenopausal):ti,ab,kw OR (Post-Menopausal):ti,ab,kw OR (perimenopause):ti,ab,kw OR (Pre-Menopause):ti,ab,kw OR (Pre-menopausal):ti,ab,kw OR (Premenopausal):ti,ab,kw OR (Premenopause):ti,ab,kw OR (Postmenopause):ti,ab,kw OR (Climacteric):ti,ab,kw OR (Climacterics):ti,ab,kw OR (Change of Life):ti,ab,kw AND (('Hormone Replacement):ti,ab,kw OR (Estrogen therapy):ti,ab,kw OR (Hormone therapy):ti,ab,kw OR (Estradiol):ti,ab,kw OR (Phytoestrogen):ti,ab,kw OR (Estradiol Valerate):ti,ab,kw OR (Estradiol Benzoate):ti,ab,kw OR (Estradiol Cypionate):ti,ab,kw OR (Chlorotrianisene):ti,ab,kw OR (Promestriene):ti,ab,kw OR (Hexestrol):ti,ab,kw OR (Ethinylestradiol):ti,ab,kw OR (Nilestriol):ti,ab,kw OR (Conjugated Estrogens):ti,ab,kw OR (Diethylstilbestrol):ti,ab,kw OR (Megestrol):ti,ab,kw OR (Norgestrel):ti,ab,kw OR (Norethindrone):ti,ab,kw OR (Levonorgestrel):ti,ab,kw OR (Desogestrel):ti,ab,kw OR (Gestrinone):ti,ab,kw OR (Anordrin):ti,ab,kw OR (Nonoxynol):ti,ab,kw OR (Tibolone):ti,ab,kw OR (Medroxyprogesterone):ti,ab,kw OR (Promestriene):ti,ab,kw OR (Cyproterone):ti,ab,kw OR (Promegestone):ti,ab,kw OR (Nomegestrol):ti,ab,kw OR (Drospirenone):ti,ab,kw OR (Phytoestrogen):ti,ab,kw OR (Phytoestrogens):ti,ab,kw OR (Soybeans):ti,ab,kw OR (Soybean):ti,ab,kw OR (Soy Foods):ti,ab,kw OR (Trifolium):ti,ab,kw OR (Isoflavones):ti,ab,kw OR (Phytooestrogen):ti,ab,kw OR (Phyto-estrogen):ti,ab,kw OR (Phyto-oestrogen):ti,ab,kw OR (Soy):ti,ab,kw OR (Red clover):ti,ab,kw OR (Isoflavon):ti,ab,kw OR (Daidzein):ti,ab,kw OR (Genistein):ti,ab,kw OR (Flaxseed):ti,ab,kw OR (Tofu):ti,ab,kw OR (Flax):ti,ab,kw OR (Flaxseed):ti,ab,kw OR (Linum usitatissimum':ad,ti) AND ((Mental):ti,ab,kw OR (Psychological):ti,ab,kw OR (Emotion):ti,ab,kw OR (Depression):ti,ab,kw OR (Emotional Depression):ti,ab,kw OR (Symptoms, Depressive):ti,ab,kw OR (Depressive Symptom):ti,ab,kw OR (Depressive Symptoms):ti,ab,kw OR (Emotional Depressions):ti,ab,kw OR (Depressions, Emotional):ti,ab,kw OR (Depression):ti,ab,kw OR (Depressed):ti,ab,kw OR (Anxiety):ti,ab,kw OR (Anxious):ti,ab,kw OR (Hypervigilance):ti,ab,kw OR (Nervousness):ti,ab,kw OR (Anxiety):ti,ab,kw OR (Stress):ti,ab,kw OR (Stressful):ti,ab,kw OR (Sleep):ti,ab,kw OR (Insomnia):ti,ab,kw OR (Sleeplessness):ti,ab,kw OR (Hyposomnia):ti,ab,kw) |
| **ClinicalTrials.gov** |
| Condition/disease: mental, Psychological, Psychic, psychictrauma, psychic trauma, mentality, psycho, psychosis, psychic, psychology, mentality, psychosocial, Emotional, Emotions, Emotion, Depressions, Depression, Emotional, Symptom, Depressive, Emotional Depression, Symptoms, Depressive, Depressive Symptom, Depressive Symptoms, Emotional Depressions, Depressions, Emotional, Depression, Depressed  Intervention/treatment: Depressions, Depression, Emotional, Symptom, Depressive, Emotional Depression, Symptoms, Depressive, Depressive Symptom, Depressive Symptoms, Emotional Depressions, Depressions, Emotional, Depression, Depressed |
| **EMBASE** |
| 'Perimenopausal':ad,ti OR 'Postmenopausal':ad,ti OR 'Post-Menopausal':ad,ti OR 'perimenopause':ad,ti OR 'Pre-Menopause':ad,ti OR 'Pre-menopausal':ad,ti OR 'Premenopausal':ad,ti OR 'Premenopause':ad,ti OR 'Postmenopause':ad,ti OR 'Climacteric':ad,ti OR 'Climacterics':ad,ti OR 'Change of Life':ad,ti) AND ('Hormone Replacement':ad,ti OR 'Estrogen therapy':ad,ti OR 'Hormone therapy':ad,ti OR 'Estradiol':ad,ti OR 'Phytoestrogen':ad,ti OR 'Estradiol Valerate':ad,ti OR 'Estradiol Benzoate':ad,ti OR 'Estradiol Cypionate':ad,ti OR 'Chlorotrianisene':ad,ti OR 'Promestriene':ad,ti OR 'Hexestrol':ad,ti OR 'Ethinylestradiol':ad,ti OR 'Nilestriol':ad,ti OR 'Conjugated Estrogens':ad,ti OR 'Diethylstilbestrol':ad,ti OR 'Megestrol':ad,ti OR 'Norgestrel':ad,ti OR 'Norethindrone':ad,ti OR 'Levonorgestrel':ad,ti OR 'Desogestrel':ad,ti OR 'Gestrinone':ad,ti OR 'Anordrin':ad,ti OR 'Nonoxynol':ad,ti OR 'Tibolone':ad,ti OR 'Medroxyprogesterone':ad,ti OR 'Promestriene':ad,ti OR 'Cyproterone':ad,ti OR 'Promegestone':ad,ti OR 'Nomegestrol':ad,ti OR 'Drospirenone':ad,ti OR 'Phytoestrogen':ad,ti OR 'Phytoestrogens':ad,ti OR 'Soybeans':ad,ti OR 'Soybean':ad,ti OR 'Soy Foods':ad,ti OR 'Trifolium':ad,ti OR 'Isoflavones':ad,ti OR 'Phytooestrogen':ad,ti OR 'Phyto-estrogen':ad,ti OR 'Phyto-oestrogen':ad,ti OR 'Soy':ad,ti OR 'Red clover':ad,ti OR 'Isoflavon':ad,ti OR 'Daidzein':ad,ti OR 'Genistein':ad,ti OR 'Flaxseed':ad,ti OR 'Tofu':ad,ti OR 'Flax':ad,ti OR 'Flaxseed':ad,ti OR 'Linum usitatissimum':ad,ti) AND (Mental':ad,ti OR 'Psychological':ad,ti OR 'Emotion':ad,ti OR 'Depression':ad,ti OR 'Emotional Depression':ad,ti OR 'Symptoms, Depressive':ad,ti OR 'Depressive Symptom':ad,ti OR 'Depressive Symptoms':ad,ti OR 'Emotional Depressions':ad,ti OR 'Depressions, Emotional':ad,ti OR 'Depression':ad,ti OR 'Depressed':ad,ti OR 'Anxiety':ad,ti OR 'Anxious':ad,ti OR 'Hypervigilance':ad,ti OR 'Nervousness':ad,ti OR 'Anxiety':ad,ti OR 'Stress':ad,ti OR 'Stressful':ad,ti OR 'Sleep':ad,ti OR 'Insomnia':ad,ti OR 'Sleeplessness':ad,ti OR 'Hyposomnia':ad,ti) |

### eTable 3. Table of subgroup classification

| **Regions** | | | |
| --- | --- | --- | --- |
| Australia | Almeida et al., 2006 | China | Haines et al.,2003 |
|  | Evans et al., 2017 |  | Zhang et al., 2021 |
|  | Khoo et al., 1998 |  | Liu et al., 2024 |
|  | Kotsopoulos et al., 2000 | Japan | Hirose et al., 2016 |
|  | Kulkarni et al., 2018 |  | Ishiwata et al., 2023 |
| Austria | Lipovac et al., 2010 | Korea | Park et al., 2016 |
|  | Saletu et al., 1995 | Multi-nations | Sismondi et al., 2011 |
|  | Saletu-Zyhlarz et al.,2003 | Sweden | Wiklund et al., 1993 |
| Brazil | Demetrio et al; 2011 | Thailand | Tansupswatdikul et al., 2015 |
|  | Frigo et al; 2022 | Turkey | Baksu et al., 2005 |
|  | Hachul et al., 2011 |  | Baksu et al; 2009 |
|  | Silva et al., 2011 |  | Gülseren et al., 2005 |
|  | Sousa et al., 2009 | UK | Pearce et al., 1996 |
| Canada | Gordon et al., 2018 |  | Purdie et al., 1995 |
| Mexico | Carranza et al., 1999 |  | Welton et al., 2008 |
|  | Berlanga et al., 2003 | USA | Block et al., 1981 |
| Denmark | Bech et al., 1998 |  | Brunner et al., 2005 |
|  | Nielsen et al., 2006 |  | Caan et al., 2015 |
| Germany | Heinrich et al., 2005 |  | Cintron et al., 2018 |
|  | Rudolph et al., 2004 |  | Diem et al., 2018 |
|  | Schüssler et al., 2008 |  | Ensrud et al., 2015 |
| Holland | Meeuwsen et al., 2002 |  | Girdler et al., 1999 |
| India | Khanna et al., 2020 |  | Gleason et al., 2015 |
|  | Shamshad et al., 2016 |  | Hays et al., 2003 |
| Iran | Aghamiri et al., 2016 |  | Hlatky et al., 2002 |
| Italy | Atteritano et al., 2014 |  | Joffe et al., 2011 |
|  | Cagnacci et al., 2003 |  | Kagan et al., 2018 |
|  | Casini et al., 2006 |  | LeBlanc et al., 2007 |
|  | Gambacciani et al.,2003 |  | Morrison et al., 2004 |
|  | Gambacciani et al.,2005 |  | Rasgon et al; 2007 |
|  | Gambacciani et al.,2011 |  | Schmidt et al., 2004 |
|  | Paoletti et al., 2001 |  | Schmidt et al., 2021 |
|  | Zanardi et al., 2007 |  | Soares et al., 2001 |

Abbreviations: USA=The United States of America; UK=The United Kingdom.

| **Participants** | | | |
| --- | --- | --- | --- |
| Perimenopause | Aghamiri et al., 2016 | Postmenopause | Gambacciani et al.,2011 |
|  | Liu et al., 2024 |  | Girdler et al., 1999 |
|  | Hachul et al., 2011 |  | Gleason et al., 2015 |
|  | Khoo et al., 1998 |  | Gordon et al., 2018 |
|  | Kotsopoulos et al., 2000 |  | Haines et al.,2003 |
|  | LeBlanc et al., 2007 |  | Hirose et al., 2016 |
|  | Lipovac et al., 2010 |  | Hlatky et al., 2002 |
|  | Meeuwsen et al., 2002 |  | Ishiwata et al., 2023 |
|  | Morrison et al., 2004 |  | Joffe et al., 2011 |
|  | Paoletti et al., 2001 |  | Kagan et al., 2018 |
|  | Park et al., 2016 |  | Khanna et al., 2020 |
|  | Rasgon et al; 2007 |  | Kulkarni et al., 2018 |
|  | Saletu-Zyhlarz et al.,2003 |  | Pearce et al., 1996 |
|  | Schmidt et al., 2004 |  | Purdie et al., 1995 |
|  | Schmidt et al., 2021 |  | Rudolph et al., 2004 |
|  | Schüssler et al., 2008 |  | Saletu et al., 1995 |
|  | Tansupswatdikul et al., 2015 |  | Schmidt et al., 2021 |
| Postmenopause | Almeida et al., 2006 |  | Shamshad et al., 2016 |
|  | Atteritano et al., 2014 |  | Silva et al., 2011 |
|  | Baksu et al., 2005 |  | Sismondi et al., 2011 |
|  | Baksu et al; 2009 |  | Soares et al., 2001 |
|  | Bech et al., 1998 |  | Sousa et al., 2009 |
|  | Berlanga et al., 2003 |  | Zanardi et al., 2007 |
|  | Block et al., 1981 |  | Zhang et al., 2021 |
|  | Caan et al., 2015 | Peri- and post-Menopause | Baksu et al., 2005 |
|  | Carranza et al., 1999 |  | Cagnacci et al., 2003 |
|  | Casini et al., 2006 |  | Gülseren et al., 2005 |
|  | Cintron et al., 2018 |  | Welton et al., 2008 |
|  | Demetrio et al; 2011 |  | Wiklund et al., 1993 |
|  | Diem et al., 2018 | Surgical menopause | Gambacciani et al.,2003 |
|  | Ensrud et al., 2015 |  | Hays et al., 2003 |
|  | Evans et al., 2017 |  | Heinrich et al., 2005 |
|  | Frigo et al; 2022 |  | Nielsen et al., 2006 |
|  | Gambacciani et al.,2005 |  | Paoletti et al., 2001 |

| **Interventions** | | | |
| --- | --- | --- | --- |
| EPT | Baksu et al., 2005 | Estrogen | Heinrich et al., 2005 |
|  | Baksu et al; 2009 |  | Hirose et al., 2016 |
|  | Liu et al., 2024 |  | Ishiwata et al., 2023 |
|  | Block et al., 1981 |  | Kagan et al., 2018 |
|  | Cintron et al., 2018 |  | Kotsopoulos et al., 2000 |
|  | Demetrio et al; 2011 |  | Morrison et al., 2004 |
|  | Ensrud et al., 2015 |  | Paoletti et al., 2001 |
|  | Frigo et al; 2022 |  | Park et al., 2016 |
|  | Gambacciani et al.,2011 |  | Purdie et al., 1995 |
|  | Gleason et al., 2015 |  | Rasgon et al; 2007 |
|  | Gordon et al., 2018 |  | Rudolph et al., 2004 |
|  | Hachul et al., 2011 |  | Saletu et al., 1995 |
|  | Hlatky et al., 2002 | Phytoestrogen | Saletu-Zyhlarz et al.,2003 |
|  | Joffe et al., 2011 |  | Schüssler et al., 2008 |
|  | Khanna et al., 2020 |  | Schmidt et al., 2004 |
|  | Khoo et al., 1998 |  | Schmidt et al., 2021 |
|  | Lipovac et al., 2010 |  | Shamshad et al., 2016 |
|  | Meeuwsen et al., 2002 |  | Silva et al., 2011 |
|  | Pearce et al., 1996 |  | Sismondi et al., 2011 |
| Estrogen | Almeida et al., 2006 |  | Soares et al., 2001 |
|  | Baksu et al., 2005 |  | Sousa et al., 2009 |
|  | Baksu et al; 2009 |  | Tansupswatdikul et al., 2015 |
|  | Berlanga et al., 2003 |  | Welton et al., 2008 |
|  | Brunner et al., 2005 |  | Wiklund et al., 1993 |
|  | Caan et al., 2015 |  | Zanardi et al., 2007 |
|  | Cagnacci et al., 2003 |  | Zhang et al., 2021 |
|  | Casini et al., 2006 | Progesterone | Aghamiri et al., 2016 |
|  | Cintron et al., 2018 |  | Diem et al., 2018 |
|  | Gülseren et al., 2005 | Tibolone | Atteritano et al., 2014 |
|  | Gambacciani et al.,2003 |  | Carranza et al., 1999 |
|  | Gambacciani et al.,2005 |  | Evans et al., 2017 |
|  | Girdler et al., 1999 |  | Kulkarni et al., 2018 |
|  | Gleason et al., 2015 |  | LeBlanc et al., 2007 |
|  | Haines et al.,2003 |  | Nielsen et al., 2006 |
|  | Hays et al., 2003 |  | Paoletti et al., 2001 |

Abbreviation: EPT=Estrogen plus progesterone therapy.

| **Route of administration** | | | |
| --- | --- | --- | --- |
| Intranasal | Casini et al., 2006 | Oral | Nielsen et al., 2006 |
|  | Heinrich et al., 2005 |  | Paoletti et al., 2001 |
| Oral | Aghamiri et al., 2016 |  | Pearce et al., 1996 |
|  | Almeida et al., 2006 |  | Saletu-Zyhlarz et al.,2003 |
|  | Atteritano et al., 2014 |  | Schüssler et al., 2008 |
|  | Baksu et al., 2005 |  | Schmidt et al., 2004 |
|  | Baksu et al; 2009 |  | Schmidt et al., 2021 |
|  | Bech et al., 1998 |  | Shamshad et al., 2016 |
|  | Berlanga et al., 2003 |  | Silva et al., 2011 |
|  | Block et al., 1981 |  | Sismondi et al., 2011 |
|  | Brunner et al., 2005 |  | Soares et al., 2001 |
|  | Cagnacci et al., 2003 |  | Sousa et al., 2009 |
|  | Carranza et al., 1999 |  | Tansupswatdikul et al., 2015 |
|  | Cintron et al., 2018 |  | Welton et al., 2008 |
|  | Demetrio et al; 2011 |  | Wiklund et al., 1993 |
|  | Diem et al., 2018 |  | Zanardi et al., 2007 |
|  | Ensrud et al., 2015 |  | Zhang et al., 2021 |
|  | Evans et al., 2017 | Transdermal | Haines et al.,2003 |
|  | Frigo et al; 2022 |  | Heinrich et al., 2005 |
|  | Gülseren et al., 2005 |  | Hirose et al., 2016 |
|  | Gambacciani et al.,2003 |  | Ishiwata et al., 2023 |
|  | Gambacciani et al.,2005 |  | Kagan et al., 2018 |
|  | Gambacciani et al.,2011 |  | Khoo et al., 1998 |
|  | Girdler et al., 1999 |  | Kotsopoulos et al., 2000 |
|  | Gleason et al., 2015 |  | Meeuwsen et al., 2002 |
|  | Gordon et al., 2018 |  | Morrison et al., 2004 |
|  | Hachul et al., 2011 |  | Paoletti et al., 2001 |
|  | Hays et al., 2003 |  | Park et al., 2016 |
|  | Hlatky et al., 2002 |  | Purdie et al., 1995 |
|  | Joffe et al., 2011 |  | Rasgon et al; 2007 |
|  | Khanna et al., 2020 |  | Rudolph et al., 2004 |
|  | Kulkarni et al., 2018 |  | Saletu et al., 1995 |
|  | LeBlanc et al., 2007 | Vaginal | Caan et al., 2015 |
|  | Lipovac et al., 2010 |  | |

| **Duration** | | | |
| --- | --- | --- | --- |
| Long term | Almeida et al., 2006 | Short term | Aghamiri et al., 2016 |
|  | Casini et al., 2006 |  | Baksu et al., 2005 |
|  | Evans et al., 2017 |  | Baksu et al; 2009 |
|  | Gordon et al., 2018 |  | Brunner et al., 2005 |
|  | Lipovac et al., 2010 |  | Caan et al., 2015 |
|  | Meeuwsen et al., 2002 |  | Cagnacci et al., 2003 |
|  | Paoletti et al., 2001 |  | Cintron et al., 2018 |
|  | Park et al., 2016 |  | Demetrio et al; 2011 |
|  | Soares et al., 2001 |  | Diem et al., 2018 |
| Moderate term | Atteritano et al., 2014 |  | Ensrud et al., 2015 |
|  | Bech et al., 1998 |  | Gülseren et al., 2005 |
|  | Berlanga et al., 2003 |  | Haines et al.,2003 |
|  | Block et al., 1981 |  | Hirose et al., 2016 |
|  | Carranza et al., 1999 |  | Hlatky et al., 2002 |
|  | Frigo et al; 2022 |  | Ishiwata et al., 2023 |
|  | Gambacciani et al.,2003 |  | Joffe et al., 2011 |
|  | Gambacciani et al.,2005 |  | Kagan et al., 2018 |
|  | Gambacciani et al.,2011 |  | Khoo et al., 1998 |
|  | Girdler et al., 1999 |  | Kotsopoulos et al., 2000 |
|  | Gleason et al., 2015 |  | Kulkarni et al., 2018 |
|  | Hachul et al., 2011 |  | LeBlanc et al., 2007 |
|  | Hays et al., 2003 |  | Morrison et al., 2004 |
|  | Heinrich et al., 2005 |  | Purdie et al., 1995 |
|  | Khanna et al., 2020 |  | Rudolph et al., 2004 |
|  | Nielsen et al., 2006 |  | Saletu et al., 1995 |
|  | Paoletti et al., 2001 |  | Saletu-Zyhlarz et al.,2003 |
|  | Pearce et al., 1996 |  | Schüssler et al., 2008 |
|  | Rasgon et al; 2007 |  | Schmidt et al., 2004 |
|  | Saletu-Zyhlarz et al.,2003 |  | Schmidt et al., 2021 |
|  | Silva et al., 2011 |  | Shamshad et al., 2016 |
|  | Sismondi et al., 2011 |  | Tansupswatdikul et al., 2015 |
|  | Sousa et al., 2009 |  | Welton et al., 2008 |
|  | Zanardi et al., 2007 |  | Wiklund et al., 1993 |
|  | Zhang et al., 2021 |  | Liu et al., 2024 |

### eTable 4. Quality assessment of included studies

| **References (author, year)** | **Random sequence generation (selection bias)** | **Allocation concealment (selection bias)** | **Blinding of participants and personnel (performance bias)** | **Blinding of outcome assessment (detection bias)** | **Incomplete outcome data (attrition bias)** | **Selective reporting (reporting bias)** | **Other bias** |
| --- | --- | --- | --- | --- | --- | --- | --- |
| Block et al., 1981 | L | L | H | L | L | L | L |
| Liu et al., 2024 | L | L | U | L | L | L | L |
| Brunner et al., 2005 | L | L | U | L | L | L | L |
| Gülseren et al., 2005 | L | L | L | L | L | L | L |
| Ensrud et al., 2015 | L | L | L | L | L | U | U |
| Gambacciani et al.,2003 | L | L | L | U | L | L | L |
| Gambacciani et al.,2005 | L | L | U | L | L | L | L |
| Gambacciani et al.,2011 | L | L | H | U | L | L | L |
| Caan et al., 2015 | L | L | L | L | L | L | U |
| Hays et al., 2003 | L | U | L | L | L | L | L |
| Heinrich et al., 2005 | L | L | L | H | L | L | L |
| Joffe et al., 2011 | L | H | L | L | L | L | L |
| Diem et al., 2018 | L | L | H | L | L | L | L |
| LeBlanc et al., 2007 | L | L | L | U | L | H | L |
| Khanna et al., 2020 | L | U | L | L | L | L | H |
| Meeuwsen et al., 2002 | L | U | L | L | L | U | U |
| Nielsen et al., 2006 | L | U | L | L | U | H | L |
| Cagnacci et al., 2003 | L | L | L | H | L | U | L |
| Purdie et al., 1995 | L | L | L | L | L | L | L |
| Hirose et al., 2016 | L | H | L | L | L | L | L |
| Saletu-Zyhlarz et al.,2003 | L | L | H | L | L | L | U |
| Schüssler et al., 2008 | L | L | U | L | L | L | L |
| Silva et al., 2011 | L | L | L | L | L | L | L |
| Sismondi et al., 2011 | L | L | U | H | L | L | L |
| Ishiwata et al., 2023 | L | L | L | U | L | L | L |
| Welton et al., 2008 | L | L | U | L | L | L | L |
| Zhang et al., 2021 | L | L | H | U | H | L | L |
| Carranza et al., 1999 | L | L | L | L | L | L | U |
| Hachul et al., 2011 | L | U | L | L | L | L | L |
| Almeida et al., 2006 | L | L | L | H | L | L | L |
| Bech et al., 1998 | L | H | L | L | L | L | L |
| Demetrio et al; 2011 | L | L | H | L | L | L | L |
| Girdler et al., 1999 | L | L | L | U | L | H | L |
| Haines et al.,2003 | L | U | L | L | L | L | H |
| Hlatky et al., 2002 | L | U | L | H | H | U | U |
| Khoo et al., 1998 | L | U | L | L | U | H | L |
| Pearce et al., 1996 | L | L | U | H | L | H | L |
| Baksu et al; 2009 | L | L | L | L | L | L | L |
| Frigo et al; 2022 | L | H | L | L | L | L | L |
| Rasgon et al; 2007 | L | L | H | L | U | H | L |
| Zanardi et al., 2007 | L | L | U | L | L | L | L |
| Paoletti et al., 2001 | L | L | L | L | L | L | L |
| Morrison et al., 2004 | L | H | L | L | L | U | L |
| Rudolph et al., 2004 | L | L | L | U | L | L | L |
| Schmidt et al., 2004 | L | L | L | L | L | L | L |
| Soares et al., 2001 | L | L | H | U | H | L | L |
| Casini et al., 2006 | L | L | L | L | L | L | U |
| Berlanga et al., 2003 | L | U | L | L | U | L | L |
| Kulkarni et al., 2018 | L | L | L | H | L | L | L |
| Gleason et al., 2015 | L | H | L | L | L | L | L |
| Schmidt et al., 2021 | L | L | H | L | L | L | L |
| Baksu et al., 2005 | L | L | L | U | L | H | L |
| Cintron et al., 2018 | L | U | L | L | H | L | H |
| Kagan et al., 2018 | L | U | L | L | L | U | U |
| Tansupswatdikul et al., 2015 | L | U | L | L | U | H | L |
| Gordon et al., 2018 | L | L | U | H | L | U | L |
| Aghamiri et al., 2016 | L | L | L | L | L | L | L |
| Shamshad et al., 2016 | L | H | H | L | L | L | L |
| Park et al., 2016 | L | L | H | L | L | L | L |
| Saletu et al., 1995 | L | H | U | L | H | L | H |
| Kotsopoulos et al., 2000 | L | L | L | L | L | L | L |
| Evans et al., 2017 | L | L | L | H | L | L | L |
| Sousa et al., 2009 | L | L | L | U | L | L | L |
| Wiklund et al., 1993 | L | L | U | L | L | L | L |
| Atteritano et al., 2014 | L | L | L | U | H | L | L |
| Lipovac et al., 2010 | L | L | L | L | L | L | U |
| Chen., 2025 | L | L | U | L | L | L | L |

Abbreviations: H=High Risk; L=Low Risk; U=Unclear Risk.

### eTable 5. Classification effect and source of heterogeneity

| **Symptoms** | **Interventions** | **No effect** | **Small effect** | **Moderate effect** | **Source of heterogeneity** |
| --- | --- | --- | --- | --- | --- |
| **Mood** | Hormone therapy |  |  | √ | Duration |
|  | Phytoestrogens |  | √ |  | Duration |
| **Depression** | Hormone therapy |  |  | √ | Interventions |
|  | Phytoestrogens |  |  | √ | Participants |
| **Anxiety** | Hormone therapy |  |  | √ | Delivery |
|  | Phytoestrogens |  |  | √ | Participants |
| **Sleep** | Hormone therapy |  |  | √ | Participants |
|  | Phytoestrogens |  | √ |  | Duration |
| **Stress** | Hormone therapy |  | √ |  | No |
|  | Phytoestrogens |  | √ |  | No |
| **Anger** | Hormone therapy |  | √ |  | Interventions |
|  | Phytoestrogens |  |  | √ | No |
| **Phobic** | Hormone therapy | √ |  |  | No |
|  | Phytoestrogens |  |  | √ | No |
| **Well-being** | Hormone therapy |  | √ |  | No |
|  | Phytoestrogens | √ |  |  | No |
| **Quality of Life** | Hormone therapy |  |  | √ | Regions |
|  | Phytoestrogens | - | | | |

### eTable 6. Preferred reporting items for systematic reviews and meta-analyses

| **Section and Topic** | **Item #** | **Checklist item** | **Location where item is reported** |
| --- | --- | --- | --- |
| **TITLE** | | |  |
| Title | 1 | Identify the report as a systematic review. | Title |
| **ABSTRACT** | | |  |
| Abstract | 2 | Provide a structured summary including, as applicable: background; objectives; data sources; study eligibility criteria, participants, and interventions; study appraisal and synthesis methods; results; limitations; conclusions and implications of key findings. | Abstract |
| **INTRODUCTION** | | |  |
| Rationale | 3 | Describe the rationale for the review in the context of existing knowledge. | Introduction, paragraphs 1 to 3 |
| Objectives | 4 | Provide an explicit statement of the objective(s) or question(s) the review addresses. | Introduction, paragraph 4 |
| **METHODS** | | |  |
| Eligibility criteria | 5 | Specify the inclusion and exclusion criteria for the review and how studies were grouped for the syntheses. | Methods, paragraphs 3 to 4 |
| Information sources | 6 | Specify all databases, registers, websites, organisations, reference lists and other sources searched or consulted to identify studies. Specify the date when each source was last searched or consulted. | Methods, paragraph 2 |
| Search strategy | 7 | Present the full search strategies for all databases, registers and websites, including any filters and limits used. | Methods, paragraph 2 |
| Selection process | 8 | Specify the methods used to decide whether a study met the inclusion criteria of the review, including how many reviewers screened each record and each report retrieved, whether they worked independently, and if applicable, details of automation tools used in the process. | Methods, paragraph 5 |
| Data collection process | 9 | Specify the methods used to collect data from reports, including how many reviewers collected data from each report, whether they worked independently, any processes for obtaining or confirming data from study investigators, and if applicable, details of automation tools used in the process. | Methods, paragraph 5 |
| Data items | 10a | List and define all outcomes for which data were sought. Specify whether all results that were compatible with each outcome domain in each study were sought (e.g. for all measures, time points, analyses), and if not, the methods used to decide which results to collect. | Methods, paragraph 4 |
|  | 10b | List and define all other variables for which data were sought (e.g. participant and intervention characteristics, funding sources). Describe any assumptions made about any missing or unclear information. | Methods, paragraph 4 |
| Study risk of bias assessment | 11 | Specify the methods used to assess risk of bias in the included studies, including details of the tool(s) used, how many reviewers assessed each study and whether they worked independently, and if applicable, details of automation tools used in the process. | Methods, paragraphs 9 to 10 |
| Effect measures | 12 | Specify for each outcome the effect measure(s) (e.g. risk ratio, mean difference) used in the synthesis or presentation of results. | Methods, paragraph 6 |
| Synthesis methods | 13a | Describe the processes used to decide which studies were eligible for each synthesis (e.g. tabulating the study intervention characteristics and comparing against the planned groups for each synthesis (item #5)). | Methods, paragraph 6 |
|  | 13b | Describe any methods required to prepare the data for presentation or synthesis, such as handling of missing summary statistics, or data conversions. | Methods, paragraph 6 |
|  | 13c | Describe any methods used to tabulate or visually display results of individual studies and syntheses. | Methods, paragraph 6 |
|  | 13d | Describe any methods used to synthesize results and provide a rationale for the choice(s). If meta-analysis was performed, describe the model(s), method(s) to identify the presence and extent of statistical heterogeneity, and software package(s) used. | Methods, paragraph 6 |
|  | 13e | Describe any methods used to explore possible causes of heterogeneity among study results (e.g. subgroup analysis, meta-regression). | Methods, paragraphs 7 to 8 |
|  | 13f | Describe any sensitivity analyses conducted to assess robustness of the synthesized results. | Methods, paragraphs 7 |
| Reporting bias assessment | 14 | Describe any methods used to assess risk of bias due to missing results in a synthesis (arising from reporting biases). | Methods, paragraph 10 |
| Certainty assessment | 15 | Describe any methods used to assess certainty (or confidence) in the body of evidence for an outcome. | Methods, paragraph 9 |
| **RESULTS** | | |  |
| Study selection | 16a | Describe the results of the search and selection process, from the number of records identified in the search to the number of studies included in the review, ideally using a flow diagram. | Results, paragraph 1 |
|  | 16b | Cite studies that might appear to meet the inclusion criteria, but which were excluded, and explain why they were excluded. | Results, paragraph 1 |
| Study characteristics | 17 | Cite each included study and present its characteristics. | Results, paragraph 2 |
| Risk of bias in studies | 18 | Present assessments of risk of bias for each included study. | Results, paragraph 9 |
| Results of individual studies | 19 | For all outcomes, present, for each study: (a) summary statistics for each group (where appropriate) and (b) an effect estimate and it’s precision (e.g. confidence/credible interval), ideally using structured tables or plots. | Results, paragraphs 3 to 8 |
| Results of syntheses | 20a | For each synthesis, briefly summarise the characteristics and risk of bias among contributing studies. | Results, paragraph 9 |
|  | 20b | Present results of all statistical syntheses conducted. If meta-analysis was done, present for each the summary estimate and its precision (e.g. confidence/credible interval) and measures of statistical heterogeneity. If comparing groups, describe the direction of the effect. | Results, paragraphs 3 to 8 |
|  | 20c | Present results of all investigations of possible causes of heterogeneity among study results. | Results, paragraphs 3 to 8 |
|  | 20d | Present results of all sensitivity analyses conducted to assess the robustness of the synthesized results. | Results, paragraphs 3 to 8 |
| Reporting biases | 21 | Present assessments of risk of bias due to missing results (arising from reporting biases) for each synthesis assessed. | Results, paragraph 10 |
| Certainty of evidence | 22 | Present assessments of certainty (or confidence) in the body of evidence for each outcome assessed. | Results, paragraph 9 |
| **DISCUSSION** | | |  |
| Discussion | 23a | Provide a general interpretation of the results in the context of other evidence. | Discussions, paragraphs 1 to 6 |
|  | 23b | Discuss any limitations of the evidence included in the review. | Discussions, paragraph 7 |
|  | 23c | Discuss any limitations of the review processes used. | Discussions, paragraph 7 |
|  | 23d | Discuss implications of the results for practice, policy, and future research. | Conclusion, paragraph 6 |
| **OTHER INFORMATION** | | |  |
| Registration and protocol | 24a | Provide registration information for the review, including register name and registration number, or state that the review was not registered. | Methods, paragraph 1 |
|  | 24b | Indicate where the review protocol can be accessed, or state that a protocol was not prepared. | Methods, paragraph 1 |
|  | 24c | Describe and explain any amendments to information provided at registration or in the protocol. | Methods, paragraph 1 |
| Support | 25 | Describe sources of financial or non-financial support for the review, and the role of the funders or sponsors in the review. | Acknowledgments, paragraph 1 |
| Competing interests | 26 | Declare any competing interests of review authors. | Conflict of interest |
| Availability of data, code and other materials | 27 | Report which of the following are publicly available and where they can be found: template data collection forms; data extracted from included studies; data used for all analyses; analytic code; any other materials used in the review. | Supplementary Online Content |

*From:* Page MJ, McKenzie JE, Bossuyt PM, Boutron I, Hoffmann TC, Mulrow CD, et al. The PRISMA 2020 statement: an updated guideline for reporting systematic reviews. BMJ 2021;372:n71. doi: 10.1136/bmj.n71

###

### eFigure 1-17. Forest plots of meta-analysis


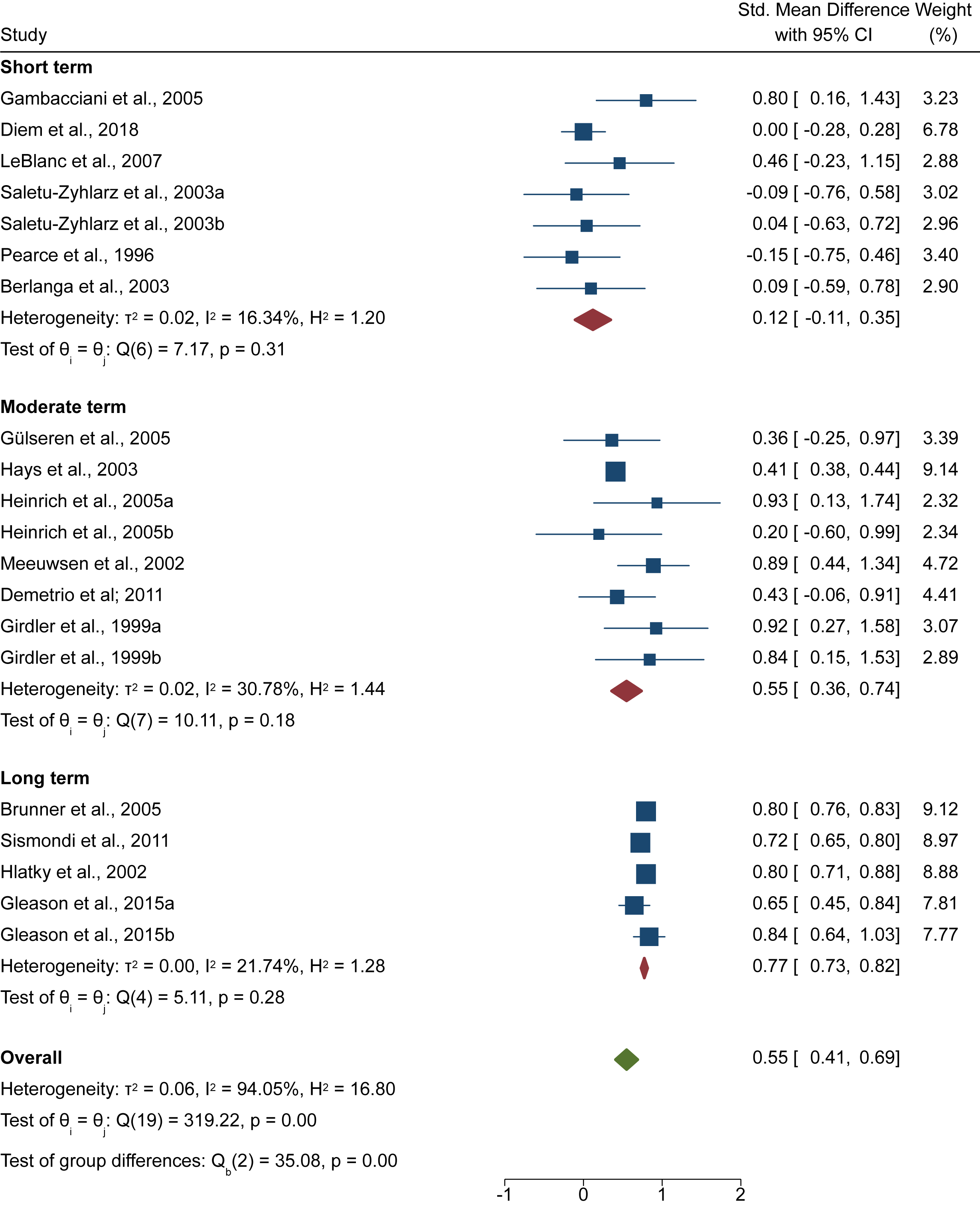
**eFigure 1. Outcomes of hormone therapy for mood**

**
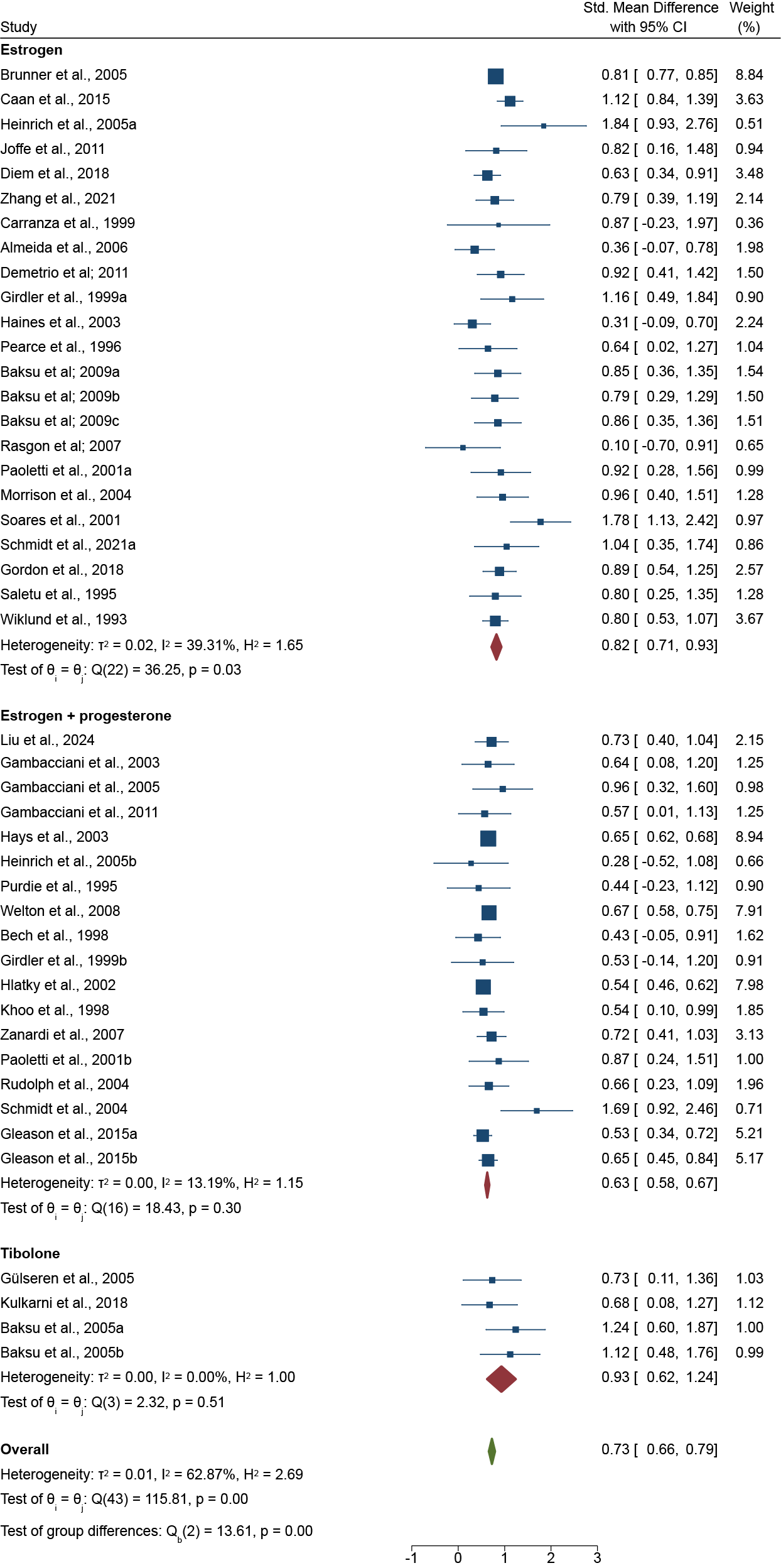
eFigure 2. Outcomes of hormone therapy for depression**

**
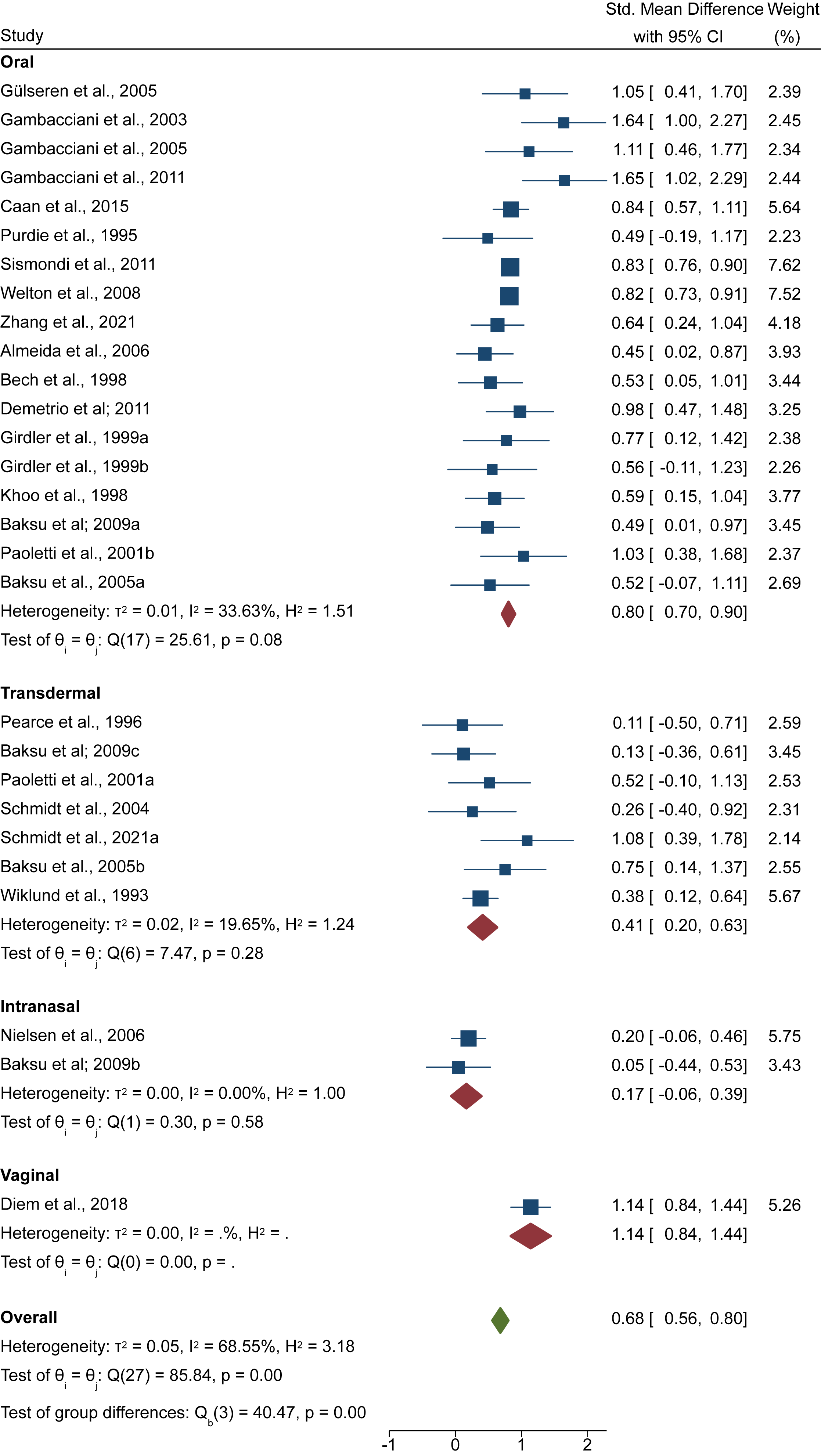
eFigure 3. Outcomes of hormone therapy for anxiety**

**
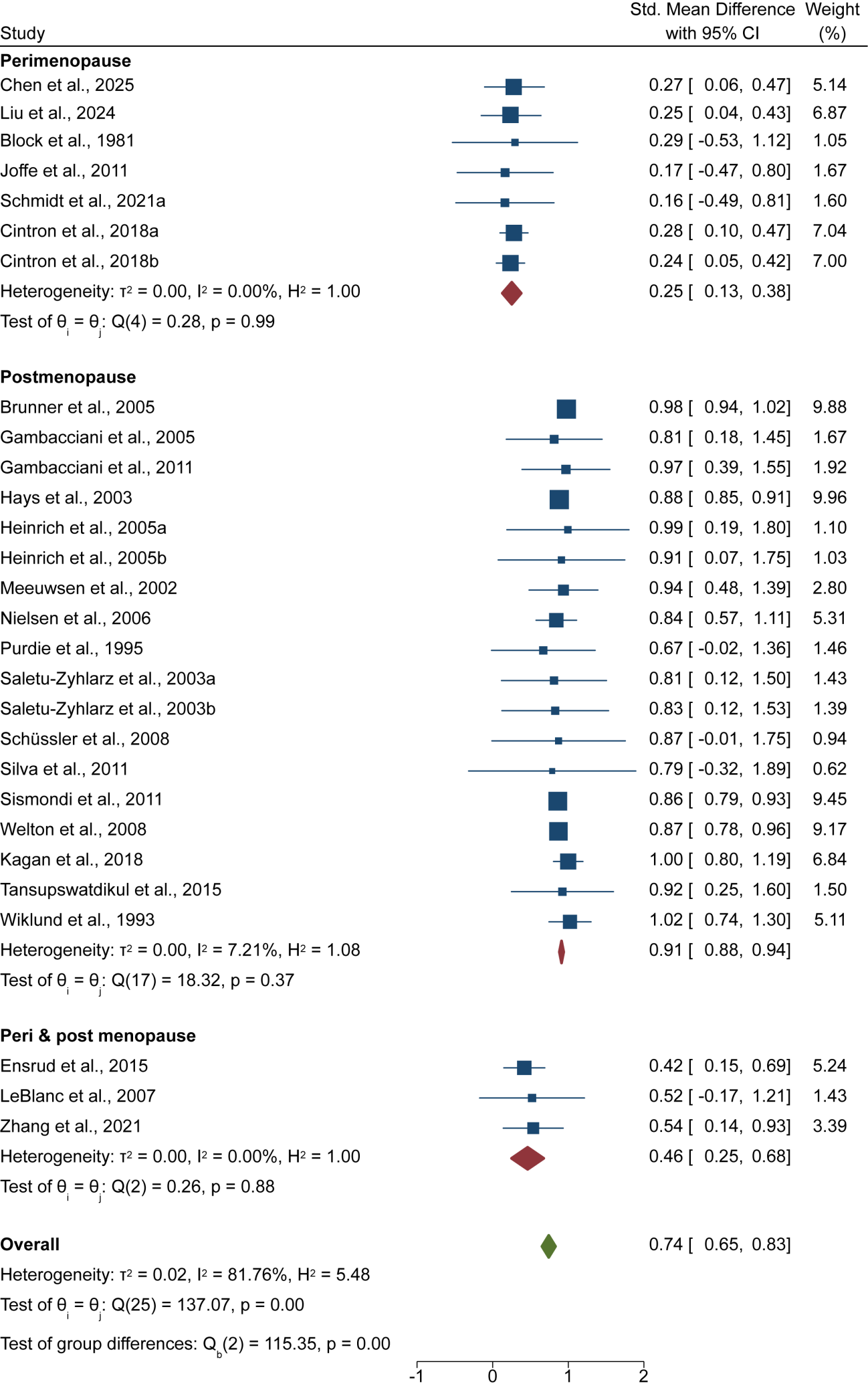
eFigure 4. Outcomes of hormone therapy for sleep**

**
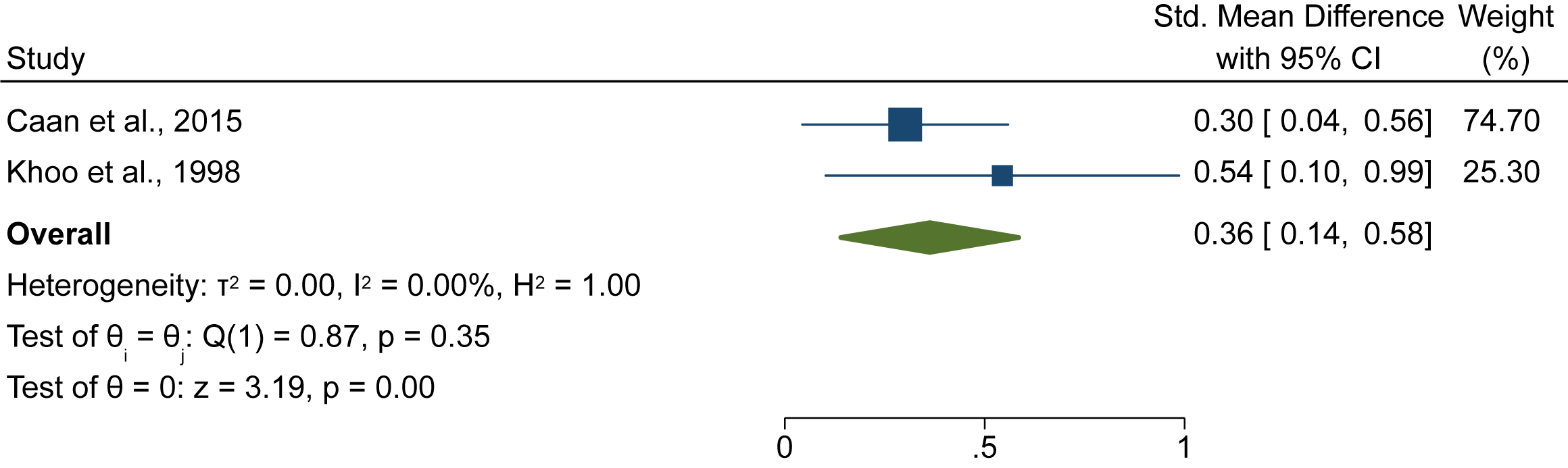
eFigure 5. Outcomes of hormone therapy for stress**

**
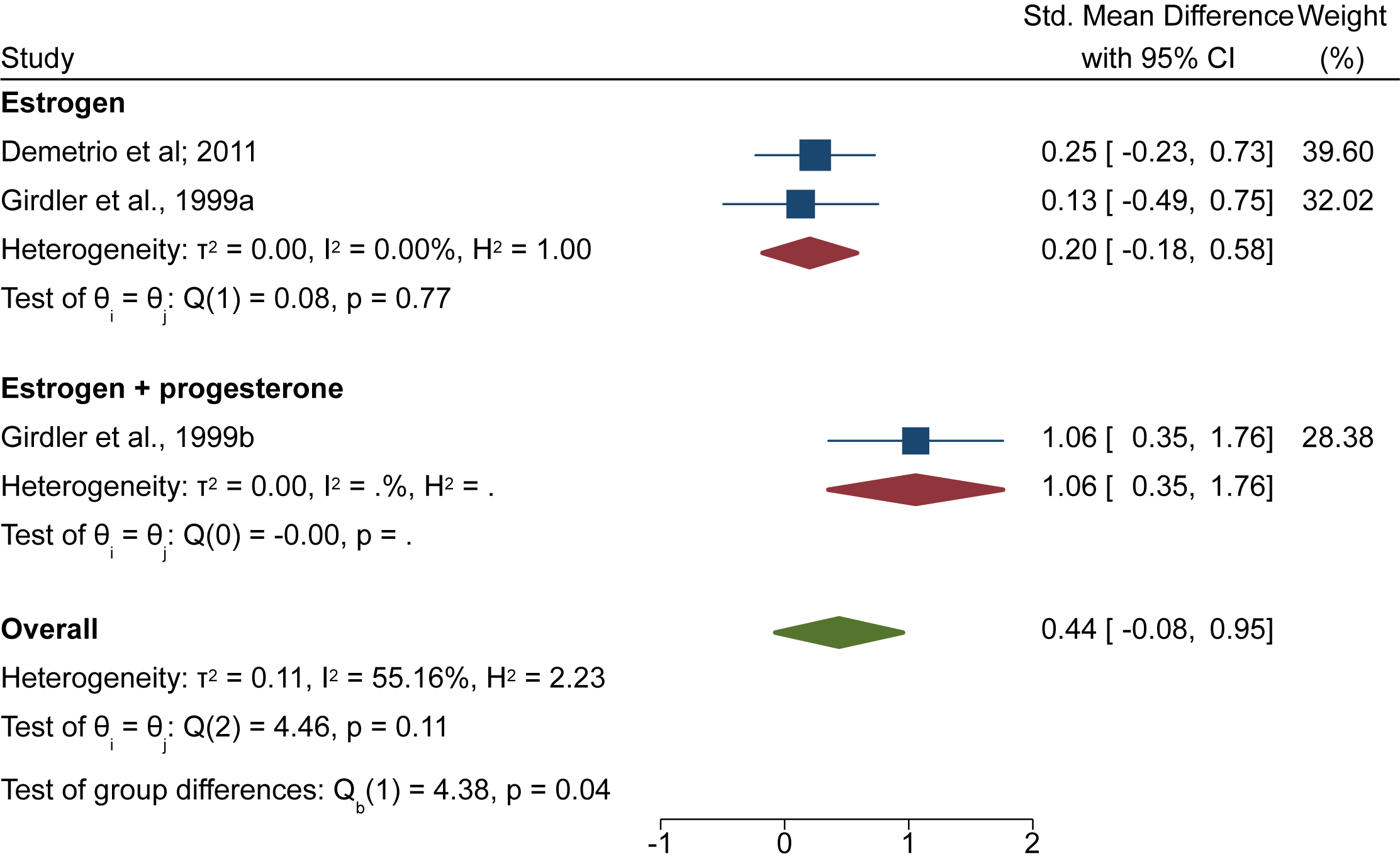
eFigure 6. Outcomes of hormone therapy for anger**

**
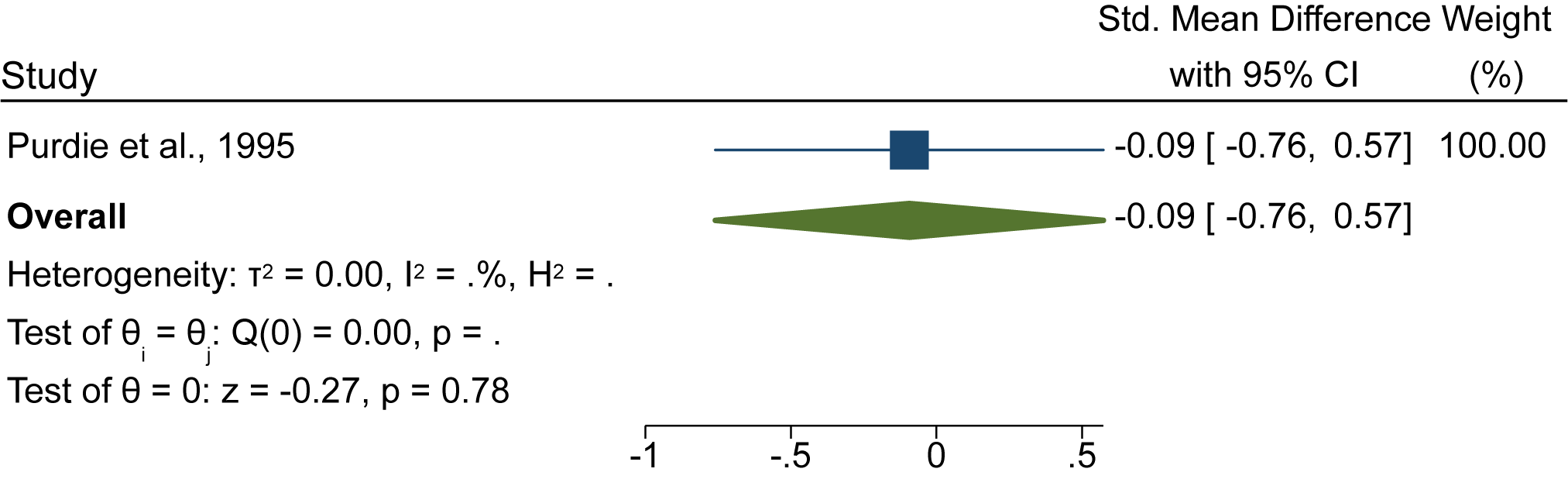
eFigure 7. Outcomes of hormone therapy for phobic**

**
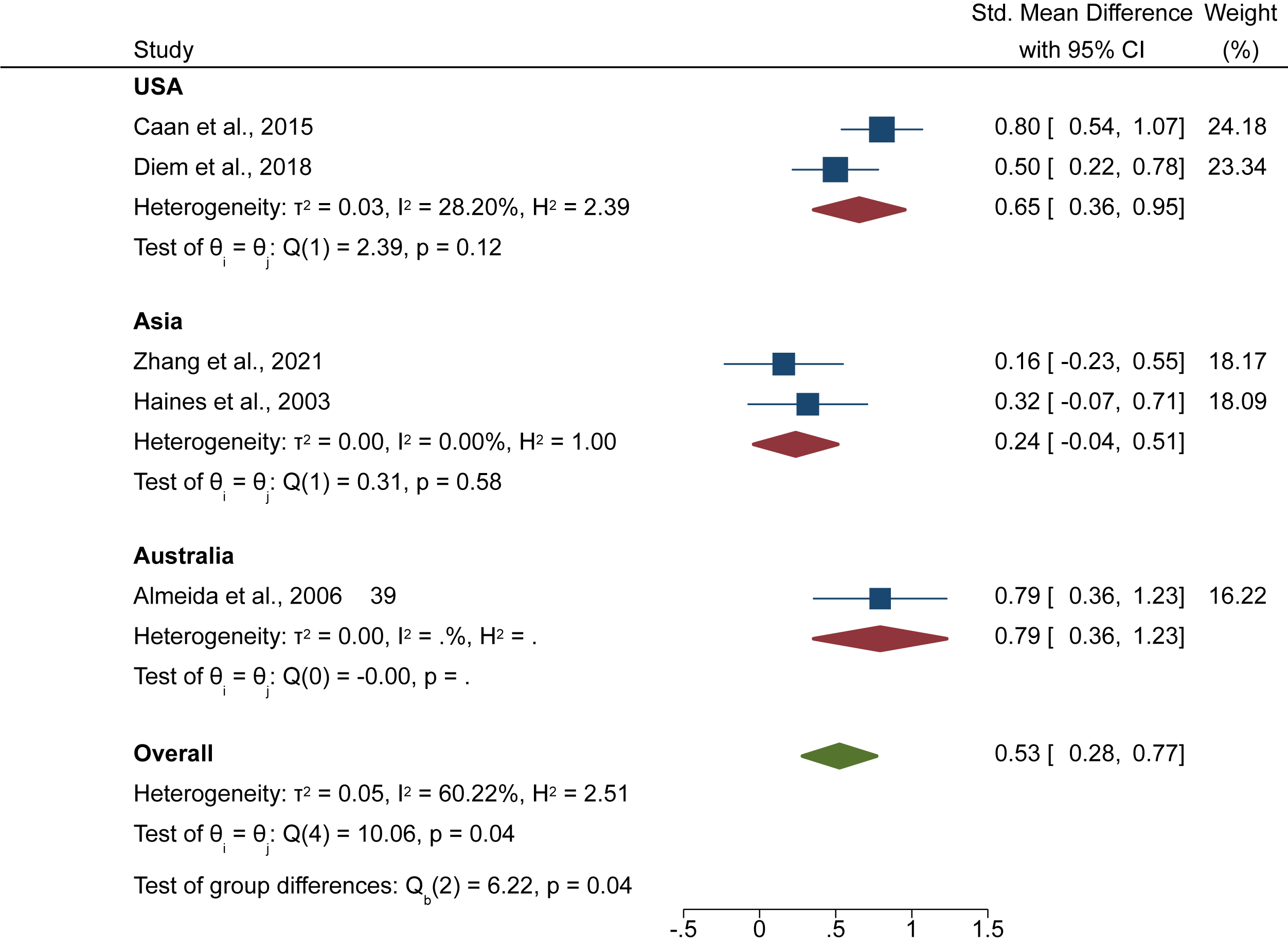
eFigure 8. Outcomes of hormone therapy for quality of life**

**
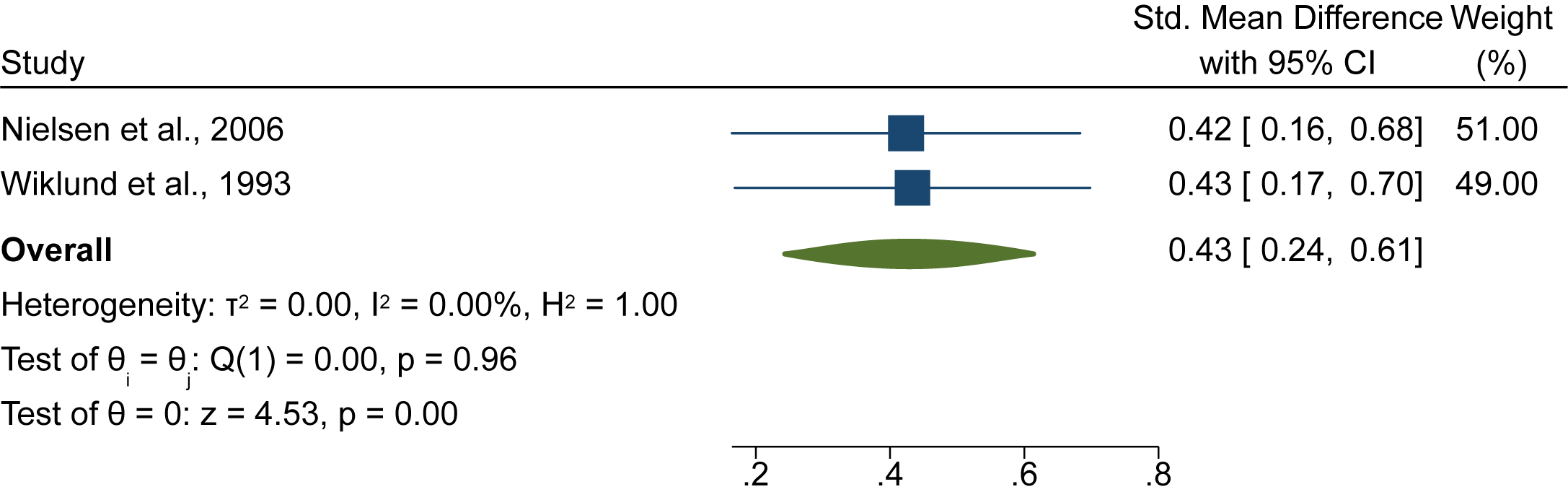
eFigure 9. Outcomes of hormone therapy for well-being**


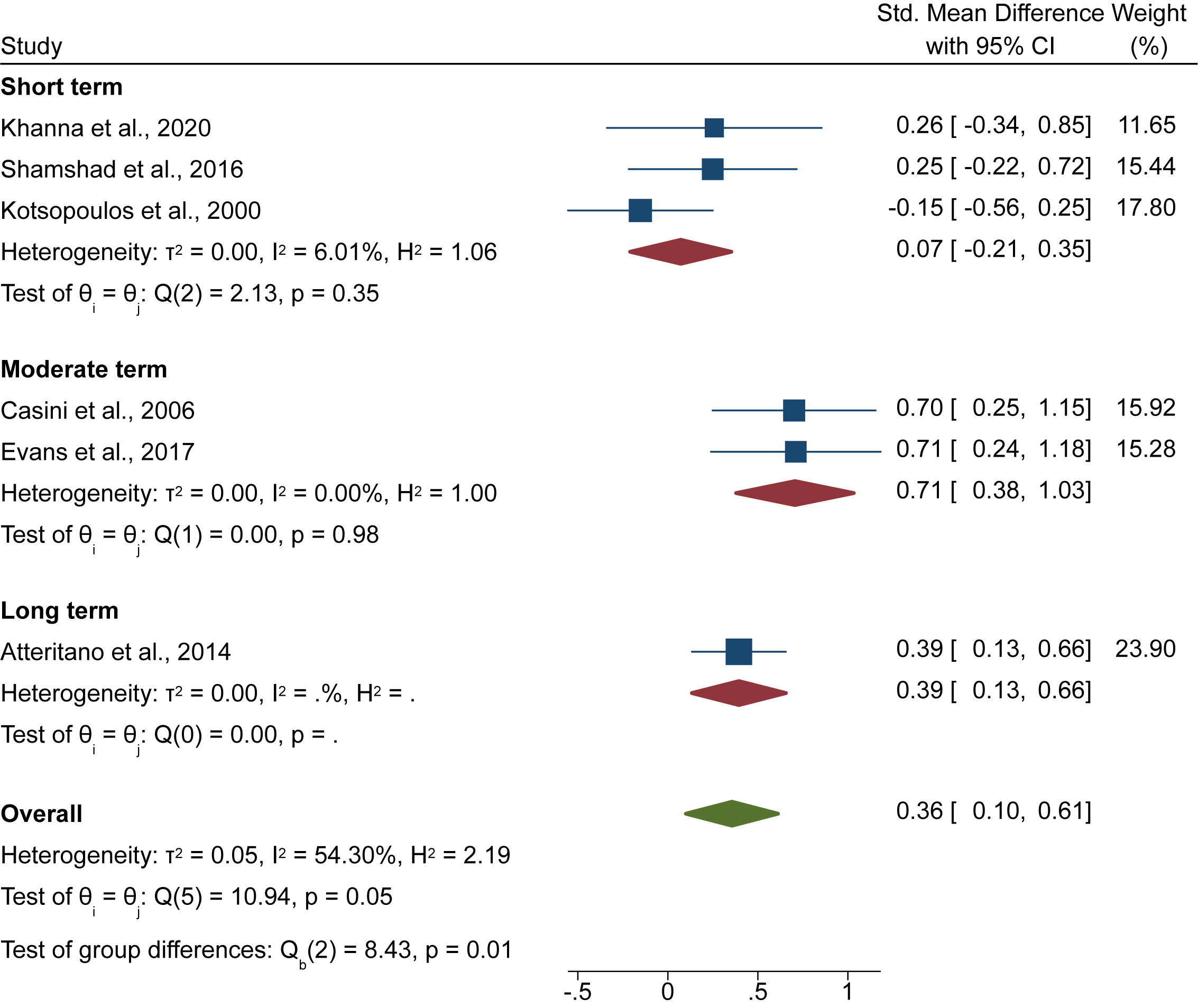


**eFigure 10. Outcomes of phytoestrogens for mood**

**
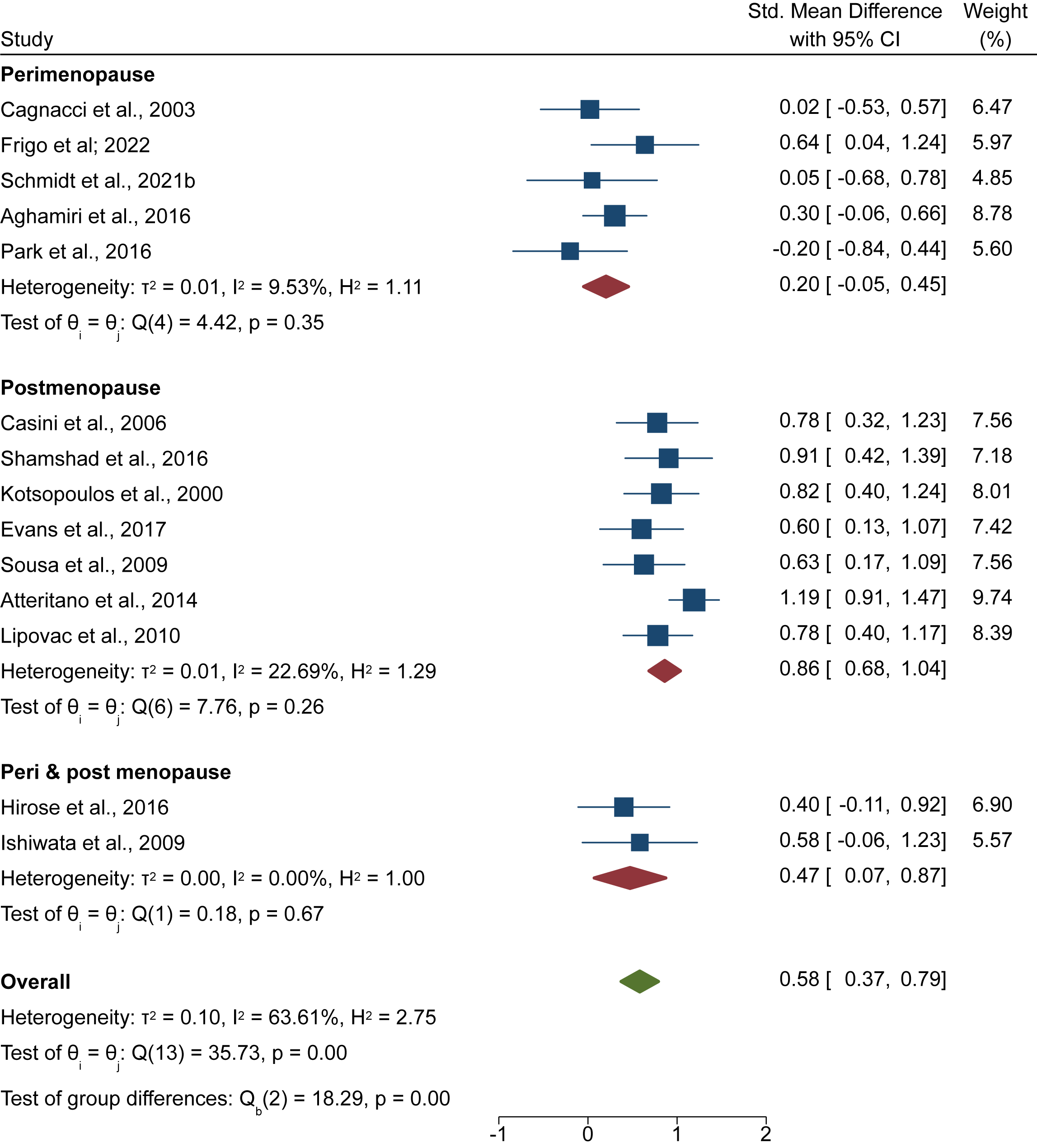
eFigure 11. Outcomes of phytoestrogens for depression**

**
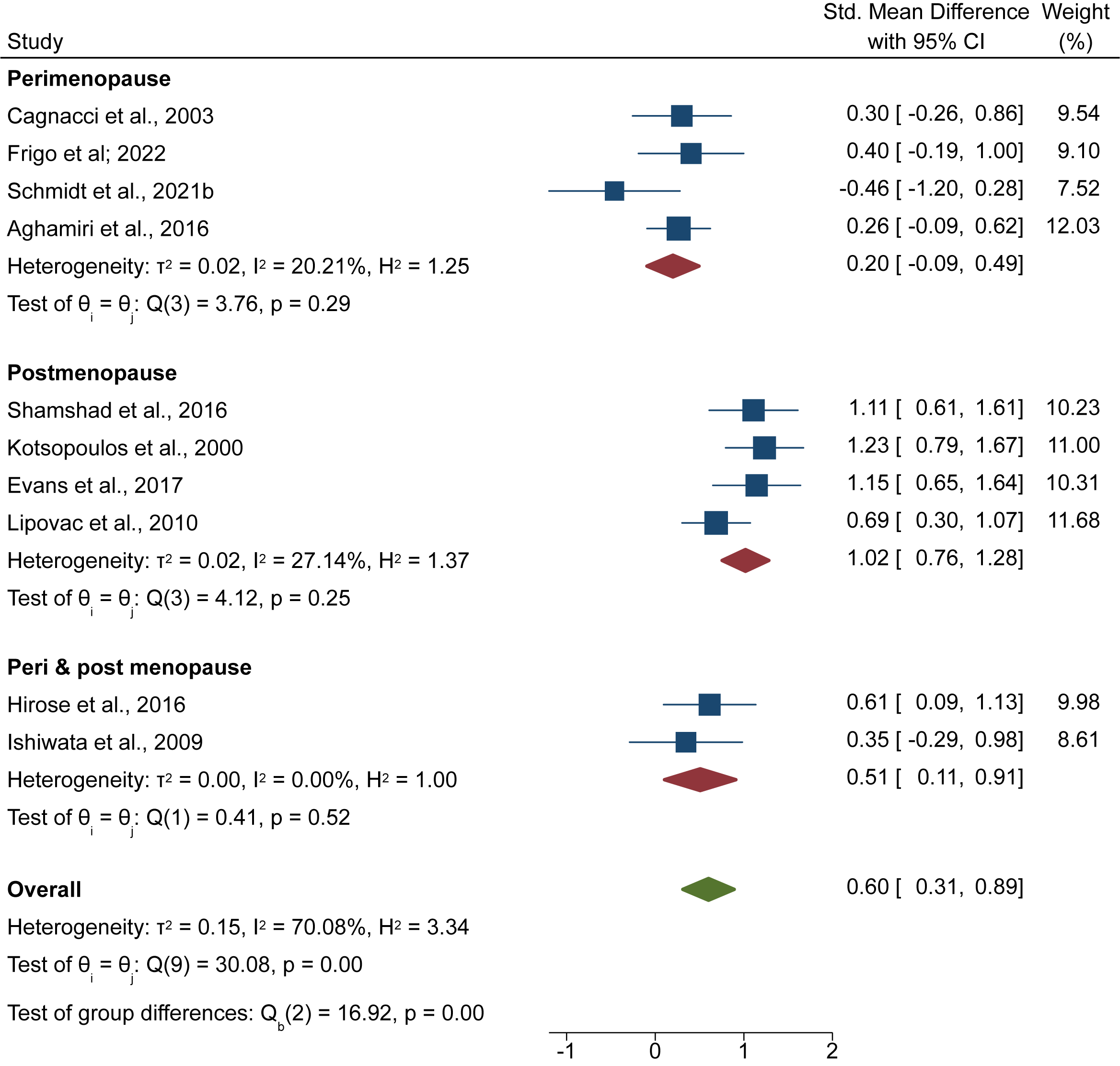
eFigure 12. Outcomes of phytoestrogens for anxiety**

**
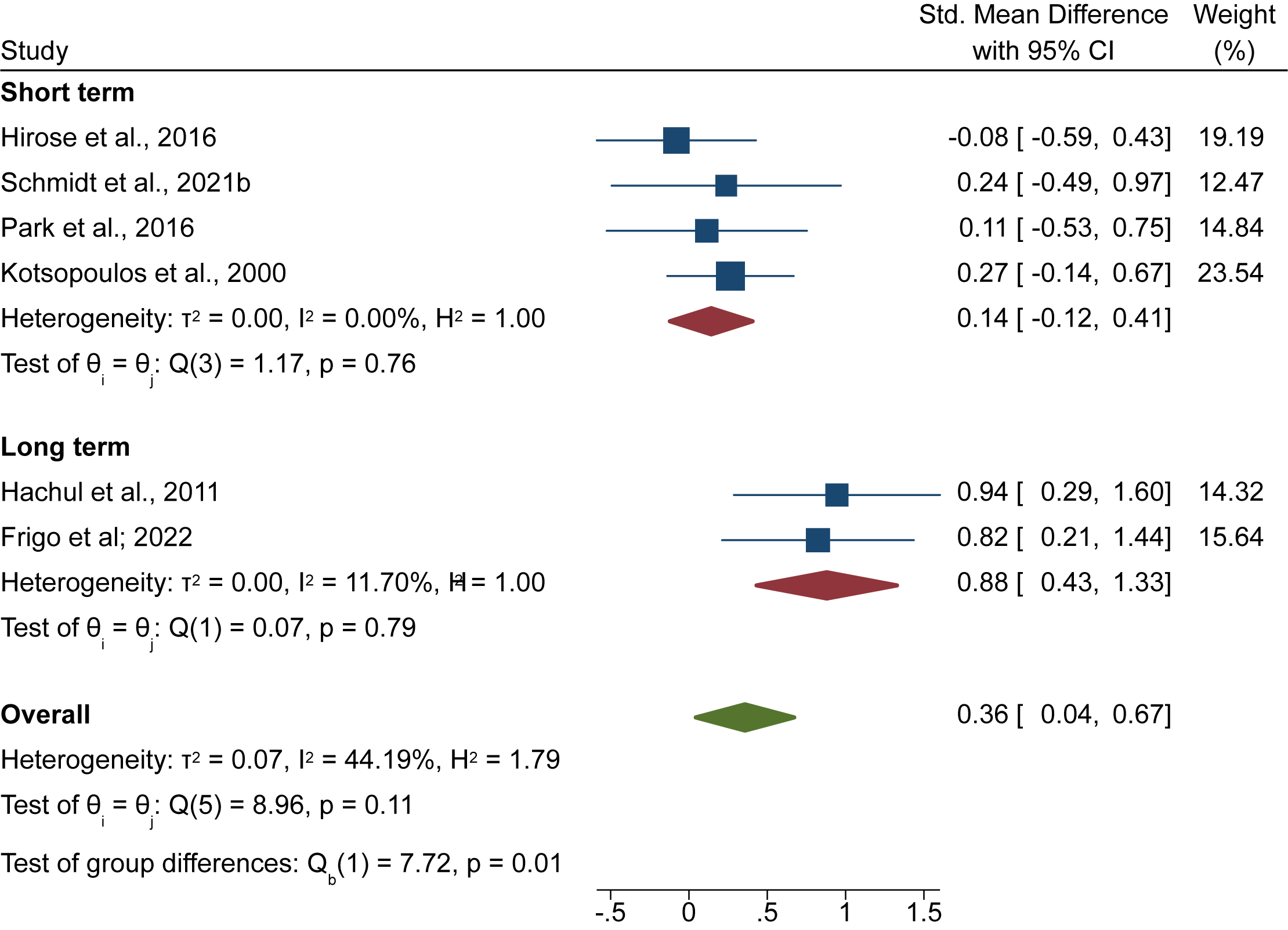
eFigure 13. Outcomes of phytoestrogens for sleep**

**
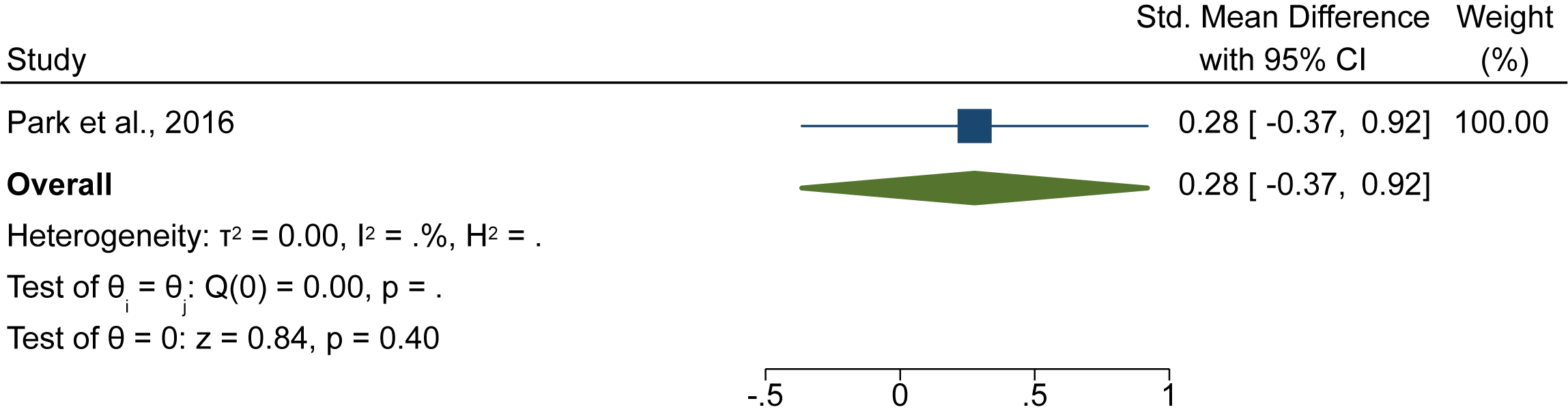
eFigure 14. Outcomes of phytoestrogens for stress**

**
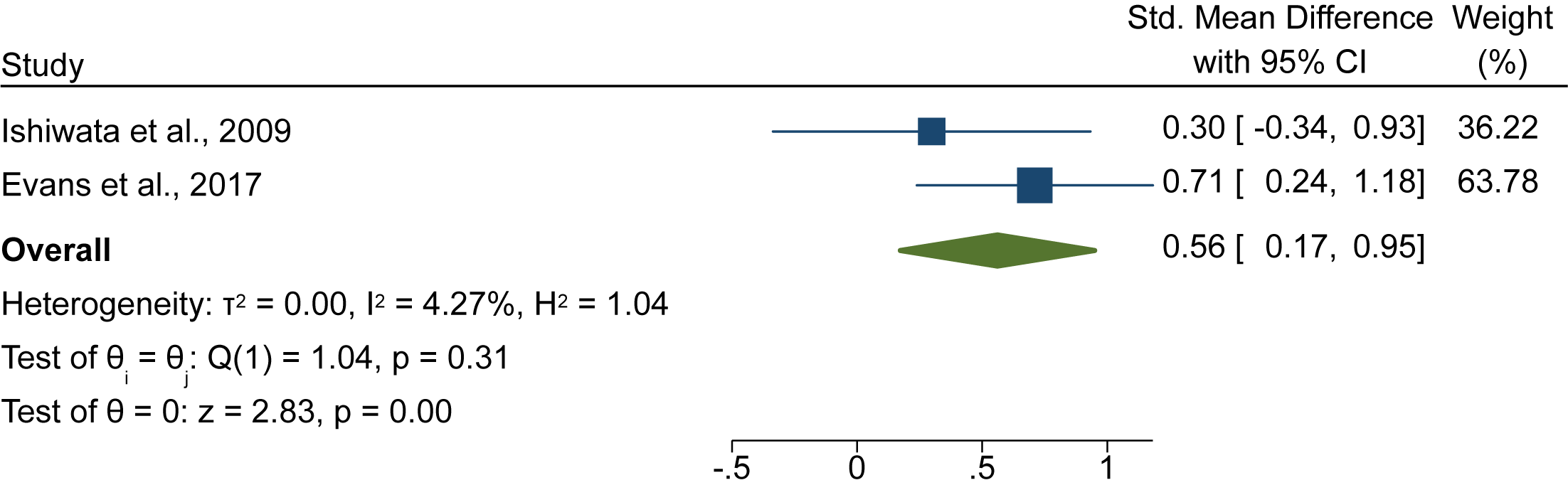
eFigure 15. Outcomes of phytoestrogens for anger**

**
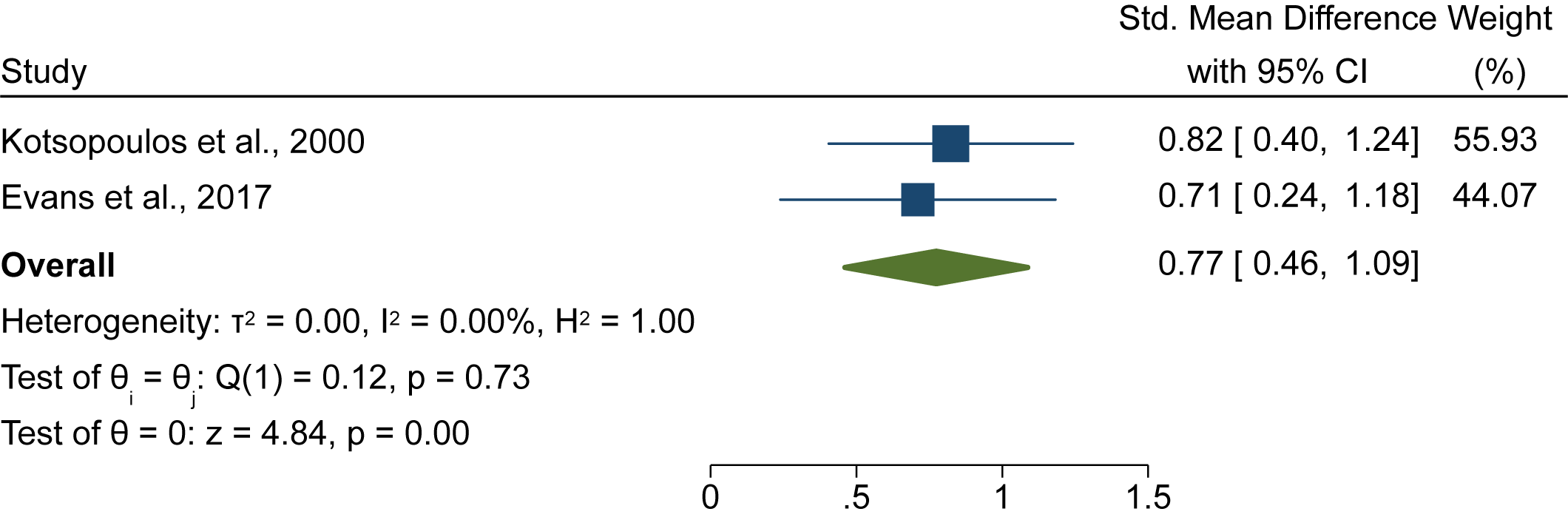
eFigure 16. Outcomes of phytoestrogens for phobic**

**
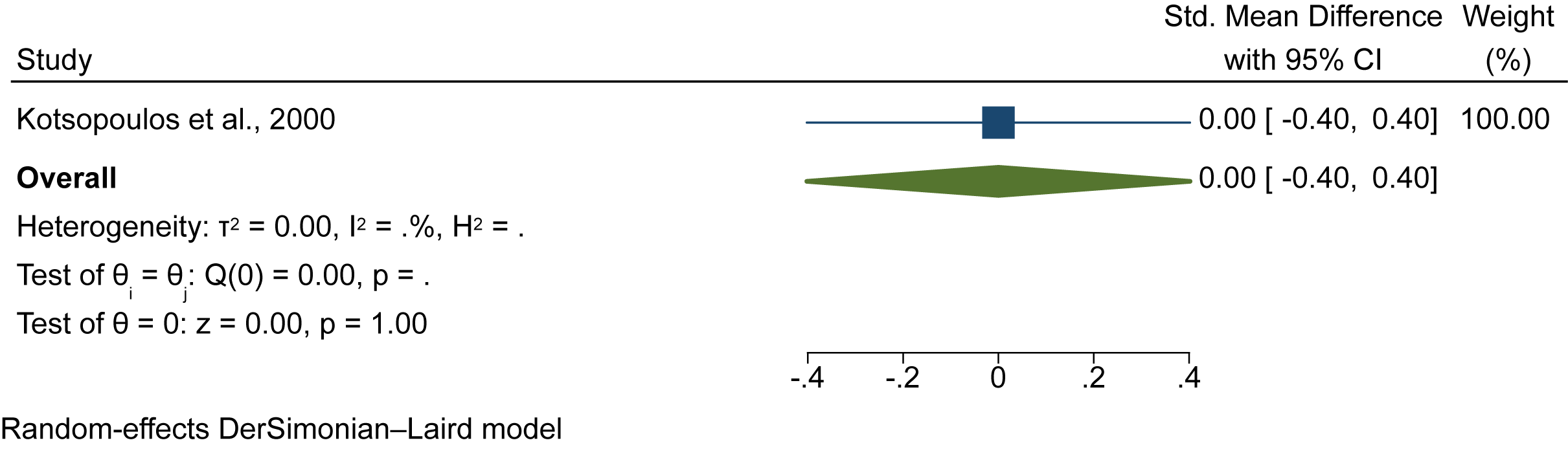
eFigure 17. Outcomes of phytoestrogens for well-being**

### eTable 7. Meta-regression summary of hormone therapy

| **Symptoms** | **Variables** | **Beta (95% CI)** | **SE** | ***P***.value | **Sig** |
| --- | --- | --- | --- | --- | --- |
| Mood |  |  |  |  |  |
|  | Duration: Moderate term | -0.337 (-0.568 to -0.106) | 0.118 | 0.004 | ** |
|  | Duration: Short term | -0.859 (-1.322 to -0.396) | 0.236 | <0.001 | *** |
|  | Participants: Perimenopause | -0.613 (-1.572 to 0.345) | 0.489 | 0.210 |  |
|  | Participants: Postmenopause | -0.523 (-1.391 to 0.345) | 0.443 | 0.238 |  |
|  | Interventions: PET | 0.000 (-0.221 to 0.221) | 0.113 | 0.997 |  |
|  | Interventions: Tibolone | 0.218 (-0.264 to 0.700) | 0.246 | 0.376 |  |
|  | Route: Transdermal | 0.071 (-0.271 to 0.413) | 0.175 | 0.683 |  |
|  | Route: Vaginal | 0.063 (-0.511 to 0.637) | 0.293 | 0.829 |  |
|  | Regions: Europe | 0.500 (-0.299 to 1.300) | 0.408 | 0.220 |  |
|  | Regions: Multinational | 0.026 (-0.690 to 0.742) | 0.365 | 0.944 |  |
|  | Regions: USA | 0.315 (-0.484 to 1.115) | 0.408 | 0.439 |  |
| Depression |  |  |  |  |  |
|  | Duration: Moderate term | 0.116 (-0.038 to 0.193) | 0.139 | 0.103 |  |
|  | Duration: Short term | 0.175 (-0.028 to 0.323) | 0.275 | 0.202 |  |
|  | Participants: Perimenopause | -0.211 (-0.794 to 0.372) | 0.297 | 0.478 |  |
|  | Participants: Postmenopause | -0.333 (-0.889 to 0.223) | 0.284 | 0.240 |  |
|  | Participants: Surgical menopause | 0.151 (-0.402 to 0.704) | 0.282 | 0.592 |  |
|  | Interventions: PET | -0.272 (-0.351 to -0.192) | 0.041 | <0.001 | *** |
|  | Interventions: Tibolone | 0.248 (-0.137 to 0.634) | 0.197 | 0.206 |  |
|  | Route: Oral | 0.090 (-0.513 to 0.694) | 0.308 | 0.770 |  |
|  | Route: Transdermal | 0.034 (-0.568 to 0.636) | 0.307 | 0.911 |  |
|  | Route: Vaginal | -0.264 (-0.936 to 0.409) | 0.343 | 0.442 |  |
|  | Regions: Australia | 0.065 (-0.373 to 0.504) | 0.224 | 0.771 |  |
|  | Regions: Europe | 0.518 (-0.158 to 0.878) | 0.184 | 0.205 |  |
|  | Regions: USA | 0.526 (-0.176 to 0.876) | 0.179 | 0.303 |  |
| Anxiety |  |  |  |  |  |
|  | Duration: Moderate term | 0.030 (-0.899 to 0.958) | 0.474 | 0.950 |  |
|  | Duration: Short term | 0.264 (-0.608 to 1.135) | 0.444 | 0.553 |  |
|  | Participants: Perimenopause | 0.504 (-0.813 to 1.82) | 0.672 | 0.453 |  |
|  | Participants: Postmenopause | 0.331 (-0.808 to 1.471) | 0.581 | 0.569 |  |
|  | Participants: Surgical menopause | 0.163 (-0.614 to 0.94) | 0.396 | 0.681 |  |
|  | Interventions: PET | 0.042 (-0.466 to 0.549) | 0.259 | 0.872 |  |
|  | Interventions: Tibolone | 0.319 (-0.312 to 0.951) | 0.322 | 0.022 |  |
|  | Route: Oral | 0.520 (0.221 to 1.261) | 0.378 | 0.029 | * |
|  | Route: Transdermal | 0.000 (-0.741 to 0.742) | 0.378 | 0.999 |  |
|  | Route: Vaginal | 0.715 (0.176 to 1.607) | 0.455 | 0.016 | * |
|  | Regions: Australia | -0.320 (-1.383 to 0.744) | 0.542 | 0.556 |  |
|  | Regions: Europe | 0.015 (-1.101 to 1.131) | 0.569 | 0.979 |  |
|  | Regions: Multinational | -0.194 (-1.413 to 1.025) | 0.622 | 0.755 |  |
|  | Regions: USA | -0.025 (-1.045 to 0.996) | 0.521 | 0.962 |  |

**Continued**

| **Symptoms** | **Variables** | **Beta (95% CI)** | **SE** | ***P***.value | **Sig** |
| --- | --- | --- | --- | --- | --- |
| Sleep quality |  |  |  |  |  |
|  | Duration: Moderate term | 0.034 (-0.341 to 0.410) | 0.192 | 0.858 |  |
|  | Duration: Short term | -0.010 (-0.278 to 0.258) | 0.137 | 0.943 |  |
|  | Participants: Perimenopause | -0.200 (-0.613 to 0.214) | 0.211 | 0.344 |  |
|  | Participants: Postmenopause | 0.521 (0.170 to 0.871) | 0.179 | 0.004 | ** |
|  | Interventions: PET | -0.126 (-0.500 to 0.248) | 0.191 | 0.509 |  |
|  | Interventions: Progesterone | -0.013 (-0.708 to 0.683) | 0.355 | 0.972 |  |
|  | Interventions: Tibolone | -0.064 (-0.664 to 0.535) | 0.306 | 0.833 |  |
|  | Route: Oral | 0.125 (-0.162 to 0.411) | 0.146 | 0.394 |  |
|  | Route: Transdermal | 0.122 (-0.236 to 0.481) | 0.183 | 0.504 |  |
|  | Regions: Europe | -0.068 (-0.471 to 0.335) | 0.206 | 0.742 |  |
|  | Regions: Multinational | -0.108 (-0.843 to 0.626) | 0.375 | 0.773 |  |
|  | Regions: USA | -0.058 (-0.456 to 0.34) | 0.203 | 0.774 |  |
| Anger |  |  |  |  |  |
|  | Interventions: PET | 0.854 (0.033 to 1.675) | 0.419 | 0.042 | * |
| QOL |  |  |  |  |  |
|  | Duration: Short term | 0.338 (-0.253 to 0.929) | 0.302 | 0.262 |  |
|  | Participants: Postmenopause | 0.496 (-0.094 to 1.086) | 0.301 | 0.099 |  |
|  | Regions: Australia | 0.476 (0.272 to 1.223) | 0.381 | 0.002 | ** |

### eTable 8. Meta-regression summary of phytoestrogens

| **Symptoms** | **Variables** | **Beta (95% CI)** | **SE** | ***P*.value** | **Sig** |
| --- | --- | --- | --- | --- | --- |
| Mood |  |  |  |  |  |
|  | Duration: Moderate term | 0.307 (-0.221 to 0.834) | 0.269 | 0.254 |  |
|  | Duration: Short term | -0.557 (-1.376 to -0.262) | 0.418 | 0.003 | ** |
|  | Regions: Australia | -0.406 (-0.957 to 0.146) | 0.281 | 0.149 |  |
|  | Regions: Europe | -0.415 (-1.276 to 0.446) | 0.439 | 0.345 |  |
| Depression |  |  |  |  |  |
|  | Duration: Moderate term | -0.444 (-0.842 to -0.046) | 0.203 | 0.009 | ** |
|  | Duration: Short term | -0.480 (-1.039 to 0.079) | 0.285 | 0.092 |  |
|  | Participants: Perimenopause | -0.243 (-0.745 to 0.259) | 0.256 | 0.343 |  |
|  | Participants: Postmenopause | 0.328 (-0.241 to 0.897) | 0.290 | 0.259 |  |
|  | Regions: Australia | -0.090 (-0.579 to 0.399) | 0.250 | 0.718 |  |
|  | Regions: Europe | -0.088 (-0.574 to 0.398) | 0.248 | 0.723 |  |
|  | Regions: USA | -0.033 (-0.571 to 0.504) | 0.274 | 0.904 |  |
| Anxiety |  |  |  |  |  |
|  | Duration: Short term | -0.081 (-0.645 to 0.482) | 0.288 | 0.777 |  |
|  | Participants: Perimenopause | -0.138 (-0.792 to -0.516) | 0.334 | 0.019 | * |
|  | Participants: Postmenopause | 0.455 (0.260 to 1.169) | 0.364 | 0.002 | ** |
|  | Regions: Australia | 0.199 (-0.461 to 0.859) | 0.337 | 0.555 |  |
|  | Regions: Europe | -0.242 (-0.879 to 0.395) | 0.325 | 0.457 |  |
|  | Regions: USA | -0.36 (-1.175 to 0.455) | 0.416 | 0.387 |  |
| Sleep quality |  |  |  |  |  |
|  | Duration: Short term | -0.237 (-1.323 to -0.243) | 0.450 | 0.014 | * |
|  | Regions: Australia | -0.642 (-1.523 to -0.240) | 0.293 | 0.355 |  |
|  | Regions: USA | 0.244 (-0.611 to 1.099) | 0.436 | 0.576 |  |

### eFigure 18-26. Bubble plots of hormone therapy and phytoestrogens

**
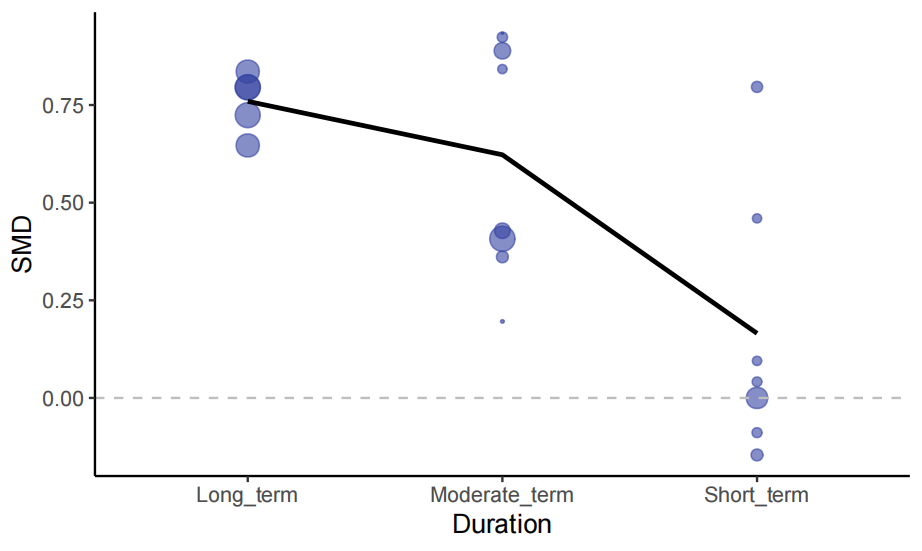
eFigure 18. Bubble plots of hormone therapy for mood**

**
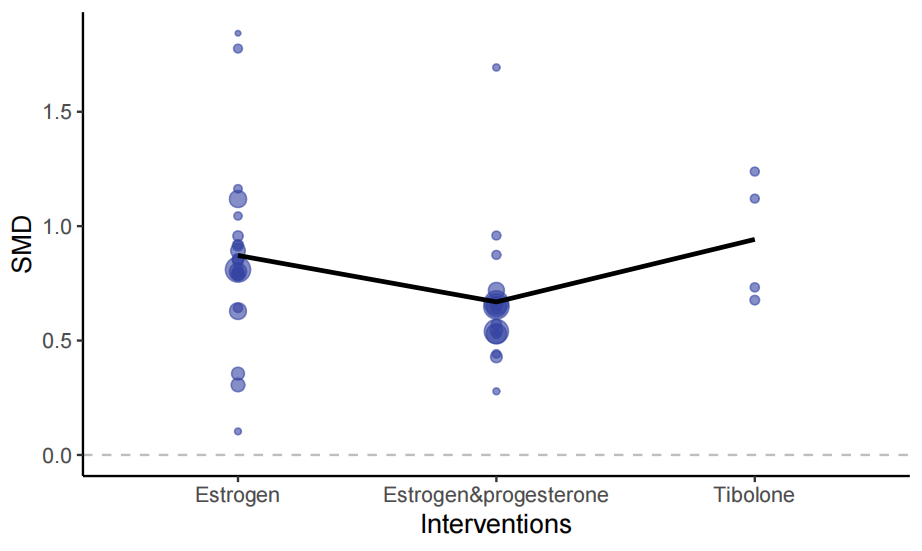
eFigure 19. Bubble plots of hormone therapy for depression**

**
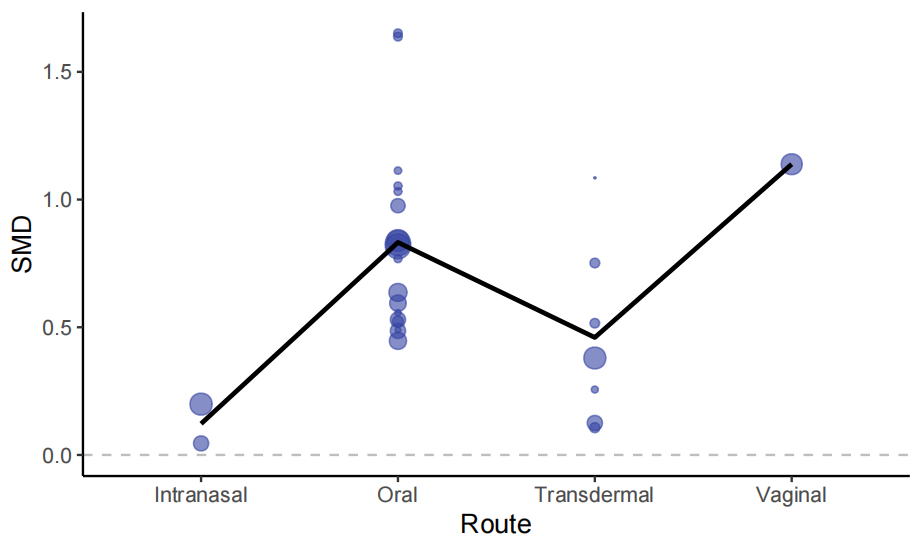
eFigure 20. Bubble plots of hormone therapy for anxiety**

**
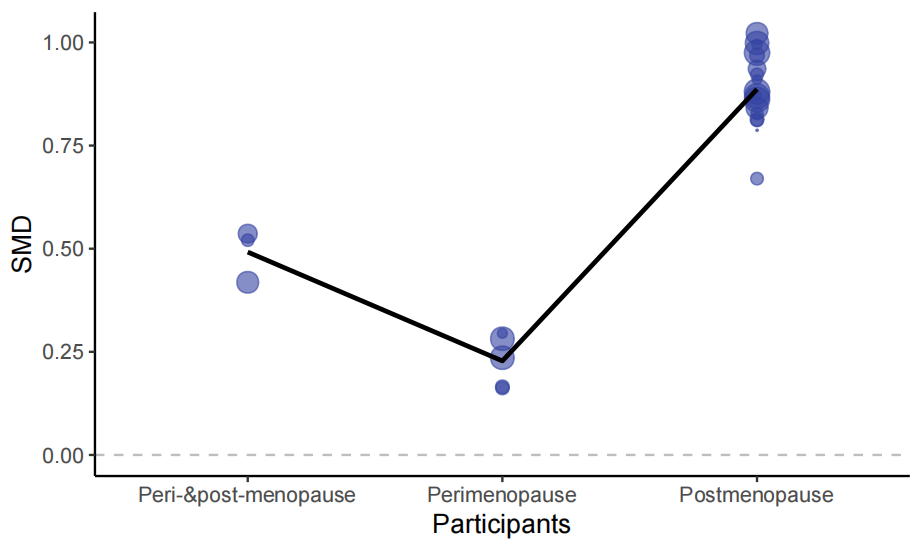
eFigure 21. Bubble plots of hormone therapy for sleep quality**

**
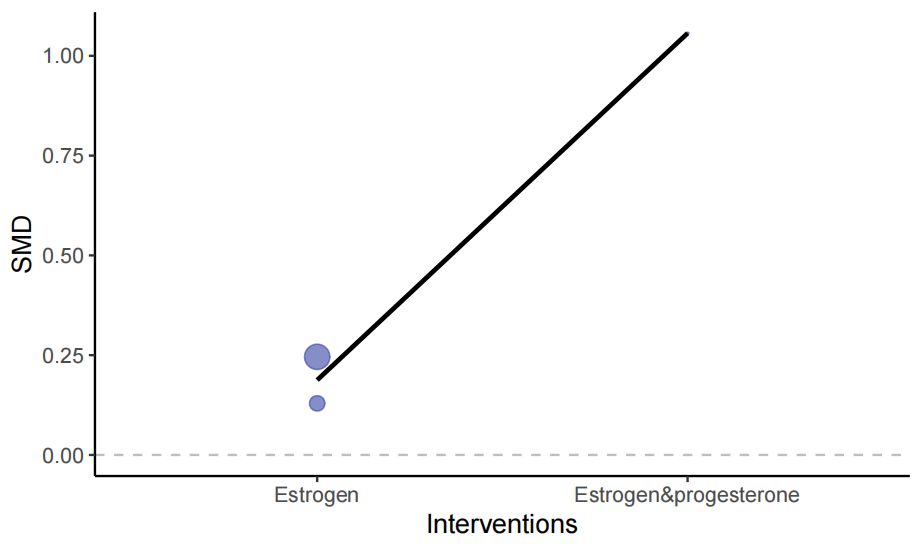
eFigure 22. Bubble plots of hormone therapy for anger**

**
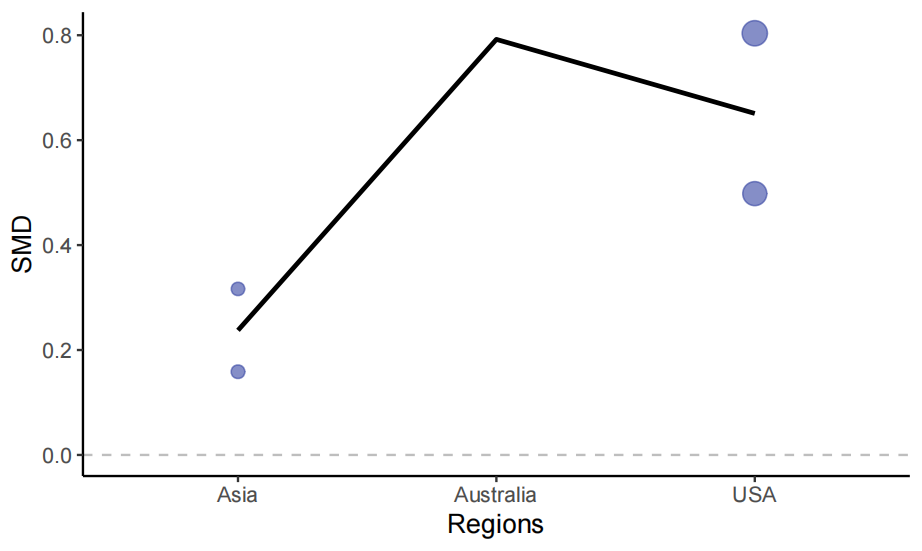
eFigure 23. Bubble plots of hormone therapy for QOL**

**
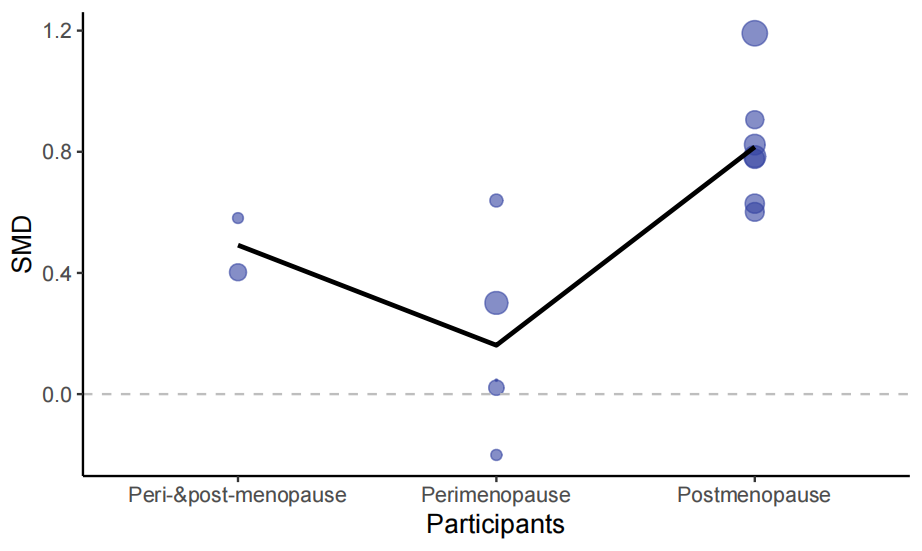

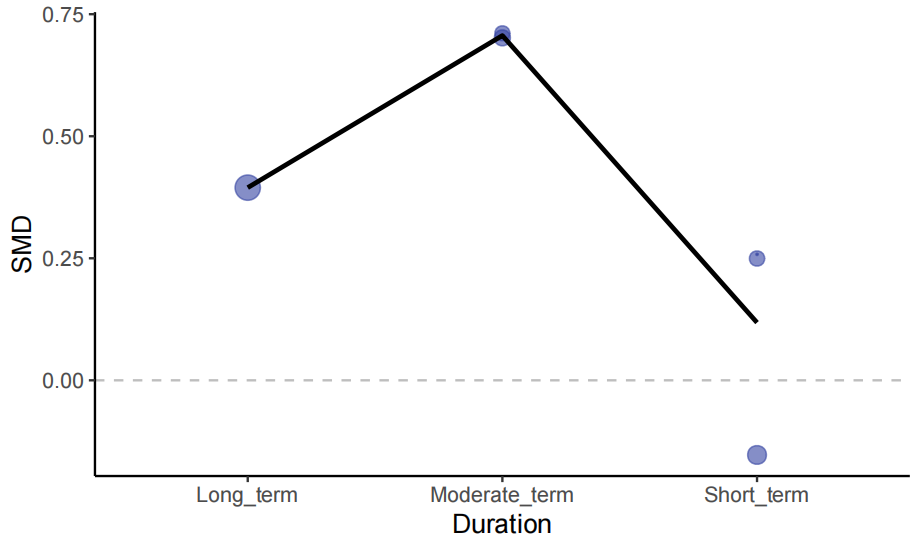
eFigure 24. Bubble plots of phytoestrogens for mood**

**eFigure 25. Bubble plots of phytoestrogens for depression**

**
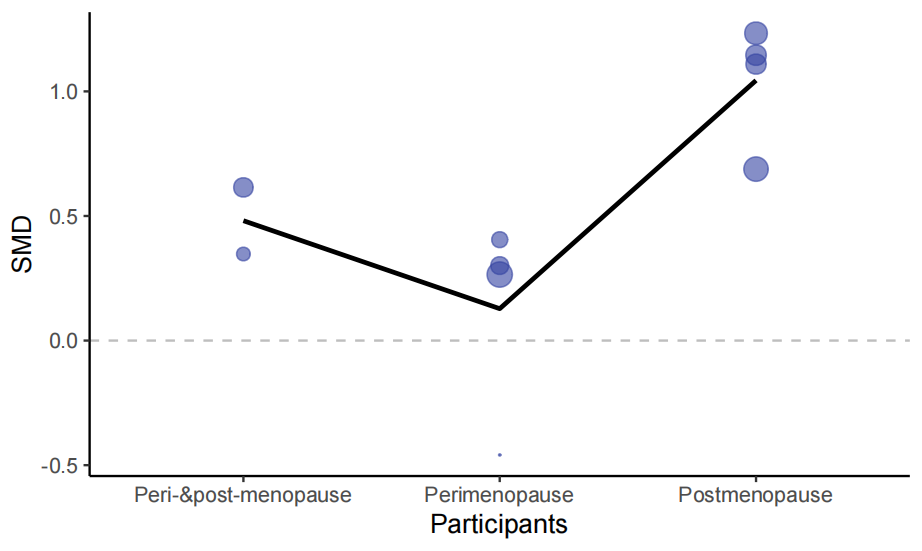
eFigure 26. Bubble plots of phytoestrogens for anxiety**

.

### eFigure 27-28. Forest plots of hormone therapy and phytoestrogens

**
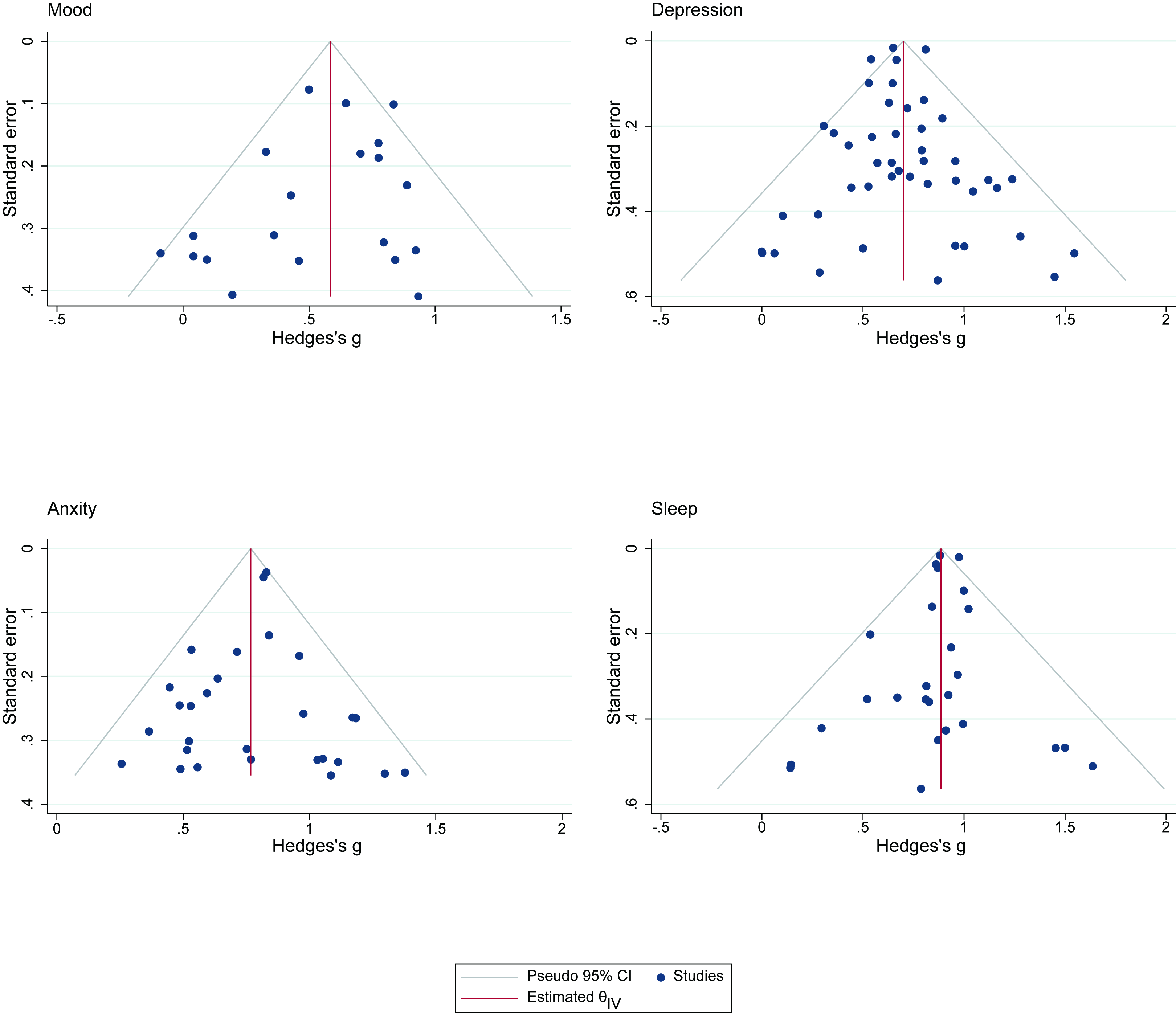
**

**eFigure 27.** **Forest plots of** **hormone therapy on menopausal psychological health**

**
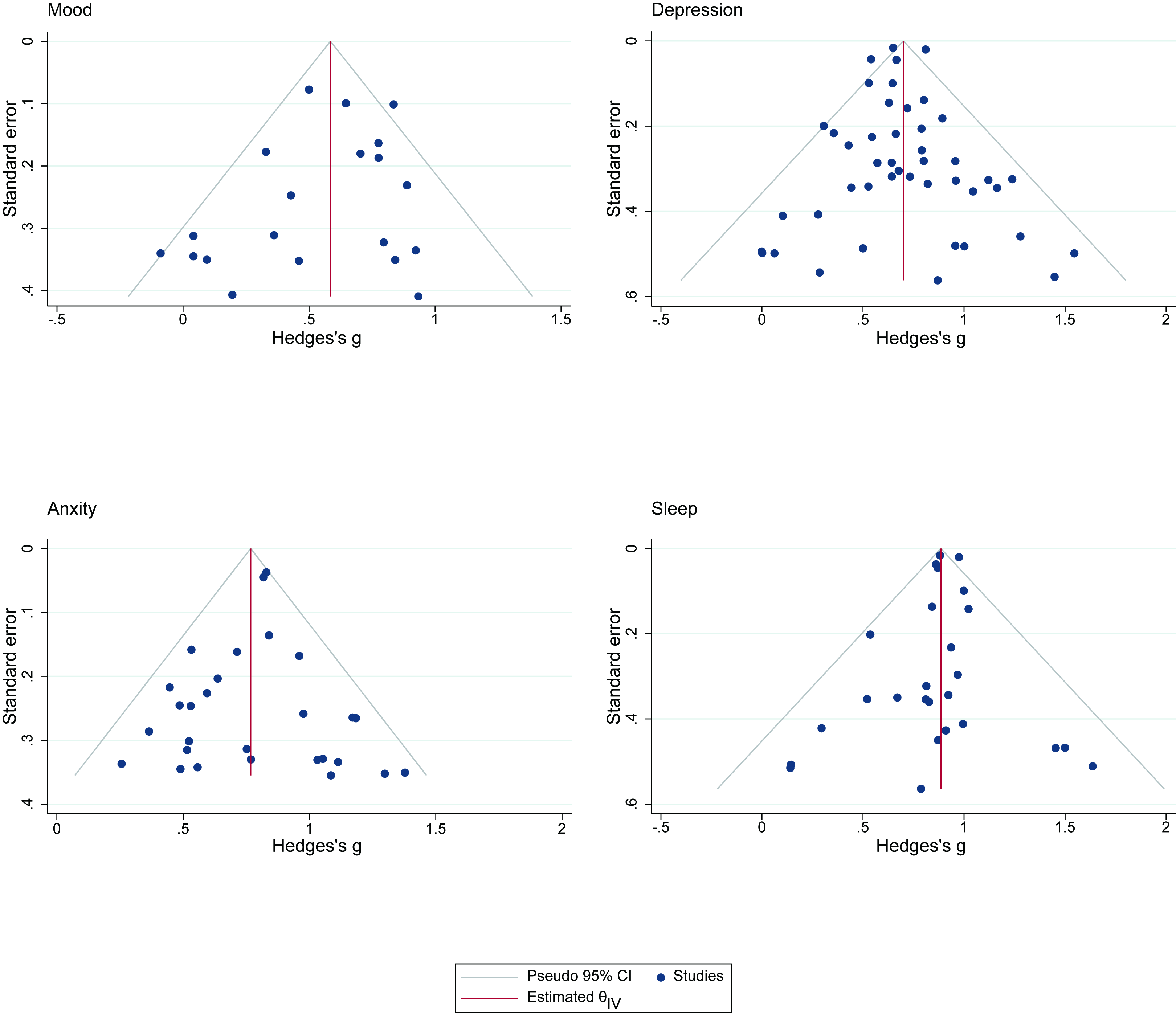
**

**eFigure 28. Forest plots of phytoestrogens on menopausal psychological health**

### eFigure 29-30. Galbraith plots of hormone therapy and phytoestrogens

**
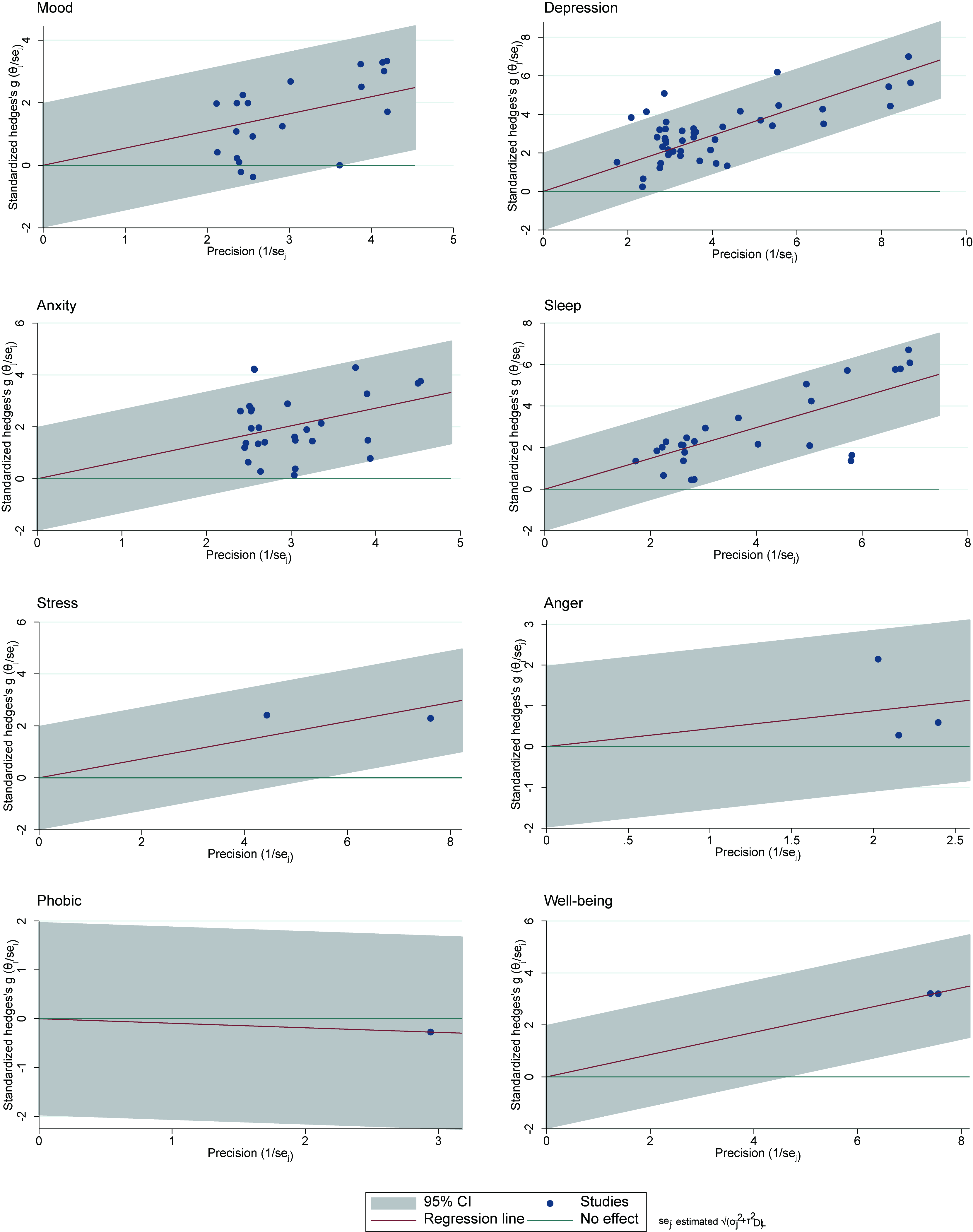
**

**eFigure 29. Galbraith plots of hormone therapy on menopausal psychological health**


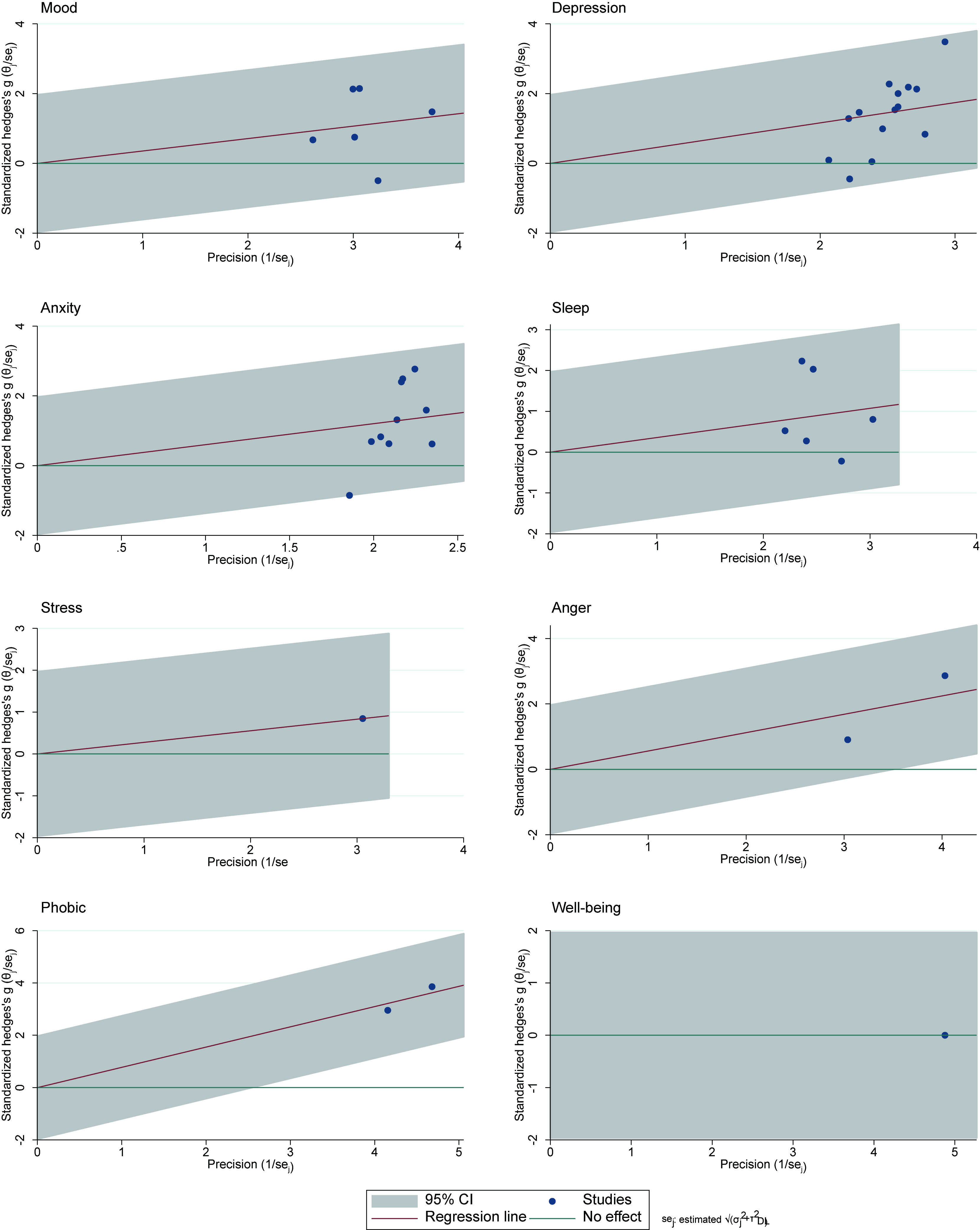


**eFigure 30. Galbraith plots of phytoestrogens on menopausal psychological health**

### eFigure 31-47. Trial sequential analysis of hormone therapy and phytoestrogens

**
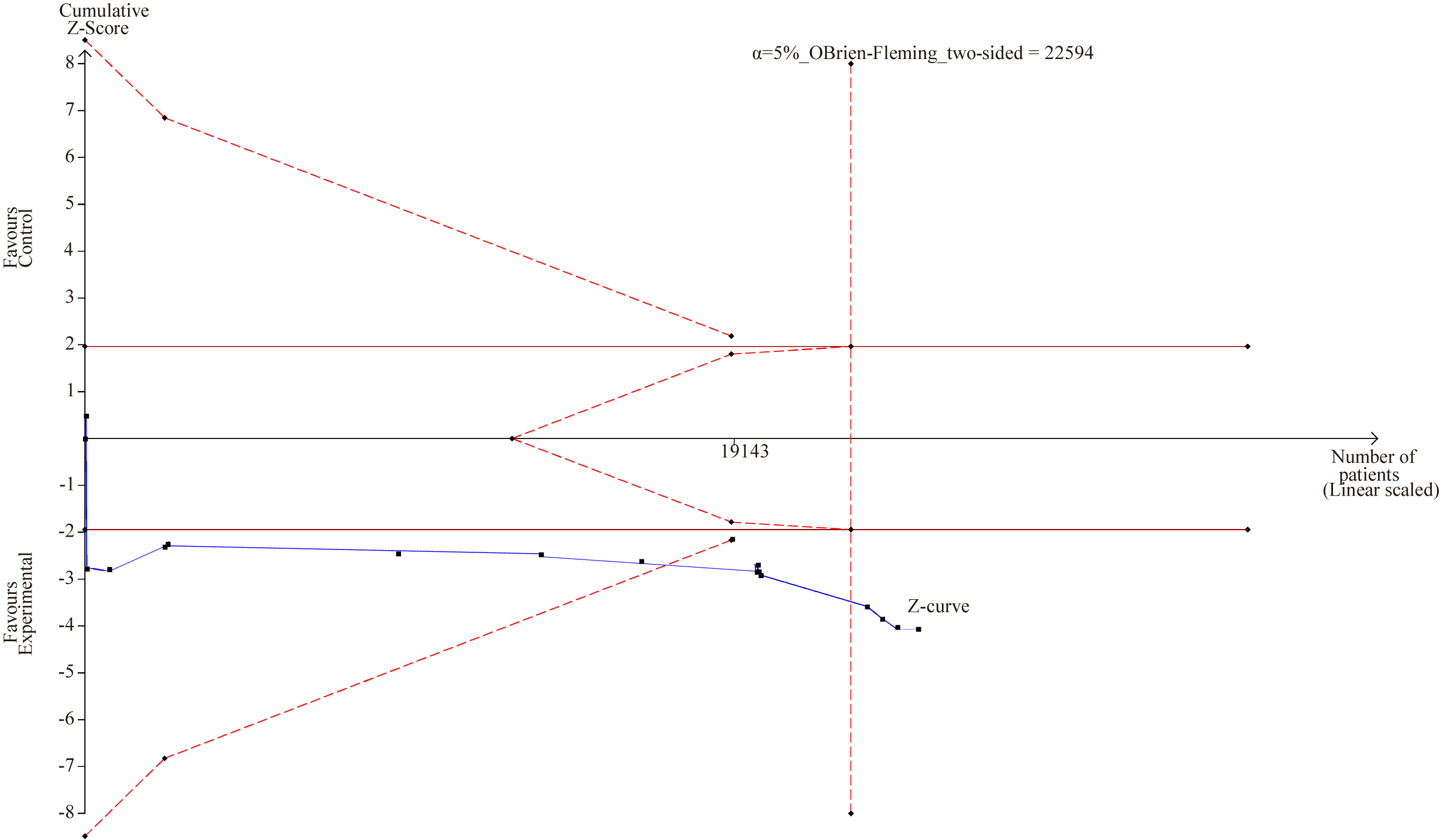
**

**eFigure 31. Trial sequential analysis of hormone therapy for mood**

**
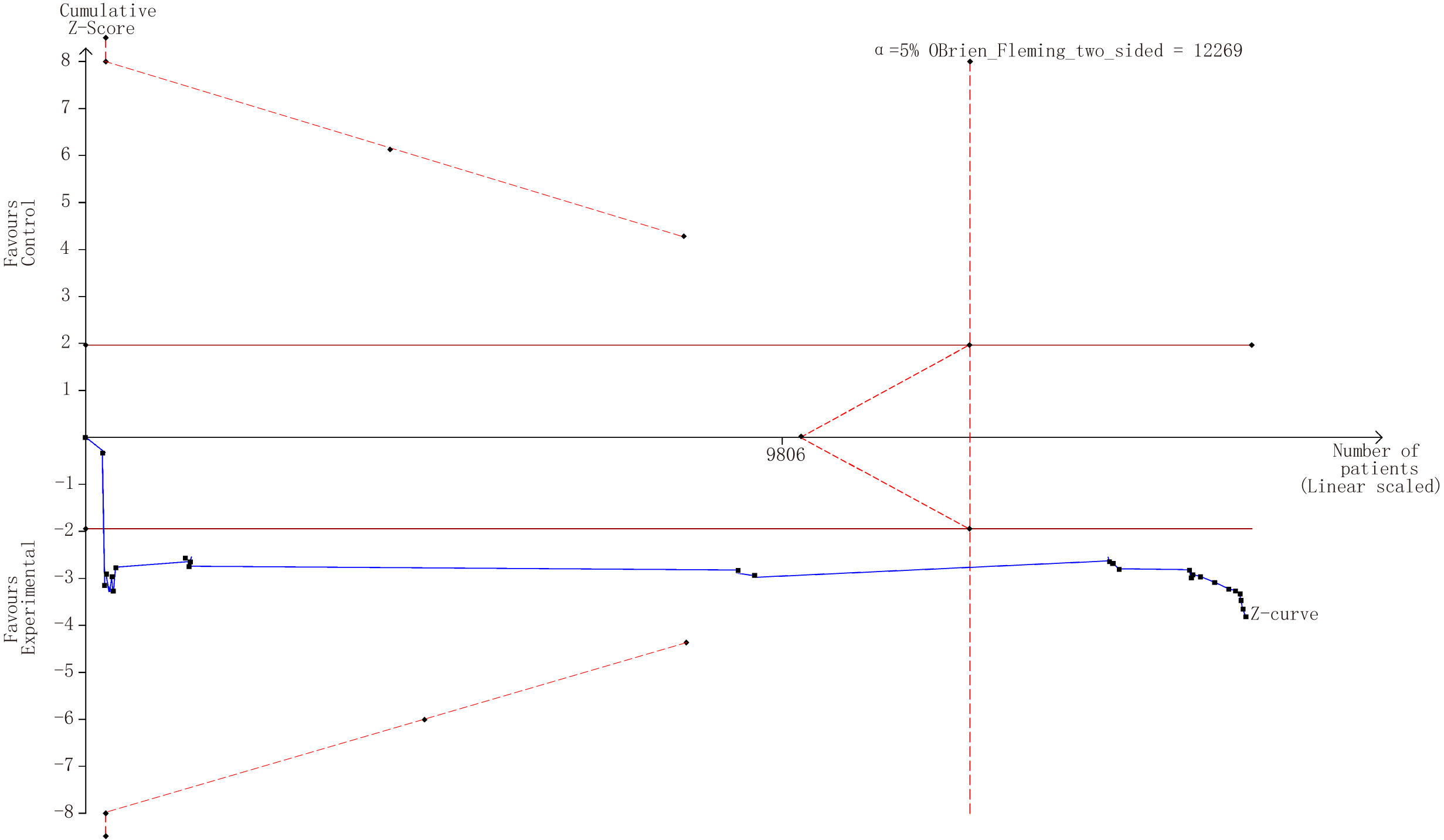
**

**eFigure 32. Trial sequential analysis of hormone therapy for depression**

**
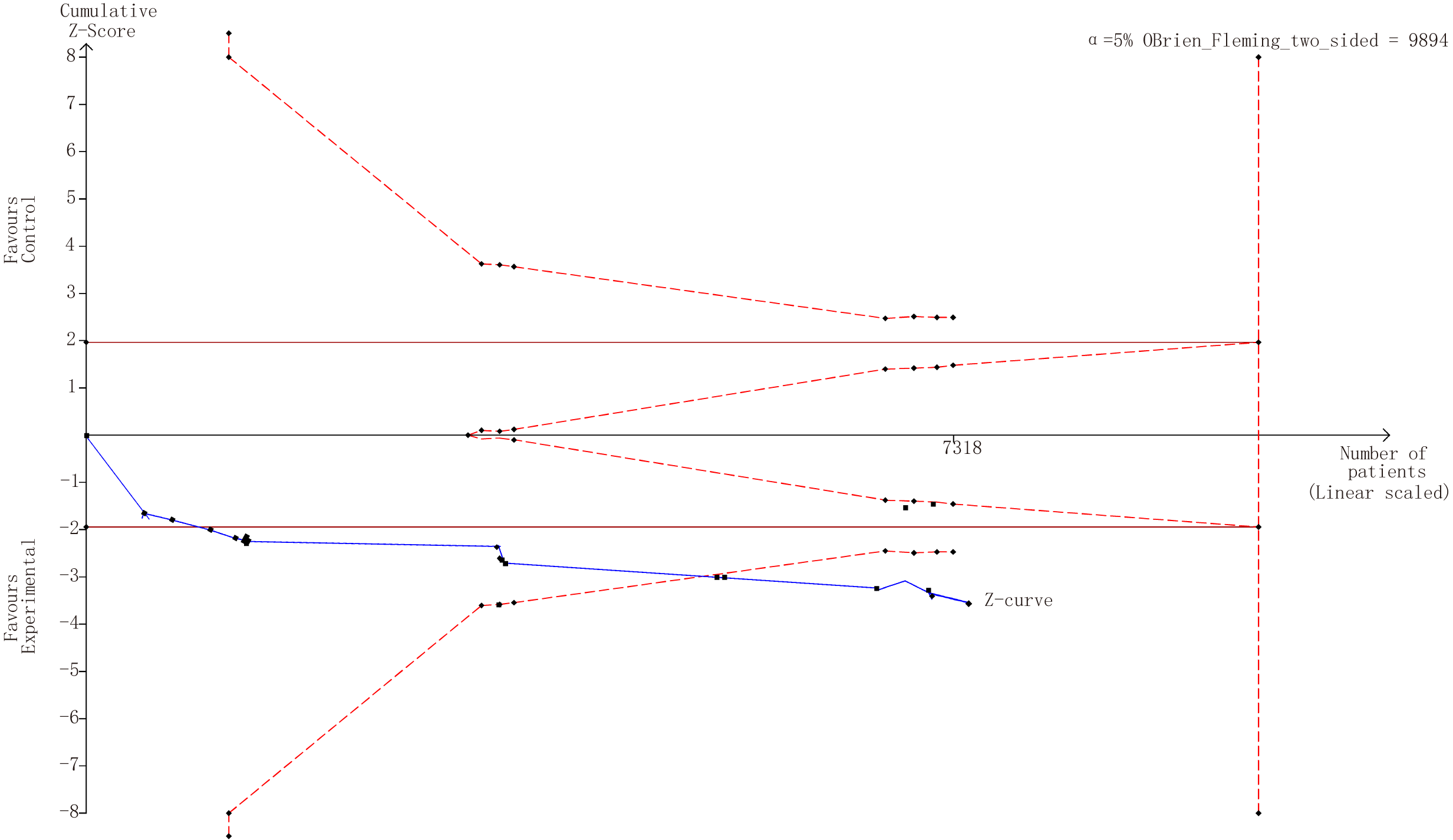
**

**eFigure 33. Trial sequential analysis of hormone therapy for anxiety**

**
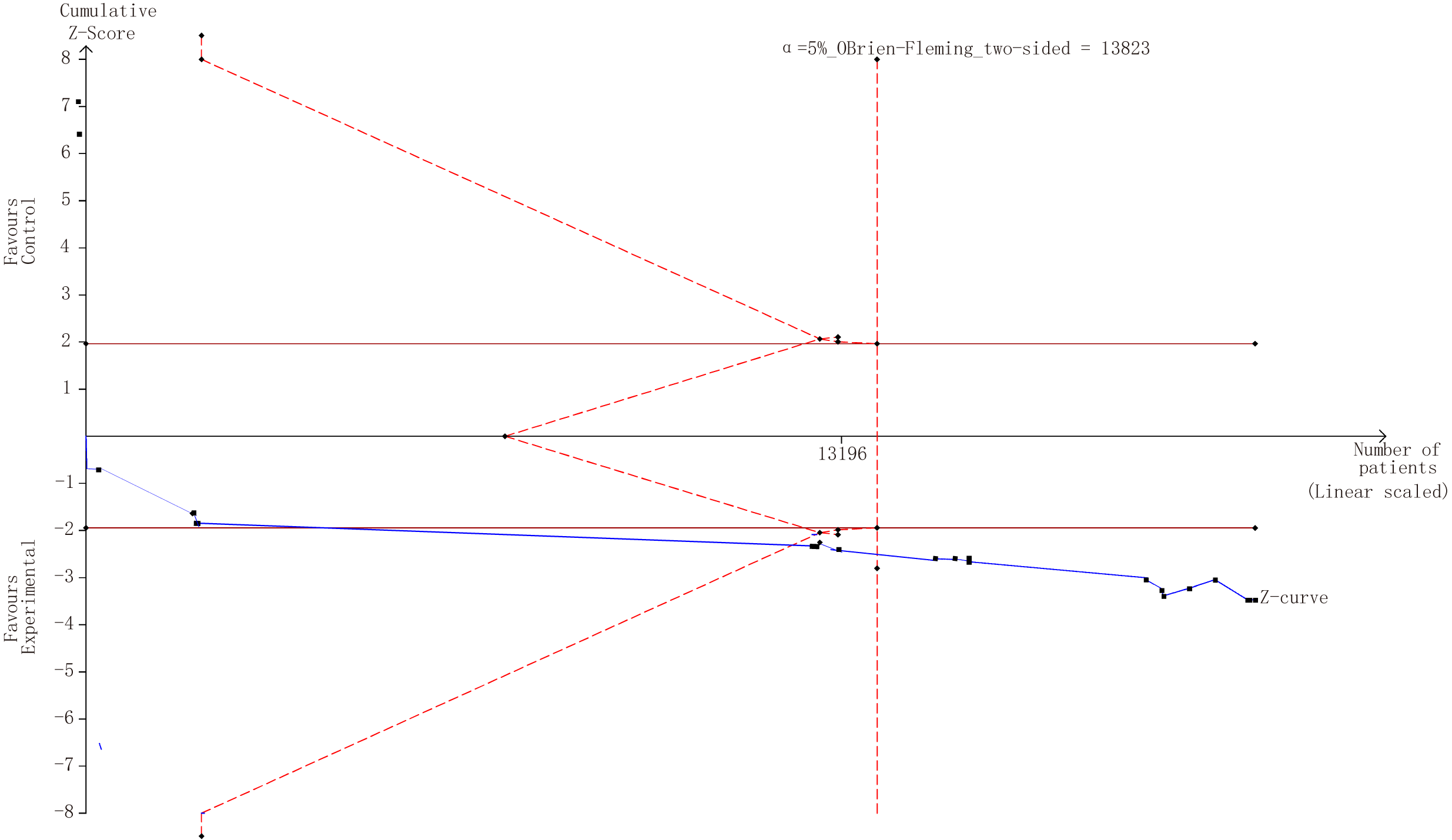
**

**eFigure 34. Trial sequential analysis of hormone therapy for sleep**

**
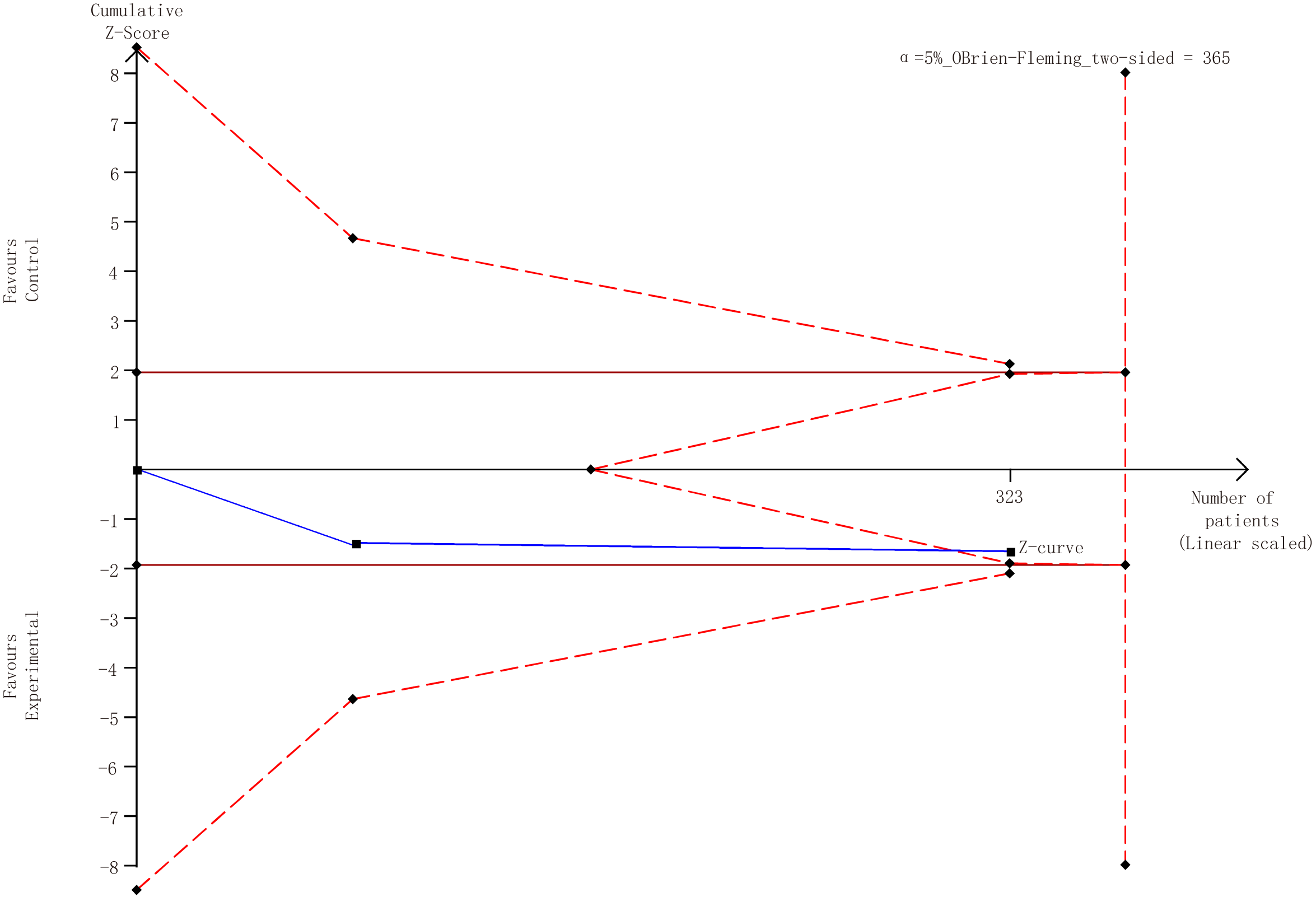
**

**eFigure 35. Trial sequential analysis of hormone therapy for stress**

**
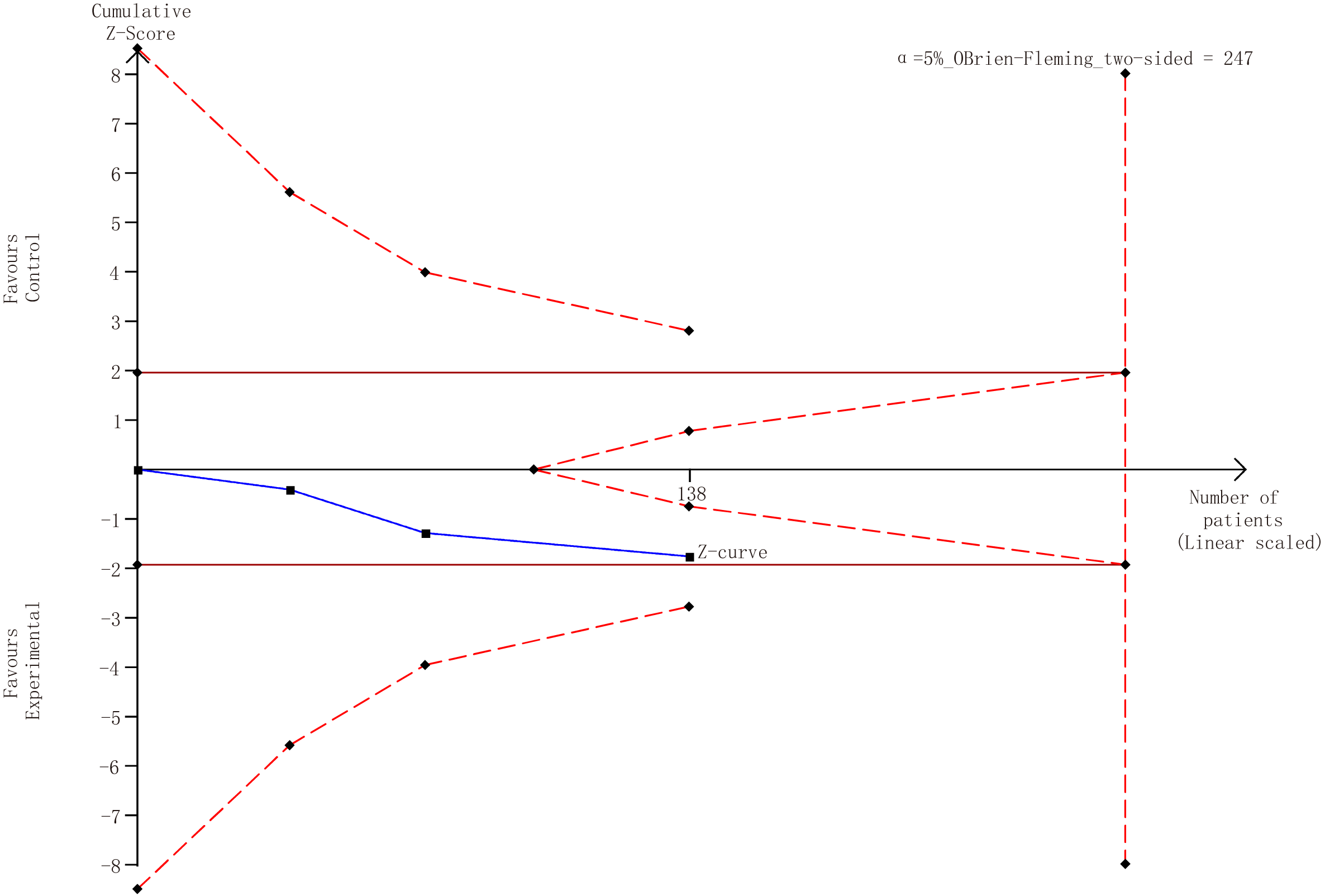
**

**eFigure 36. Trial sequential analysis of hormone therapy for anger**

**
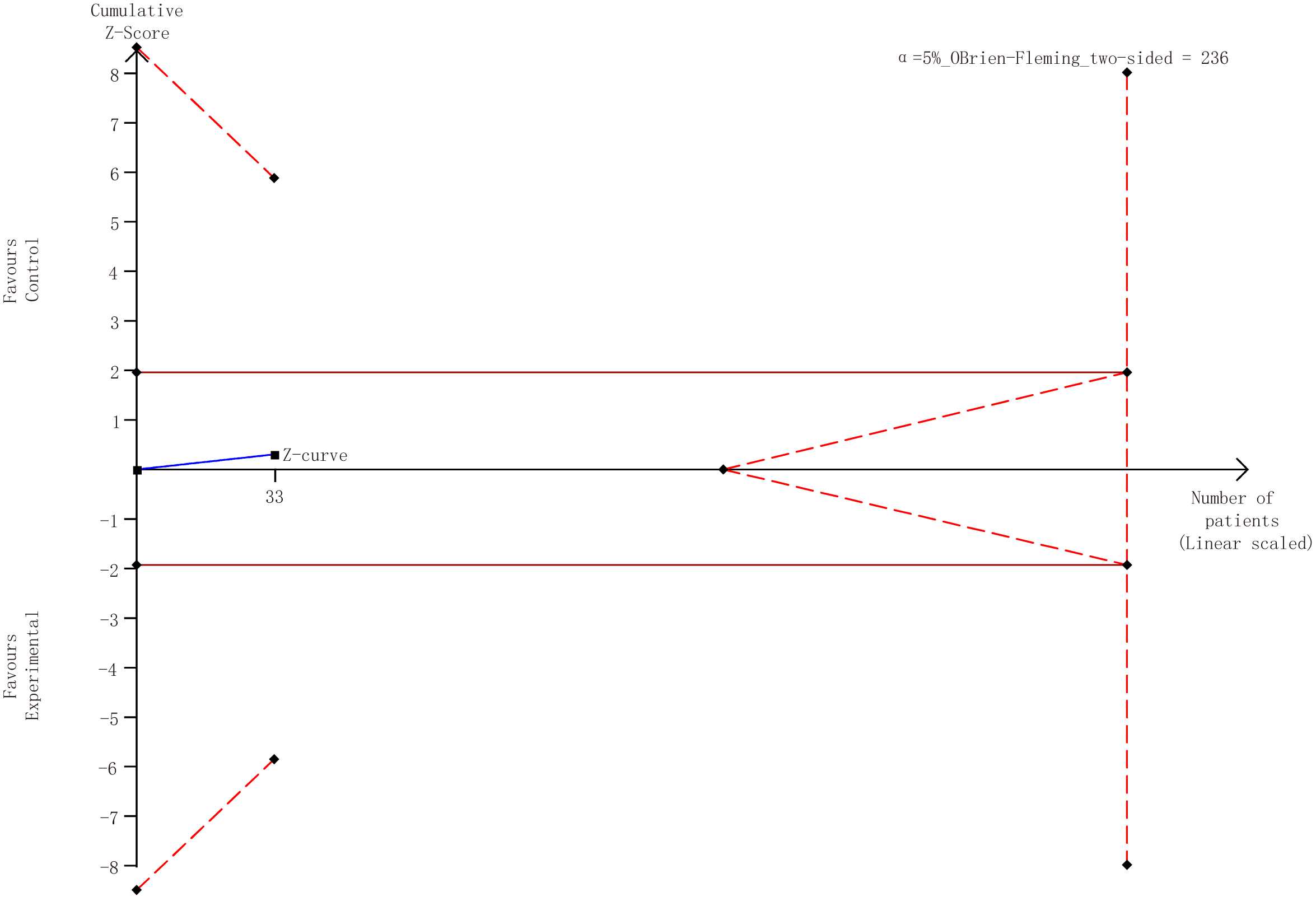
**

**eFigure 37. Trial sequential analysis of hormone therapy for phobic**

**
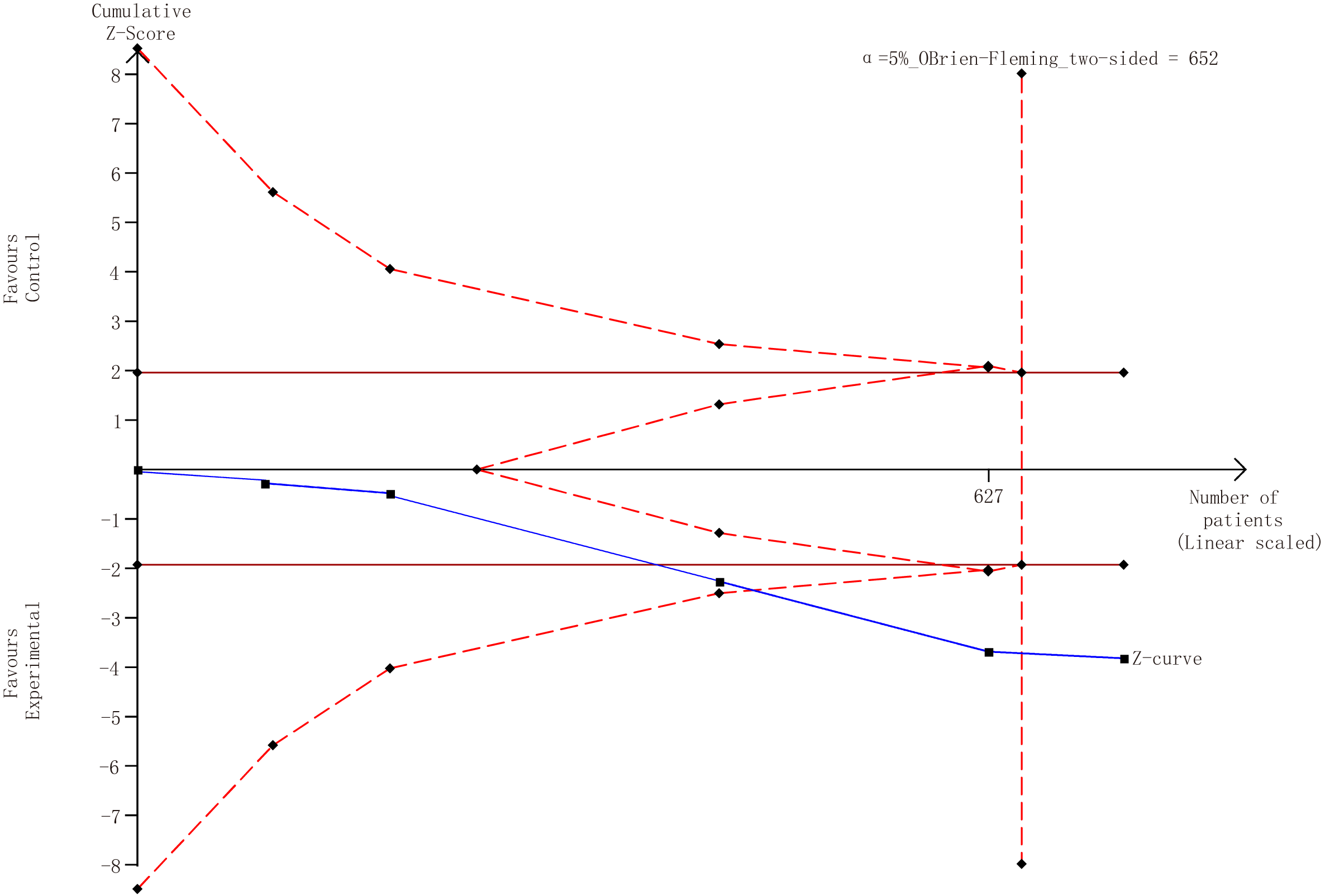
**

**eFigure 38. Trial sequential analysis of hormone therapy for quality of life**

**
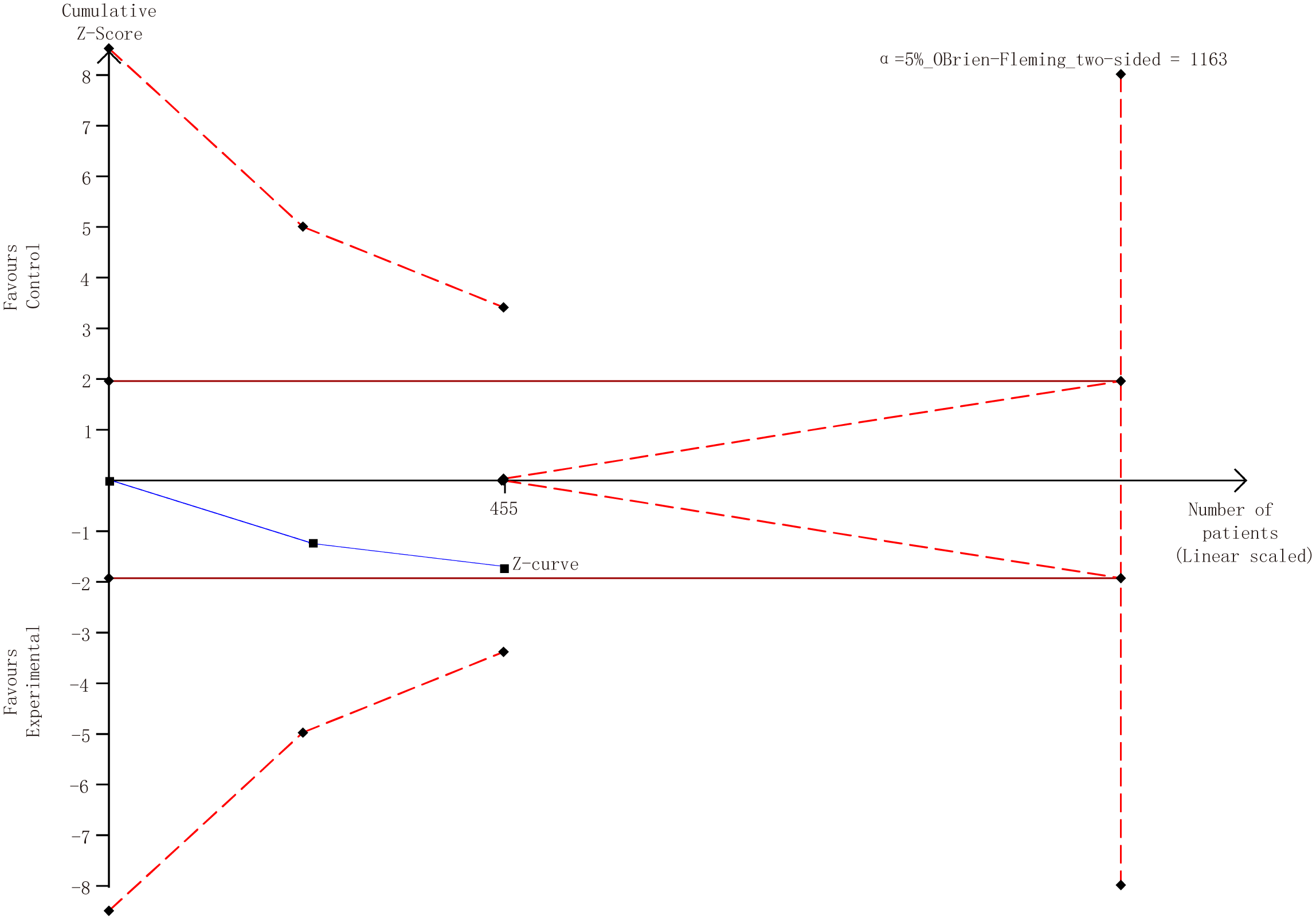
**

**eFigure 39. Trial sequential analysis of hormone therapy for well-being**

**
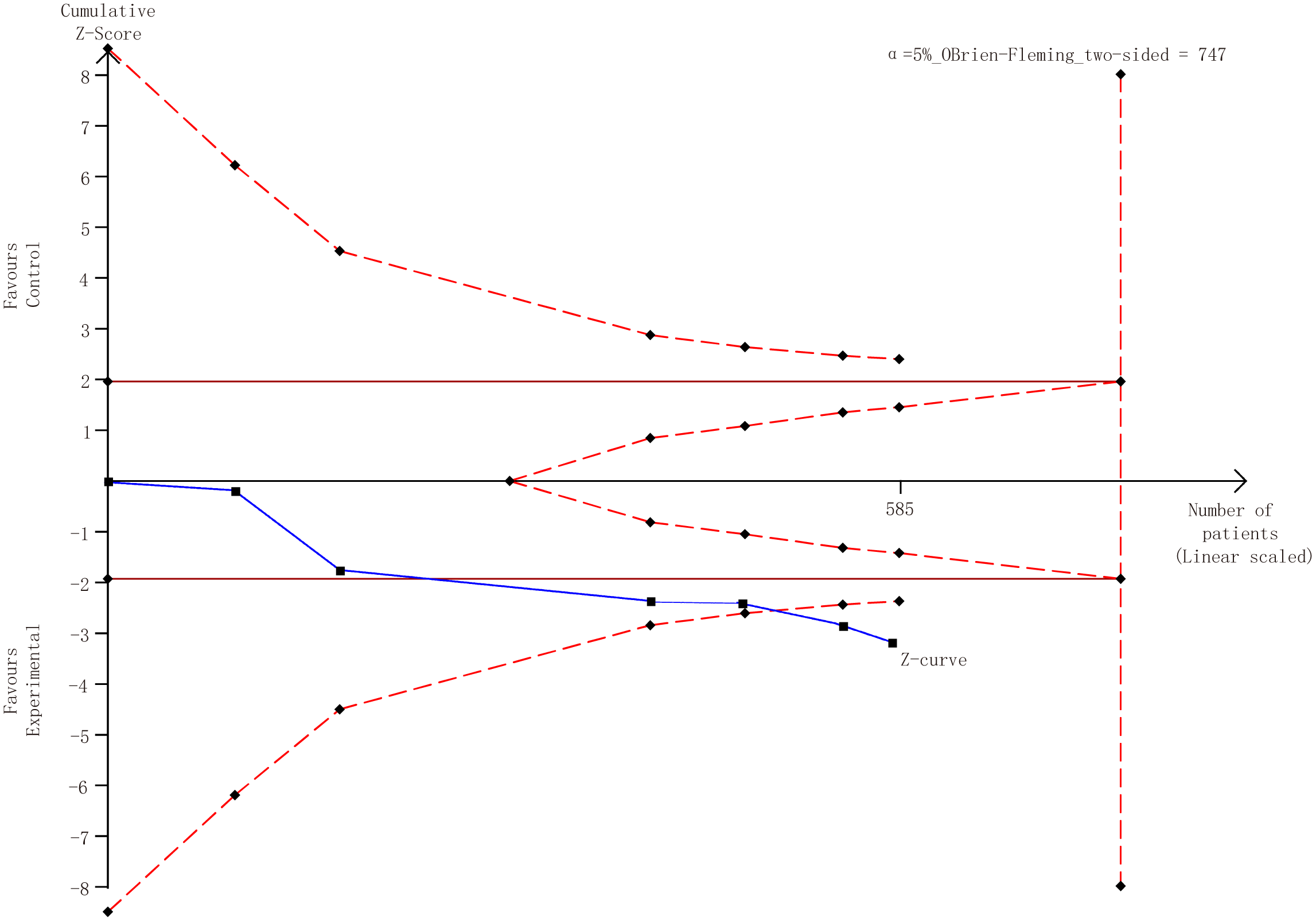
**

**eFigure 40. Trial sequential analysis of phytoestrogens for mood**

**
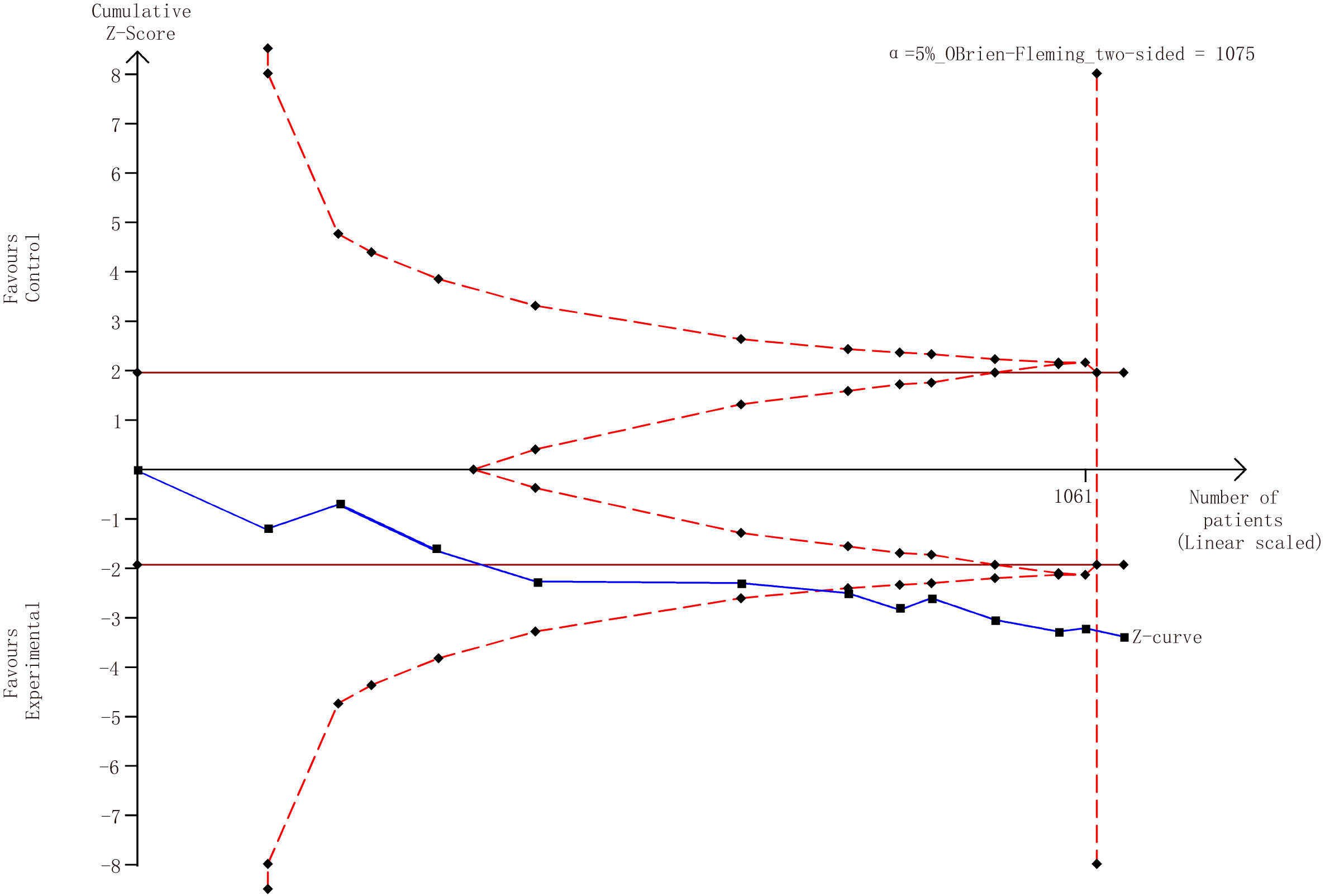
**

**eFigure 41. Trial sequential analysis of phytoestrogens for depression**

**
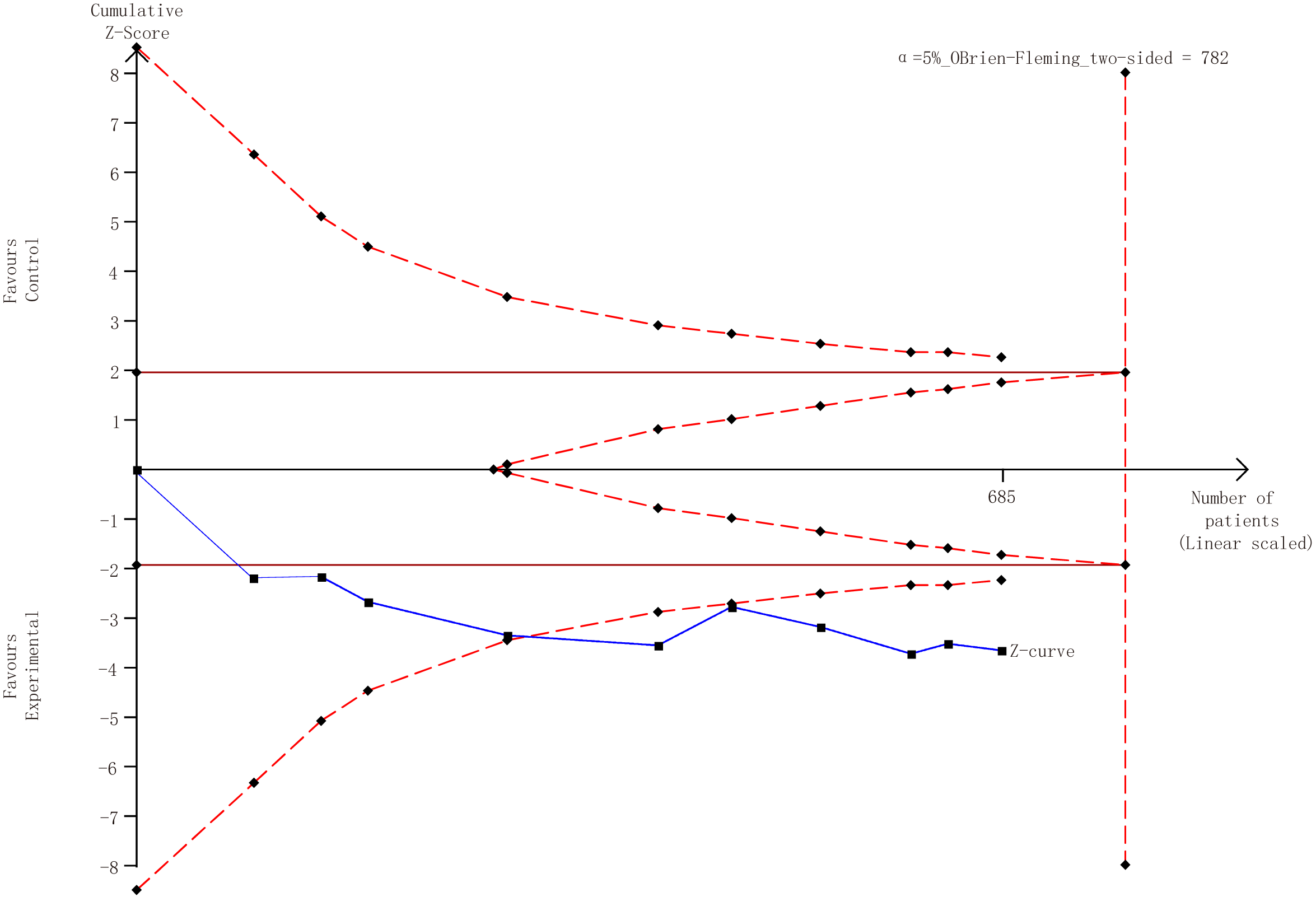
**

**eFigure 42. Trial sequential analysis of phytoestrogens for anxiety**

**
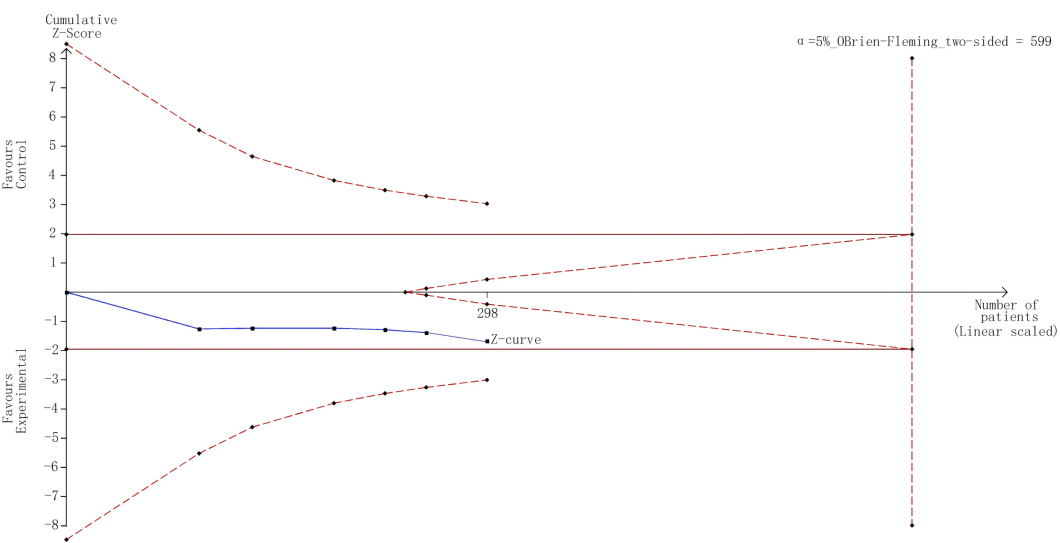
**

**eFigure 43. Trial sequential analysis of phytoestrogens for sleep**

**
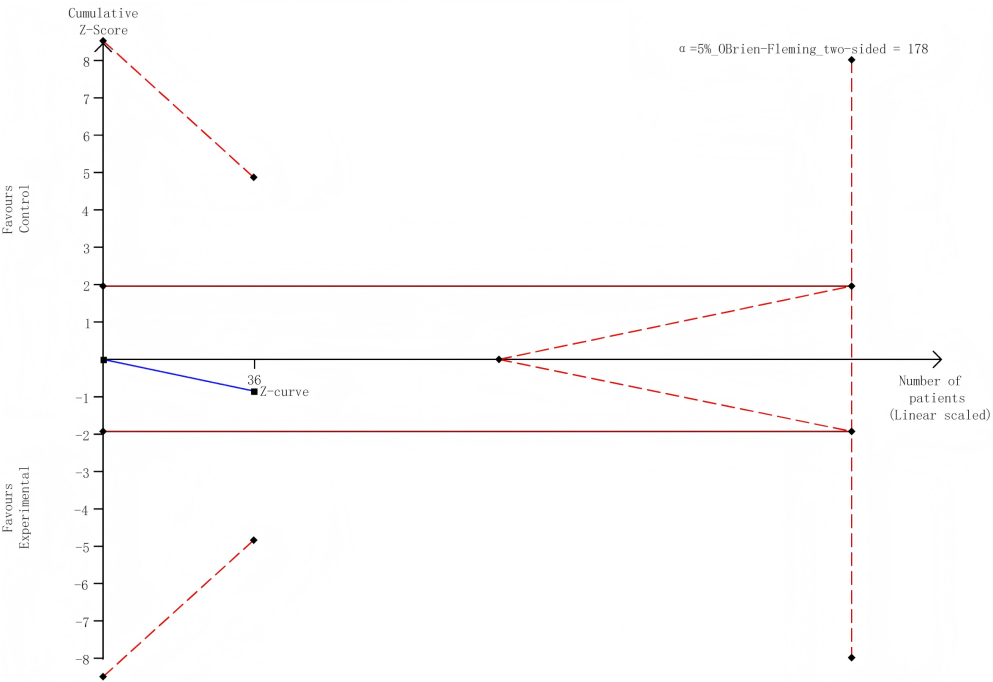
**

**eFigure 44. Trial sequential analysis of phytoestrogens for stress**

**
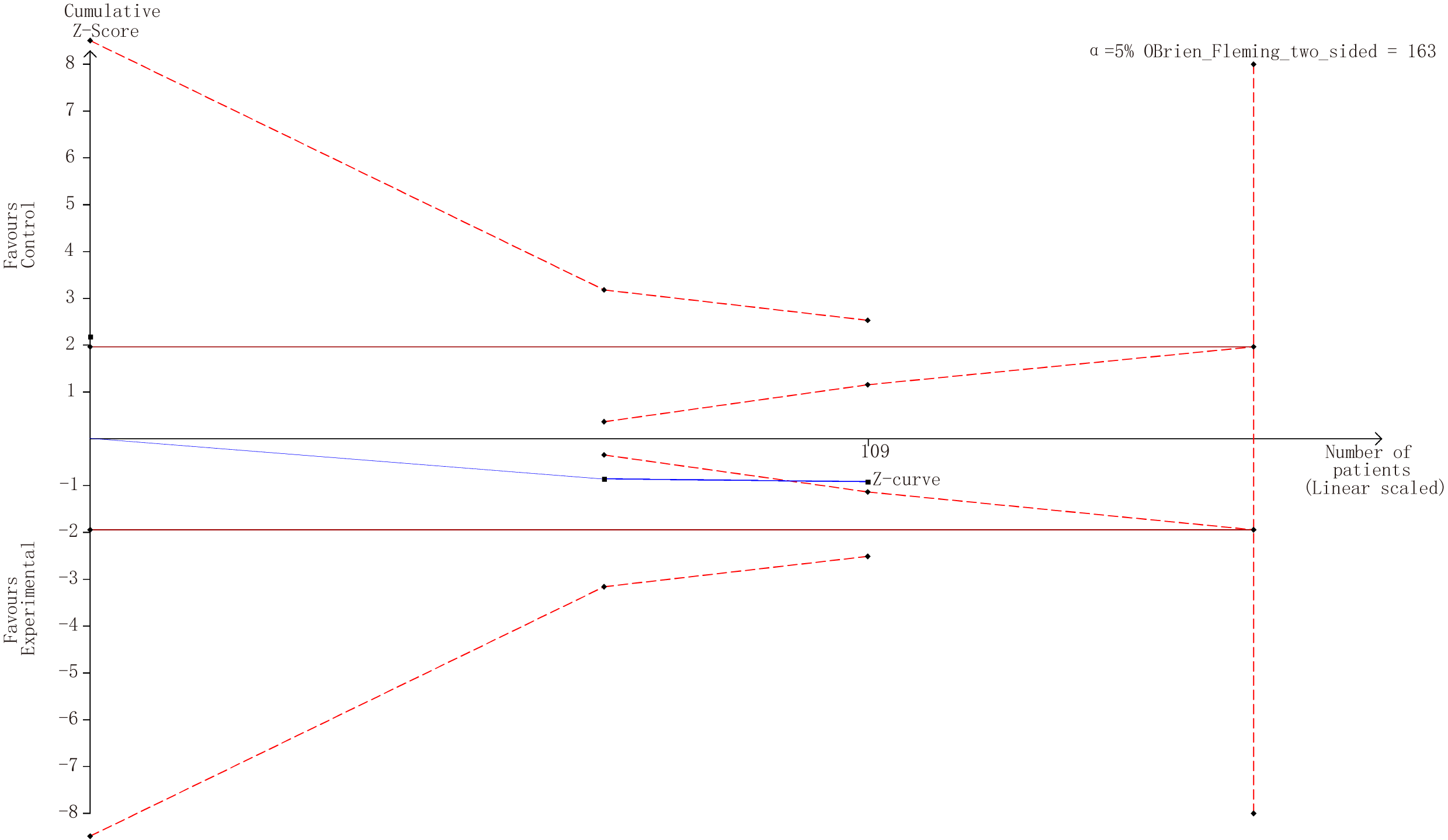
**

**eFigure 45. Trial sequential analysis of phytoestrogens for anger**

**
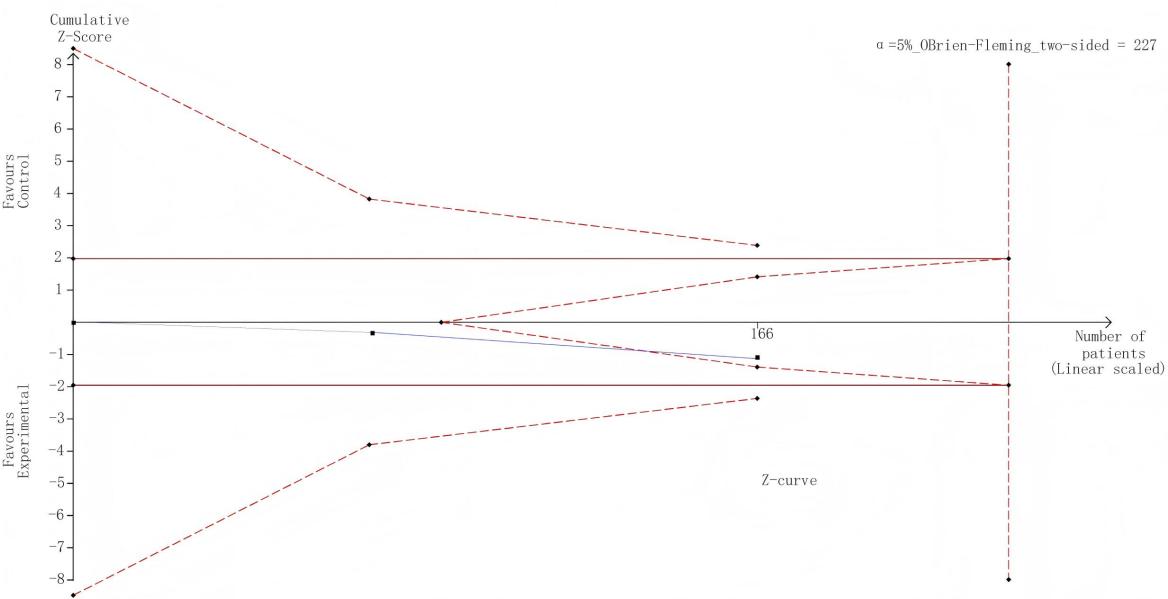
**

**eFigure 46. Trial sequential analysis of phytoestrogens for phobic**

**
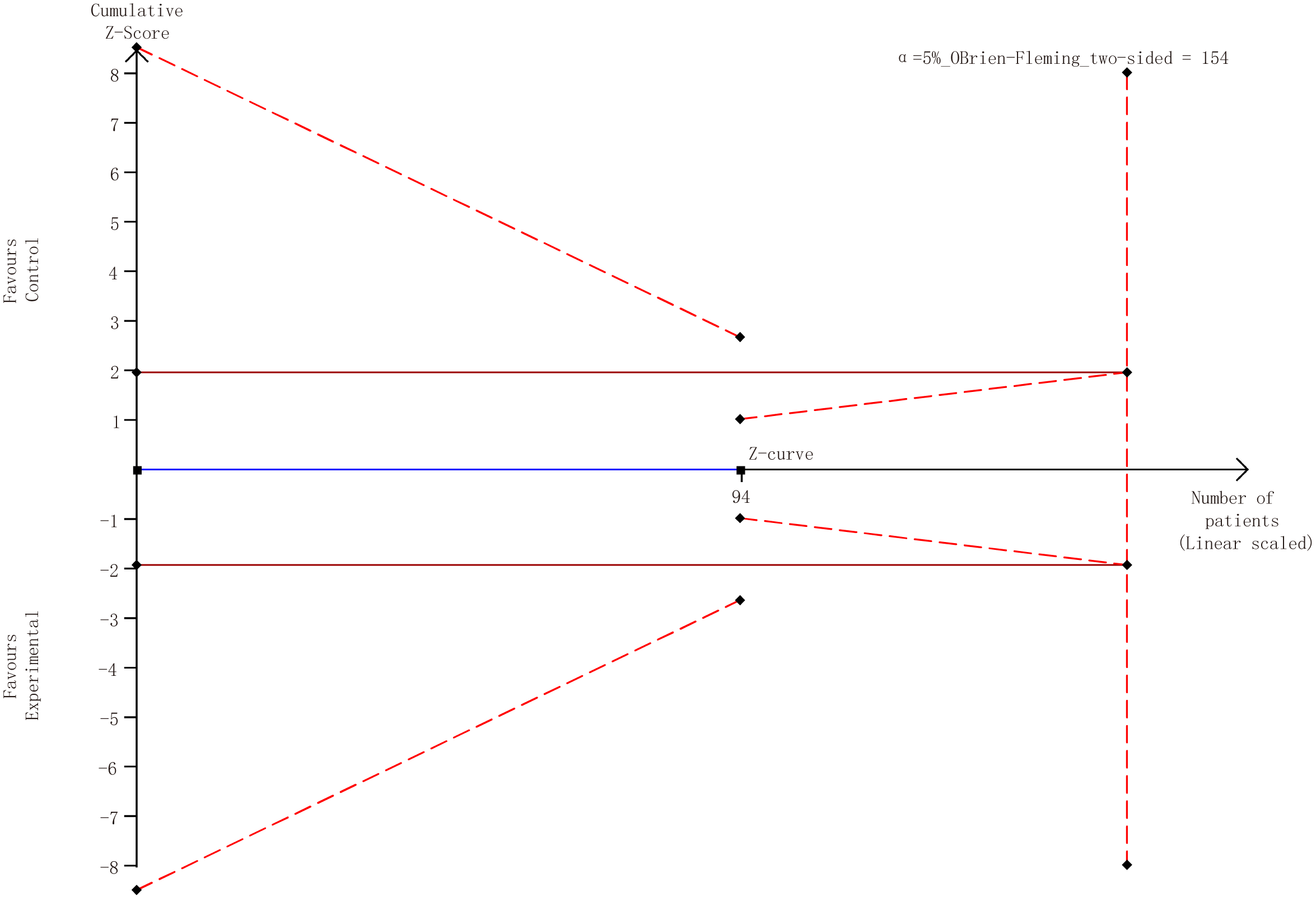
**

**eFigure 47. Trial sequential analysis of phytoestrogens for well-being**
